# Correction: Health worker compliance with severe malaria treatment guidelines in the context of implementing pre-referral rectal artesunate in the Democratic Republic of the Congo, Nigeria, and Uganda: An operational study

**DOI:** 10.1371/journal.pmed.1004234

**Published:** 2023-04-26

**Authors:** Aita Signorell, Phyllis Awor, Jean Okitawutshu, Antoinette Tshefu, Elizabeth Omoluabi, Manuel W. Hetzel, Proscovia Athieno, Joseph Kimera, Gloria Tumukunde, Irene Angiro, Jean-Claude Kalenga, Babatunde K. Akano, Kazeem Ayodeji, Charles Okon, Ocheche Yusuf, Giulia Delvento, Tristan T. Lee, Nina C. Brunner, Mark J. Lambiris, James Okuma, Nadja Cereghetti, Valentina Buj, Theodoor Visser, Harriet G. Napier, Christian Lengeler, Christian Burri

In the Author Summary, there is an error in the second sentence. The correct sentence is: In places where injectable treatment is not available, the World Health Organization (WHO) malaria guidelines recommend that children below 6 years of age be administered a single dose of rectal artesunate (RAS) before being referred to a higher-level health facility for parenteral treatment completed with a full course of artemisinin-based combination therapy (ACT).
