# Supplementary material for: Health worker compliance with severe malaria treatment guidelines in the context of implementing pre-referral rectal artesunate in the Democratic Republic of the Congo, Nigeria, and Uganda: An operational study
Source: PLoS Med. 2023 Feb 21;20(2):e1004189. doi: 10.1371/journal.pmed.1004189 (PMC9990943; doi:10.1371/journal.pmed.1004189)
Supplement: S1 File — (DOCX) [file pmed.1004189.s001.docx]

**Study Protocol**

**Community access to rectal artesunate for malaria (CARAMAL):**

**observational research in Nigeria, Uganda and DR Congo**

| **Protocol Number** | P 001-17-1.2 | | |
| --- | --- | --- | --- |
| **Version Number** | 1.2 | **Document Date** | 09.02.2018 |
| **Sponsor Contact** | Swiss Tropical and Public Health Institute  Socinstrasse 57  4051 Basel  Switzerland | | |
| **Principal Investigator** | Prof. Christian Burri  Socinstrasse 57  4051 Basel  Switzerland | | |
| **Funding Agency** | Unitaid  Chemin de Blandonnet 1  1214 Vernier  Switzerland | | |

*The information contained in this document is confidential. It is intended solely for the*

*Investigators, potential Investigators, consultants, or applicable Independent Ethics Committees and Regulatory Authorities. It is understood that this information will not be disclosed to others without prior written authorisation from the Sponsor, except where required by applicable local laws.*

# General Information

1. List of Investigators and other persons involved

| **Title** | **Names** | **Institution** | **Position** | **Function in study** |
| --- | --- | --- | --- | --- |
| Prof. | Christian Burri | Swiss TPH | Head, Division of Medicines Research, Department of Medicine | PI |
| Prof. | Christian Lengeler | Swiss TPH | Head, Health Intervention Unit, Department of Epidemiology and Public Health | Co-PI |
| PD Dr. | Manuel Hetzel | Swiss TPH | Scientific Project Leader | Co-Investigator |
| Dr. | Aita Signorell | Swiss TPH | Clinical Research Scientist | Co-Investigator |
| Prof. | Thomas Smith | Swiss TPH | Head, Infectious Disease Modelling Unit | Statistician |
| Prof. | Antoinette Tshefu | Kinshasa School of Public Health, University of Kinshasa | Professor | Sub-Investigator |
| Dr. | Phyllis Awor | Makerere University School of Public Health | Research Fellow | Sub-Investigator |
| Dr. | Elizabeth Omoluabi | Akena Associates Ltd. | Director | Sub-Investigator |

1. Signatures (adapt according to study)

**Authorized Sponsor representative**

| Signature |  |  |
| --- | --- | --- |
| Name | Prof. Christian Burri | Date of Signature (dd/mm/yy) |
| Title | Head, Division of Medicines Research, Department of Medicine | |
| Institution | Swiss Tropical and Public Health Institute | |
| Address | Socinstrasse 57  4051 Basel, Switzerland | |
| Phone |  | |

**Statistician**

| Signature |  |  |
| --- | --- | --- |
| Name | Prof. Thomas Smith | Date of Signature (dd/mm/yy) |
| Title | Head, Infectious Disease Modelling Unit | |
| Institution | Swiss Tropical and Public Health Institute | |
| Address | Socinstrasse 57  4051 Basel, Switzerland | |
| Phone |  | |

Principal Investigator(s)

- I have read this protocol and agree that it contains all necessary details for carrying out this study. I will conduct the study as outlined herein and will complete the study within the time designated.
- I will ensure that all individuals and parties contributing to this study are qualified and I will implement procedures to ensure integrity of study tasks and data.
- I will provide copies of the protocol and all pertinent information to all individuals responsible to me who assist in the conduct of this study. I will discuss this material with them to ensure they are fully informed regarding the drug and the conduct of the study.
- I will use only the informed consent forms approved by the Sponsor or its representative and will fulfil all responsibilities for submitting pertinent information to the Independent Ethics Committees responsible for this study.
- I agree that the Sponsor or its representatives shall have access to any source documents from which Case Report Form information may have been generated.

**Principal Investigator**

| Signature |  |  |
| --- | --- | --- |
| Name | Prof. Christian Burri | Date of Signature (dd/mm/yy) |
| Title | Head, Division of Medicines Research, Department of Medicine | |
| Institution | Swiss Tropical and Public Health Institute | |
| Address | Socinstrasse 57  4051 Basel, Switzerland | |
| Phone |  | |

**Co-Principal Investigator**

| Signature |  |  |
| --- | --- | --- |
| Name | Prof. Christian Lengeler | Date of Signature (dd/mm/yy) |
| Title | Head, Health Intervention Unit, Department of Epidemiology and Public Health | |
| Institution | Swiss Tropical and Public Health Institute | |
| Address | Socinstrasse 57  4051 Basel, Switzerland | |
| Phone |  | |

**Sub-investigator Democratic Republic of the Congo**

| Signature |  |  |
| --- | --- | --- |
| Name | Prof. Antoinette Tshefu | Date of Signature (dd/mm/yy) |
| Title | Professor | |
| Institution | Kinshasa School of Public Health, University of Kinshasa | |
| Address | Kinshasa, DRC | |
| Phone |  | |

**Sub-investigator Nigeria**

| Signature |  |  |
| --- | --- | --- |
| Name | Dr. Elizabeth Omoluabi | Date of Signature (dd/mm/yy) |
| Title | Director | |
| Institution | Akena Associates Ltd. | |
| Address | Road F, House 20, CITEC; Abuja; Nigeria | |
| Phone |  | |

**Sub-investigator Uganda**

| Signature |  |  |
| --- | --- | --- |
| Name | Dr. Phyllis Awor | Date of Signature (dd/mm/yy) |
| Title | Research Fellow | |
| Institution | Makerere University School of Public Health | |
| Address | Mulago Hospital Complex, Kampala, Uganda | |
| Phone |  | |

1. Table of contents

[1 General Information 2](#_Toc126762185)

[I. List of Investigators and other persons involved 2](#_Toc126762186)

[II. Signatures (adapt according to study) 3](#_Toc126762187)

[III. Table of contents 6](#_Toc126762188)

[IV. Abbreviations / Glossary of terms 8](#_Toc126762189)

[V. Synopsis 10](#_Toc126762190)

[2 Background information 20](#_Toc126762191)

[2.1 Malaria 20](#_Toc126762192)

[2.2 Treatment of malaria 20](#_Toc126762193)

[2.3 Rectal artesunate as pre-referral treatment 20](#_Toc126762194)

[2.4 Project justification 21](#_Toc126762195)

[2.4.1 Roll-out of rectal artesunate in DR Congo, Nigeria and Uganda 21](#_Toc126762196)

[3 Overall goal, objectives and outcomes 23](#_Toc126762197)

[3.1 Overall goal 23](#_Toc126762198)

[3.2 Study objectives and research questions 23](#_Toc126762199)

[3.2.1 Objectives 23](#_Toc126762200)

[3.2.2 Research questions 23](#_Toc126762201)

[3.3 Study outcomes 24](#_Toc126762202)

[3.3.1 Monitoring of Artemisinin resistance 25](#_Toc126762203)

[4 Study sites 27](#_Toc126762204)

[4.1.1 Integrated community case management and RAS 28](#_Toc126762205)

[4.1.2 Selection of study area within each country 28](#_Toc126762206)

[5 Project design and data collection procedures 30](#_Toc126762207)

[5.1 Activity 1: Patient surveillance system 31](#_Toc126762208)

[5.1.1 Study population 31](#_Toc126762209)

[5.1.2 Procedures and instruments 32](#_Toc126762210)

[5.2 Activity 2: Health care provider survey 38](#_Toc126762211)

[5.2.1 Study population 38](#_Toc126762212)

[5.2.2 Procedures and instruments 38](#_Toc126762213)

[5.2.3 Rapid Assessment of referral facilities 39](#_Toc126762214)

[5.3 Activity 3: Household survey 39](#_Toc126762215)

[5.3.1 Study population 39](#_Toc126762216)

[5.3.2 Procedures and instruments 40](#_Toc126762217)

[5.4 Activity 4: Economic evaluation study 41](#_Toc126762218)

[5.5 Activity 5: Routine monitoring of process indicators 41](#_Toc126762219)

[5.6 Biological sample collection and diagnostic tests 42](#_Toc126762220)

[5.7 Environmental temperature 42](#_Toc126762221)

[5.8 Measures to minimize bias 43](#_Toc126762222)

[5.9 Study duration 43](#_Toc126762223)

[5.9.1 Early termination of the study 44](#_Toc126762224)

[6 Selection of the study participants 45](#_Toc126762225)

[6.1 Recruitment 45](#_Toc126762226)

[6.2 Inclusion criteria 45](#_Toc126762227)

[6.3 Exclusion criteria 45](#_Toc126762228)

[7 Description of data management 47](#_Toc126762229)

[7.1 Data collection 47](#_Toc126762230)

[7.2 Data management system 47](#_Toc126762231)

[7.3 Data security, access, archiving and back up 48](#_Toc126762232)

[8 Statistics 49](#_Toc126762233)

[8.1 Hypothesis 49](#_Toc126762234)

[8.2 Determination of sample size 49](#_Toc126762235)

[8.2.1 Sample size calculation for case fatality ratio (CFR) 49](#_Toc126762236)

[8.2.2 Sample size calculation for successful completion of referral 51](#_Toc126762237)

[8.2.3 Sample size calculation for Health care provider surveys 51](#_Toc126762238)

[8.2.4 Sample size calculation for household surveys: treatment seeking from CHF/primary health facility 52](#_Toc126762239)

[8.3 Description of statistical methods 52](#_Toc126762240)

[8.3.1 Descriptive analysis of routine M&E data 52](#_Toc126762241)

[8.3.2 Inference of the effects of the RAS roll-out 52](#_Toc126762242)

[8.4 Handling of data 53](#_Toc126762243)

[9 Duties of the Investigator 54](#_Toc126762244)

[9.1 Investigator’s confirmation 54](#_Toc126762245)

[9.2 Safety reporting 54](#_Toc126762246)

[9.3 Project management 54](#_Toc126762247)

[10 Ethical considerations 56](#_Toc126762248)

[10.1 Independent Ethics Committee (IEC) 56](#_Toc126762249)

[10.2 Evaluation of the risk-benefit ratio 56](#_Toc126762250)

[10.2.1 Risk categorisation 56](#_Toc126762251)

[10.2.2 Risks to participants 57](#_Toc126762252)

[10.2.3 Potential Benefits of the Proposed Research 57](#_Toc126762253)

[10.3 Participant information and consent 57](#_Toc126762254)

[10.4 Participant confidentiality 59](#_Toc126762255)

[10.5 Participants requiring particular protection 59](#_Toc126762256)

[10.6 Damage coverage 60](#_Toc126762257)

[10.7 Participant compensation 60](#_Toc126762258)

[10.8 Other aspects 60](#_Toc126762259)

[11 Quality control and quality assurance: description of measures 61](#_Toc126762260)

[11.1 Risk management 61](#_Toc126762261)

[11.1.1 Risk identification, assessment and mitigation 61](#_Toc126762262)

[11.2 Quality control 62](#_Toc126762263)

[11.3 Translations - Reference language 62](#_Toc126762264)

[12 Dissemination of results and publication policy 63](#_Toc126762265)

[13 Funding and support 63](#_Toc126762266)

[14 References 64](#_Toc126762267)

1. Abbreviations / Glossary of terms

| ACT | Artemisinin-based Combination Therapy |
| --- | --- |
| AE | Adverse Event |
| ANC | Antenatal Care |
| AQUAMAT | African Quinine Artesunate Malaria Trial |
| CARAMAL | Community Access to Rectal Artesunate for Malaria |
| CFR | Case Fatality Ratio |
| CHAI | Clinton Health Access Initiative |
| CHW | Community Health Worker |
| CNES | Comité National d’Ethique de la Santé |
| CRF | Case Report Form |
| DHIS2 | District Health Information System 2 |
| DQA | Data Quality Audit |
| DRC | Democratic Republic of the Congo |
| EDC | Electronic Data Capture |
| EGEP | Essentials of Good Epidemiological Practice |
| EKNZ | Ethikkommission Nordwest- und Zentralschweiz |
| ERP | Expert Review Panel |
| GCP | Good Clinical Practice |
| Hb | Haemoglobin |
| HCPS | Health Care Provider Survey |
| HH | Household |
| HMIS | Health Management Information System |
| iCCM | integrated Community Case Management |
| ICF | Informed Consent Form |
| IEC | Independent Ethics Committee |
| IPTi | Intermittent Preventive Treatment in infants |
| IPTp | Intermittent Preventive Treatment in pregnancy |
| IRB | Institutional Review Board |
| ISMO | Improving Severe Malaria Outcomes |
| ITN | Insecticide-Treated Nets |
| Kofam | Koordinationsstelle für Forschung am Menschen (Federal Office of Public Health’s portal for human research in Switzerland) |
| LGA | Local Government Area |
| M&E | Monitoring and Evaluation |
| min | Minutes |
| MMV | Medicines for Malaria Venture |
| mRDT | Malaria Rapid Diagnostic Test |
| MUAC | Mid-Upper-Arm Circumference |
| ODK | Open Data Kit |
| PQ | WHO prequalification |
| PSS | Patient Surveillance System |
| RAS | Rectal Artesunate |
| REC | Research Ethics Committee |
| Q | Quarter |
| QA | Quality-Assured |
| SAE | Serious Adverse Event |
| Swiss TPH | Swiss Tropical and Public Health Institute |
| Tmax | Time to maximal plasma concentration |
| UNCST | Uganda National Council for Science & Technology |
| UNICEF | United Nations Children’s Fund |
| VHT | Village Health Team |
| Wi-Fi | Technology for wireless local area networking |
| WHO | World Health Organization |
| WHO-PQP | WHO - Prequalification of Medicines Programme |

1. Synopsis

| **Sponsor:** | Swiss Tropical and Public Health Institute |
| --- | --- |
| **Study Title:** | **Community access to rectal artesunate for malaria (CARAMAL):**  **observational research in Nigeria, Uganda and DR Congo** |
| **Short Title/Study ID:** | **Caramal** |
| **Protocol Version and Date:** | Version 1.1, 28.11.2017 |
| **Investigators:** | **Principal Investigators:**  Prof. Christian Burri  Prof. Christian Lengeler  **Co-Investigators:**  PD Dr. Manuel Hetzel  Dr. Aita Signorell  **Sub-Investigators:**  Prof. Antoinette Tshefu (DRC)  Dr. Elizabeth Omoluabi (Nigeria)  Dr. Phyllis Awor (Uganda) |
| **Study Category:** | Non-clinical research project (including persons - data or samples)  Collection of health related personal data. Data will be coded. |
| **Background and Rationale:** | Malaria is one of the leading causes of illness, death, and lost economic productivity globally. While the successful scale-up and use of critical commodities such as insecticide-treated nets (ITNs) and artemisinin-based combination therapies (ACTs) have led to a 62% decline in malaria-related mortality between 2000 and 2015, malaria still results in over 400,000 deaths each year, most of which are in children under 5 years of age and pregnant women [1]. The heaviest malaria burden continues to be borne by sub-Saharan African countries which accounted for an estimated 90% of malaria cases and 92% of malaria deaths in 2015 [1].  Oral artemisinin-based combination therapy (ACT) is currently the most effective and recommended treatment for episodes of uncomplicated malaria [2]. Patients with severe malaria on the other hand (including infants, pregnant women in all trimesters and lactating women) should first be treated with intravenous or intramuscular artesunate for at least 24 hours until they can tolerate a full 3-day oral treatment course of ACT [2].  Many episodes of severe malaria occur in remote locations in which access to health services is limited, treatment for severe malaria, particularly parenteral therapy is not available, and long delays in referring patients to an appropriate health facility are common [3]. Without effective pre-referral treatment that can be administered at the community level, many cases of severe malaria will result in death.  Where parenteral treatment of severe malaria is not available, the World Health Organization (WHO) recommends treating children less than 6 years old with a single rectal dose of 10 mg artesunate per kilogram of body weight [2] prior to referral. It rapidly (i.e. within 24 h) clears 90% or more of malaria parasites [4] and in children less than 6 years old who cannot reach a facility in less than six hours, it can reduce the risk of death or permanent disability by up to 50% [4]. After the administration of rectal artesunate (RAS), the child should be referred immediately to an appropriate facility where the full package of care for severe malaria can be provided.  Currently, 16 countries in Africa have included the use of pre-referral RAS in their treatment policies, but guidelines for use vary widely across these countries and often they do not align with the WHO recommendation. The only study that formally evaluated the use of pre-referral RAS in Bangladesh, Ghana and Tanzania found a reduction in mortality for children under 6 years, but also an indication that in Asia the use of the product might increase mortality in children older than 6 years [3]. Despite misalignments and lack of operational guidance, countries have begun to procure and implement RAS. Widespread use of this product without alignment with WHO recommendations raises substantial concerns, including regarding its inappropriate use as a monotherapy against both severe and uncomplicated malaria.  With the impending availability of quality-assured rectal artesunate (QA RAS) and countries poised to scale-up this intervention, it is critical to investigate the safe and effective implementation of RAS in existing community-based case management systems, as part of a continuum of care for severe malaria patients. The pilot roll-out of QA RAS in the Democratic Republic of the Congo (DRC), Nigeria and Uganda provides a unique and timely opportunity to generate high-quality evidence applicable to a wide range of African settings.  Unitaid will support a pilot roll-out of QA RAS in selected areas of the three countries linked to the research project outlined in this protocol. In this context, QA RAS will be procured through UNICEF as suppositories containing 100 mg of artesunate, and implemented through existing iCCM schemes.  The pilot roll-out will be accompanied by the multi-country observational research project described in this protocol. It is to our best knowledge the first and only current study to investigate key questions linked to the large-scale roll-out of pre-referral RAS.  The Community access to rectal artesunate for malaria (CARAMAL) project will be implemented by a consortium comprised of the Clinton Health Access Initiative (CHAI), UNICEF, the Swiss Tropical and Public Health Institute (Swiss TPH) and three Country Research Partners:   - **CHAI** as the lead grantee of the RAS implementation project is responsible for the overall governance of the project. - **UNICEF** will procure QA RAS and support its introduction into functional local iCCM systems according to existing guidelines, ensuring that all personnel within the continuum of care are prepared and supported to adequately administer QA RAS. - **Swiss TPH** is responsible for generating and disseminating scientific evidence as described in detail in this protocol. - **Country Research Partners** will be responsible for the local implementation of all research (non-routine) data collection and country-level analyses:   - DRC: **Kinshasa School of Public Health, University of Kinshasa**   - Nigeria: **Akena Associates Ltd.**   - Uganda: **School of Public Health, Makerere University** |
| **Objective(s):** | The overall goal of the CARAMAL project is to contribute to reducing malaria mortality in children globally by improving the community management of suspected severe malaria cases. The project will contribute to this goal by advancing the development of operational guidance to catalyse effective and appropriate scale-up of QA RAS as pre-referral treatment of severe malaria.  Accompanying the pilot roll-out of QA RAS by UNICEF, the CARAMAL project will test whether it is feasible to introduce QA RAS into established integrated community case management (iCCM) platforms with only minimal additional supportive interventions and with minimal unintended consequences such as inappropriate use as artemisinin monotherapy.  Through the research activities described in detail below, the CARAMAL project aims to answer the following **research questions**:   1. What are the **minimal requirements** of a community case management system **to ensure that RAS is an effective part of the continuum of care** from the community to a referral facility (defined as a health care facility equipped for inpatient care of severe malaria)? 2. What are the **unintended consequences** of scaled implementation, such as adverse drug reactions, unforeseen costs [5], or unforeseen issues in treatment of malaria at all levels of care, and how can they be addressed? 3. Is there any **use of RAS beyond the recommended guidelines**, including full treatment of severe cases with RAS at community level, and the treatment of uncomplicated malaria with RAS? 4. Can the introduction of pre-referral QA RAS **reduce severe malaria case fatality ratio** over time under real-world operational circumstances in three distinct settings? 5. What are the **costs and cost-effectiveness** of community and peripheral health facility based RAS? |
| **Outcome:** | **Key indicators:**   \| **Research question** \| **Indicator** \| **Source** \| **Indicator description** \| **Frequency** \| \| --- \| --- \| --- \| --- \| --- \| \| **I** \| QA RAS coverage \| M&E, HCPS \| Proportion of trained and functional CHW and primary HF who provide QA RAS \| Annually \| \| RAS availability \| M&E, HCPS \| Proportion of CHW/primary HF with RAS in stock \| Annually \| \| Functional referral facilities \| HCPS \| Proportion of referral health facilities that have the capacity to manage severe malaria in children in line with global guidance \| Annually \| \| Supervision \| M&E, HCPS \| Proportion of CHW/primary HF that received at least one supervisory visit in the past 3 months \| Annually \| \| **I + II** \| Adverse events \| PSS \| Frequency of passively reported adverse events after RAS administration \| Continuous \| \| Delayed haemolytic anaemia \| PSS \| Frequency of delayed haemolytic anaemia within 28 days after RAS administration \| Continuous \| \| **II** \| Treatment seeking \| HHS \| Proportion of children <5 years with a recent history of fever (mild or severe) who attend CHW/primary HF \| Annually \| \| CHW attendance \| PSS \| Proportion of children <5 years with a recent history of fever (mild or severe) who attend CHW/primary HF \| Annually \| \| RAS acceptability among health workers \| HCPS \| Acceptability of pre-referral RAS among health workers \| Annually \| \| RAS acceptability in community \| HHS \| Acceptability of pre-referral RAS among caretakers \| Annually \| \| Referral \| PSS \| Proportion of children <5 years with severe febrile illness seen by CHW/primary HF who completed referral \| Continuous \| \| Direct reporting \| PSS \| Number of children <5 years with severe febrile illness who report directly to RHF \| Continuous \| \| **I + III** \| Pre-referral treatment \| PSS \| Proportion of children <5 years with severe febrile illness managed pre-referral according to guidelines \| Continuous \| \| Post-referral treatment \| PSS \| Proportion of children <5 years with severe febrile illness managed post-referral according to guidelines \| Continuous \| \| **IV** \| Case fatality ratio \| PSS \| Proportion of children <5 years with severe febrile illness seen by CHW/primary HF that resulted in death within 28 days \| Continuous \| \| Malaria infection on day 28 \| PSS \| Proportion of children <5 years with severe febrile illness without parasites on day 28 \| Continuous \| \| Successful referral \| PSS \| Proportion of children <5 years with severe febrile illness seen by CHW/primary HF who completed referral to a referral health facility \| Continuous \| \| **V** \| Costs \| EES \| Total financial cost of managing severe case at community level.  Marginal financial cost of adding RAS to the management. \| Continuous  From project records \| \| Cost-effectiveness \| PSS EES \| Financial cost of RAS interventions per death averted. \| PSS and project records \| \| **Exploratory / contextual** \| Danger signs \| HHS \| Proportion of children <5 years with a recent history of fever who reported danger signs (signs of 'severe febrile illness'). \| Annually \| \| Malaria prevalence \| HHS \| Proportion of children <5 years infected with malaria parasites \| Annually \|   PSS = Patient surveillance system (Activity 1); HCPS = Health care provider survey (Activity 2); HHS = Household survey (Activity 3); EES = Economic evaluation study (Activity 4); HF = Health facility; M&E = routine M & E activity (Activity 5) |
| **Study Design:** | The CARAMAL project has been designed as a multi-country operational research study implemented in three highly malaria-endemic countries. It will be based on a before-and-after plausibility design [6] aligned with the roll-out of QA RAS through established community-based health care provider systems.  **Activity 1:** A **patient surveillance** **system** **(PSS)** to assess severe febrile illness/suspected severe malaria incidence, case fatality rate, and related clinical patterns, diagnoses, treatment and treatment outcomes from first contact to the point of recovery or death (baseline and after RAS roll-out)  **Activity 2:** **Health care provider surveys** **(HCPS)** to establish the availability and uptake of QA RAS at all levels, and health providers’ knowledge, attitudes and practices towards RAS (baseline and after RAS roll-out)  **Activity 3:** **Household surveys** **(HHS)** to assess treatment seeking, caretakers’ knowledge and attitudes towards RAS, and malaria intervention coverage at community level (baseline and after RAS roll-out)  **Activity 4:** An **economic evaluation** to assess the costs and incremental cost-effectiveness of implementing RAS at community level compared to the current standard of care  **Activity 5:** **Routine monitoring of process indicators** **along the entire case management chain** to continuously assess implementation progress of RAS as contextual information for other outcome indicators |
| **Study Population:** | **Activity 1, Patient surveillance system:**  The PSS will include all children <5 years of age seeking care for a current/recent febrile illness episode at the level of community based care providers in the study areas, including CHWs and primary health facilities. All children diagnosed at that level with severe febrile illness / suspected severe malaria will then be enrolled in the PSS and tracked at the referral health facility and at the child’s home 28 days after initial diagnosis. In addition, all children <5 years of age seeking care for a severe febrile illness episode directly at the referral facility will be enrolled to evaluate treatment seeking, diagnosis, treatment and disease outcome. These children will be excluded from the case fatality ratio analysis.  **Activity 2,** **Health care provider surveys:**  The sampling frame will include all registered providers at all levels operating in the study areas, including public and private providers. A simple random sampling approach stratified by provider level (CHW, primary health facility, etc.) will be used to select providers for the survey.  **Activity 3,** **Household surveys**:  Household surveys will include randomly sampled households in the study areas. Households will be selected using a two-stage random sampling approach (village-household) whereas the sampling frames will consist of all villages in the study area and all households in the village, respectively.  In each household, the household heads and parents/caretakers of children < 5 years of age will be eligible to participate. |
| **Measurements and Procedures:** | **Activity 1, patient surveillance system:**   1. **CHW / primary health facility (first contact)**   According to established routine practice, the following procedures are performed: children attending a CHW or primary health facility undergo examination, treatment and referral procedures as per local guidelines. An mRDT is performed on all children with a history of or acute fever, including children with symptoms of severe febrile illness / children with danger signs. The children are then treated as per the applicable national guidelines. Enrolled children will be assigned a unique study ID.   1. **Referral facility**   Children with severe febrile illness / suspected severe malaria and either referred to or directly reporting to a referral facility in the study area will be registered upon arrival in the facility. The study nurse will monitor the case management of each registered patient and continuously enter study-specific details on diagnosis and treatment provided throughout the patient’s admission into an electronic case report form.   1. **At home (day 28)**   All children with severe febrile illness / suspected severe malaria and enrolled into the PSS by a CHW / primary health facility or at the referral health facility will be followed up at their home by a member of the research team. The primary purpose of this visit is to establish the health status of the child using a structured questionnaire. It will also include a section on the parent’s/caretaker’s experience and attitude towards the use of RAS. During the home follow-up a finger-prick blood sample will be collected from all of children for:  - mRDT  - Microcuvette sample for measuring haemoglobin (Hb) concentration  In case of the death of an enrolled child, the research team will attempt to conduct a verbal autopsy at a later stage.  **Activity 2,** **Health care provider surveys:**   1. **Health Care Provider Checklist**   A structured checklist completed to assess the availability of essential medical supplies (incl. RAS) and equipment, human resource capacity, infrastructure and documentation.   1. **Health Care Provider Questionnaire**   An interviewer administered questionnaire with questions pertaining to the health worker’s demographics, education and training, work experience and supervision, type and utility of any work-related training received, knowledge, attitudes and practices relevant to febrile case management (incl. diagnostic algorithm and (RAS) treatment guidelines) and intermittent preventive treatment in infants and pregnancy (IPTi, IPTp), experiences implementing malaria/febrile case management and prevention guidelines. More in-depth questions will be asked to health care workers administering RAS as well as different cadres of health workers providing post-referral treatment, focusing on aspects relevant to the implementation of pre-referral RAS and post-referral treatment of severe febrile illness.  **Activity 3,** **Household surveys**:  Three survey instruments will be completed with participating household heads and/or household members:   1. **Household Questionnaire**   A structured interviewer administered questionnaire to collect information about coverage and uptake of mosquito nets behaviour change campaigns and other malaria control interventions, alongside background demographic information on each household member as well as indicators of the household’s socio-economic status.   1. **Treatment Seeking Questionnaire**   A semi-structured interviewer administered questionnaire completed with randomly selected household members who are parents or caretakers of a child <5 years of age. Parents/caretakers of children who reported a febrile illness (irrespective of severity) in the two weeks prior to the survey will be interviewed about that specific illness episode. The form collects information about the signs and symptoms of the illness and subsequent treatment seeking behaviours, including sources of treatment and types (if any) of drugs administered. Parents/caretakers of children who did not experience a recent febrile illness will be asked about hypothetical care seeking behaviour based on specific vignettes (one scenario of mild and one scenario of severe febrile illness). The form furthermore collects information on the knowledge and attitudes of the parents/caretakers towards RAS. The parent/caretaker will be asked about previous experiences with RAS. Parents/caretakers without any previous experience with RAS will be asked about their attitudes based on a vignette.   1. **Prevalence Form / blood sample collection**   A finger prick blood sample will be taken from every household member below 5 years of age to assess changes in malaria prevalence and anaemia over time in the study areas. A short form will accompany each finger-prick blood sample, recording the individuals’ demographic details, recent travel history, intake of any medicine, the mRDT and Hb measurement result, and any treatment administered.  **Activity 4, Economic evaluation:**  Financial and non-financial economic costs will be collected as part of routine project records over the duration of the study. The evaluation will reflect multiple perspectives including individual (i.e. patient), societal, and health systems-level (i.e. government).  **Activity 5, Routine monitoring of process indicators:**  Programmatic records of the implementation of QA RAS by UNICEF will be continuously assessed, including:   1. RAS needs assessment data and orders 2. Reports from supportive supervision of CHWs 3. CHW monthly reports 4. CHW, health facility registers |
| **Number of Participants with Power Analysis:** | **Activity 1, Patient surveillance system:**  The minimum sample size of **6,032 cases of severe malaria in children < 5 years** over 24 months is based on the calculation of difference in proportions allowing the detection of a 30% decrease in case fatality following the roll-out of RAS with 80% power and α = 0.05. Based on the project time plan with 6 months of baseline and 18 months post-RAS (total 2 years, allocation ratio = 3), this translates into a minimum of 1508 cases at baseline and 4524 after RAS roll-out.  **Activity 2:** **Health care provider surveys:**   - **All referral health facilities** in the project areas - **All non-referral health facilities** in the study area (Uganda and DRC), while in Nigeria, a random sample of **40 facilities** will be included, allowing for the detection of a reduction of coverage indicators by 19 percentage points from a baseline value of 80% with a power of 80% and α = 0.05 - **40 community health workers** (Nigeria and Uganda), allowing for the detection of a reduction of coverage indicators by 19 percentage points from a baseline value of 80% with a power of 80% and α = 0.05. In DRC, **all CHW** will be included due to their smaller number (<40).   **Activity 3:** **Household surveys**  **906 household survey responses** on treatment-seeking for severe febrile illness will be required per country and individual survey round allowing for a detection of an increase to 20% with 80% power and α = 0.05 (under the assumption that treatment-seeking at baseline is as low as 15%) |
| **Study Duration:** | Q2 2018 – Q3 2020 |
| **Study Schedule:** | Data collection baseline: Q2 2018 – Q3 2018  Data collection post-implementation: Q1 2019 – Q2 2020  Interim data analysis: Q3 – Q4 2018  Identify second round interventions necessary in each implementation area: Q4 2019  Final data analysis: Q3 2019 – Q2 2020 |
| **Study Sites:** | - Three health zones in DRC (Kenge, Kingandu and Ipamu), - A subset of local government areas (LGAs) in one state in Nigeria (Adamawa) , and - Three districts in Uganda (Apac, Kole and Oyam) |
| **Ethics Statement:** | The research project will be carried out in accordance with the research plan outlined in this protocol and with principles enunciated in the current version of the Declaration of Helsinki, International Conference on Harmonisation ICH Topic E6: ‘Guideline for Good Clinical Practice’. 1996 (CPMP/ICH/135/95), Essentials of Good Epidemiological Practice issued by Public Health Switzerland (EGEP) as well as all national legal and regulatory requirements as applicable. |
| **Study Flowchart with Timelines:**   \|  \| **2017** \| **2018** \| \| \| \| **2019** \| \| \| \| **2020** \| \| \| \| \| --- \| --- \| --- \| --- \| --- \| --- \| --- \| --- \| --- \| --- \| --- \| --- \| --- \| --- \| \|  \| **Q4** \| **Q1** \| **Q2** \| **Q3** \| **Q4** \| **Q1** \| **Q2** \| **Q3** \| **Q4** \| **Q1** \| **Q2** \| **Q3** \| **Q4** \| \| **Project phase** \| **Pre-RAS** \| \| \| \| **R/O** \| **Post-RAS introduction** \| \| \| \| \| \| \| \| \| IEC submission \| X \|  \|  \|  \|  \|  \|  \|  \|  \|  \|  \|  \|  \| \| IEC approval \|  \| X \|  \|  \|  \|  \|  \|  \|  \|  \|  \|  \|  \| \| **Baseline data collection** \|  \|  \| **6 months** \| \|  \|  \|  \|  \|  \|  \|  \|  \|  \| \| PSS \|  \|  \| X \| X \| (X) \|  \|  \|  \|  \|  \|  \|  \|  \| \| HCPS \|  \|  \| X \| X \|  \|  \|  \|  \|  \|  \|  \|  \|  \| \| HHS \|  \|  \| X \| X \|  \|  \|  \|  \|  \|  \|  \|  \|  \| \| EES \|  \|  \| X \| X \|  \|  \|  \|  \|  \|  \|  \|  \|  \| \| Routine data collection \|  \|  \| X \| X \| X \|  \|  \|  \|  \|  \|  \|  \|  \| \| Baseline data analysis \|  \|  \|  \| X \| X \|  \|  \|  \|  \|  \|  \|  \|  \| \| **Post-RAS data collection** \|  \|  \|  \|  \|  \| **18 months** \| \| \| \| \| \|  \|  \| \| PSS \|  \|  \|  \|  \|  \| X \| X \| X \| X \| X \| X \|  \|  \| \| HCPS \|  \|  \|  \|  \|  \|  \| X \| X \|  \|  \| X \|  \|  \| \| HHS \|  \|  \|  \|  \|  \|  \| X \| X \|  \|  \| X \|  \|  \| \| EES \|  \|  \|  \|  \|  \| X \| X \| X \| X \| X \| X \|  \|  \| \| Routine data collection \|  \|  \|  \|  \|  \| X \| X \| X \| X \| X \| X \|  \|  \| \| Post-RAS data analysis \|  \|  \|  \|  \|  \|  \|  \| X \| X \| X \| X \|  \|  \| \| Publication and technical manuscripts \|  \|  \|  \| X \| X \|  \|  \| X \| X \|  \| X \| X \|  \| \| Data Quality Audits \|  \| X \|  \|  \|  \| X \|  \|  \|  \| X \|  \|  \|  \|   IEC = Independent Ethics Committee; PSS = Patient surveillance system (Activity 1); HCPS = Health care provider survey (Activity 2); HHS = Household survey (Activity 3); EES = Economic evaluation study (Activity 4) | |
| **The most relevant References:**  1. WHO, *World Malaria Report*. 2016, World Health Organization.  2. WHO, *Guidelines for the treatment of malaria*. 2015, World Health Organization.  3. Gomes, M.F., et al., *Pre-referral rectal artesunate to prevent death and disability in severe malaria: a placebo-controlled trial.* Lancet, 2009. **373**(9663): p. 557-66.  4. Gomes, M., et al., *Rectal artemisinins for malaria: a review of efficacy and safety from individual patient data in clinical studies.* BMC Infect Dis, 2008. **8**: p. 39.  5. Tozan, Y., et al., *Prereferral rectal artesunate for treatment of severe childhood malaria: a cost-effectiveness analysis.* Lancet, 2010. **376**(9756): p. 1910-5.  6. Habicht, J.P., C.G. Victora, and J.P. Vaughan, *Evaluation designs for adequacy, plausibility and probability of public health programme performance and impact.* Int J Epidemiol, 1999. **28**(1): p. 10-18. | |
|  | |
|  | |

# Background information

## Malaria

Malaria is one of the leading causes of illness, death, and lost economic productivity globally. While the successful scale-up and use of critical commodities such as insecticide-treated nets (ITNs) and artemisinin-based combination therapies (ACTs) have resulted in a 62% decline in malaria-related mortality between 2000 and 2015, malaria still results in over 400,000 deaths each year, most of which are in children under 5 years of age and pregnant women [1]. The heaviest malaria burden continues to be borne by sub-Saharan African countries which accounted for an estimated 90% of malaria cases and 92% of malaria deaths in 2015 [1].

Malaria mortality is a result of the progression of untreated or sub-optimally treated malaria to severe disease. Severe malaria almost invariably leads to death or irreversible sequelae if not appropriately treated. With prompt, effective antimalarial treatment and supportive care, severe malaria mortality rates can be substantially reduced [2]. The risk of death is greatest in the first 24 hours of onset of illness [7] and timely provision of adequate treatment and care is therefore paramount.

Many of the remaining malaria deaths happen in remote settings with poor access to formal health facilities. As a result, many patients do not receive the necessary treatment and care in a timely fashion. In a study conducted with over 6,000 children seeking treatment for suspected severe malaria in Ghana and Tanzania, of the children who survived the first six hours after seeing a health worker at the community level, approximately 45% were still not at a referral health facility after more than six hours. Of those children, about half were still not there after more than 15 hours [3]. Without an effective pre-referral treatment that can be administered at the community level, many of these cases will result in death.

## Treatment of malaria

Oral artemisinin-based combination therapy (ACT) is currently the most effective and recommended treatment for episodes of uncomplicated malaria [2]. Treatment should be based on the parasitological confirmation of infection by light microscopy or malaria Rapid Diagnostic Test (mRDT).

Patients with severe malaria on the other hand (including infants, pregnant women in all trimesters and lactating women) should first be treated with intravenous or intramuscular artesunate for at least 24 hours until the patient can tolerate oral medication. Parenteral artemether and quinine are alternatives where injectable artesunate is unavailable. Once a patient has received at least 24 h of parenteral therapy and can tolerate oral therapy, a full 3-day treatment course of ACT must be administered [2]. Parenteral artesunate has been shown to reduce mortality in both children and adults as compared to parenteral quinine [8, 9]. Comprehensive supportive treatment and intensive care may be required depending on the severity of the illness.

## Rectal artesunate as pre-referral treatment

A full treatment course for a severe malaria episode inclusive of parenteral and oral therapy is often not accessible in time due to frequent long delays in referring patients to an appropriate health facility [3]. Yet, without effective pre-referral treatment that can be administered at the community level, many cases of severe malaria will result in death. In such situations, where parenteral treatment of severe malaria is not available, rectal artesunate (RAS) can be an effective pre-referral treatment for young children^[[1]](#footnote-2)^. It rapidly (i.e. within 24 h) clears 90% [4] or more of malaria parasites, and in children less than 6 years old who cannot reach a facility in less than six hours, it can reduce the risk of death or permanent disability by up to 50% [4].The World Health Organization (WHO) therefore recommends treating children less than 6 years old initially with a single rectal dose of 10 mg artesunate per kilogram of body weight [2].

After the administration of rectal artesunate (RAS), the child should be referred immediately to an appropriate facility where the full package of care for severe malaria can be provided. WHO considers the administration of RAS as pre-referral treatment feasible and acceptable even at community level [2].

As a result of the Improving Severe Malaria Outcomes (ISMO) project funded by Unitaid and implemented by Medicines for Malaria Venture (MMV), two new RAS products (developed by the Indian pharmaceutical companies Strides and Cipla) were submitted for WHO prequalification (PQ) in Q1 2016. This has opened the way for large-scale implementation in the near future. Cipla received Global Fund Expert Review Panel (ERP) approval in December 2016. In 2017, 100 mg RAS has been added to the WHO Model List of Essential Medicines (EML) and Model List of Essential Medicines for Children (EMLc).

## Project justification

Appropriate management of children with signs of severe malaria at the periphery of health services and their referral to higher level health facilities, where severe disease can be managed, is vital in the fight to reduce malaria morbidity and mortality (WHO Global Technical Strategy for Malaria 2016-2030, Goal 1: Reduce malaria mortality rates globally compared with 2015; by at least 40% by 2020; at least 75% by 2025; at least 90% by 2030 [10]).

The World Health Organization recommends treating children less than 6 years of age with artesunate suppositories in situations where parenteral artesunate is not available [2]. While this recommendation by the WHO is strong, it is based on evidence from only one study that the WHO considers of moderate quality and comes with limited operational guidance [4]. There are currently no evaluations of using RAS at the level of community based health care providers under real-world operational circumstances.

Currently, 16 countries in Africa include the use of pre-referral RAS in their treatment policies, but guidelines for use vary widely across these countries and often they do not align with the WHO recommendation. In some countries, RAS is recommended for use in adults, although this is not recommended by the WHO as it may lead to adverse effects [4]. The only study that formally evaluated the use of pre-referral RAS in Bangladesh, Ghana and Tanzania found a reduction in mortality for children under 6 years, but also an indication that in Asia use of the product might increase mortality for children older than 6 years [3]. Despite any such misalignment and lack of operational guidance, countries have begun to procure and use RAS. Widespread use of this product without alignment with WHO recommendations raises substantial concerns, including regarding its inappropriate use as a monotherapy against both severe and uncomplicated malaria.

With the impending availability of quality-assured rectal artesunate (QA RAS) and countries poised to scale-up this intervention, it is now a critical time to investigate the safe and effective implementation of RAS in existing community-based case management systems, as part of a continuum of care for severe malaria patients. The pilot roll-out of QA RAS in the Democratic Republic of the Congo (DRC), Nigeria and Uganda provides a unique and timely opportunity to generate high-quality evidence applicable to a wide range of African settings.

## Roll-out of rectal artesunate in DR Congo, Nigeria and Uganda

In the three countries DRC, Nigeria and Uganda, RAS is part of the countries’ current national malaria treatment policy. In parts of these three countries, QA RAS will be rolled out in the near future at community level through integrated Community Case Management (iCCM) schemes, with support from various donor agencies.

Unitaid will support a pilot roll-out of QA RAS by UNICEF and its partners in selected areas of the three countries linked to the research project outlined in this protocol. In the context of the Unitaid-supported pilot roll-out, QA RAS will be procured through UNICEF as suppositories containing 100 mg of artesunate, and implemented through existing iCCM schemes. In addition to meeting the WHO PQ quality criteria, the product will require regulatory registration / import licencing or a specific waiver in each of the RAS project countries before delivery can be initiated. Product characteristics and dosage information is provided for reference purposes in Appendix I.

The pilot roll-out will be accompanied by the multi-country observational research studies described in this protocol. It is to our best knowledge the first and only current study to investigate key questions linked to the large-scale roll-out of pre-referral RAS.

# Overall goal, objectives and outcomes

## Overall goal

The overall goal of the “**C**ommunity **A**ccess to **R**ectal **A**rtesunate for **Mal**aria” (CARAMAL) project is to contribute to reducing malaria mortality in children globally by improving the community management of suspected severe malaria cases.

The CARAMAL project will contribute to this goal by advancing the development of operational guidance to catalyse effective and appropriate scale-up of QA RAS as pre-referral treatment of severe malaria. Results from this study will guide the design of appropriate evidence-based strategies for implementation and scale-up of RAS in the project areas and beyond.

## Study objectives and research questions

### Objectives

Accompanying the pilot roll-out of QA RAS by UNICEF, the CARAMAL project will test whether it is feasible to introduce QA RAS into established integrated community case management (iCCM) platforms with only minimal additional supportive interventions and with minimal unintended consequences such as inappropriate use as artemisinin monotherapy.

For this purpose, the CARAMAL project will implement a set of complementary research studies to provide evidence on the impact and practical operational and health systems-related factors relevant for the introduction of community-based pre-referral treatment with QA RAS. The evidence generated by the CARAMAL Project will be used in the development of evidence-based operational guidance to catalyse scale-up of pre-referral QA RAS.

### Research questions

Through the research activities described in detail below, the CARAMAL project aims to answer the following research questions:

1. What are the **minimal requirements** of a community case management system **to ensure that RAS is an effective part of the continuum of care** from the community to a referral facility (defined as a health care facility equipped for inpatient care of severe malaria)?
   1. *What are obstacles (including operational aspects and acceptability at all levels) to the appropriate implementation of the RAS guidelines?*
   2. *What realistic approaches/measures are needed to support the appropriate implementation of pre-referral rectal artesunate by health workers?*
2. What are the **unintended consequences** of scaled implementation, such as adverse drug reactions, unforeseen costs [5], or unforeseen issues in treatment of malaria at all levels of care, and how can they be addressed?
   1. *How does treatment seeking for mild and severe malaria episodes change following the introduction of RAS?*
3. Is there any **use of RAS beyond the recommended guidelines**, including full treatment of severe cases with RAS at community level, and the treatment of uncomplicated malaria with RAS?
   1. *Does the introduction of RAS promote the use of a monotherapy treatment against uncomplicated falciparum malaria and if so, what interventions are necessary to avoid this inappropriate use?*
4. Can the introduction of pre-referral QA RAS **reduce severe malaria case fatality ratio** over time under real-world operational circumstances in three distinct settings?
   1. *How does malaria case management (including referrals and case management at referral facilities) change following the introduction of RAS?*
   2. *How successful are referrals for severely ill children, both in terms of completion of referral and reduction in case fatality ratio?*
5. What are the **costs and cost-effectiveness** of community and peripheral health facility based RAS?

## Study outcomes

The principal indicators that will be assessed in the context of this project are presented in Table 1. The data sources mentioned in the table are described in detail in the subsequent chapters.

Table 1. Key indicators assessed by the CARAMAL Project – with related activities

| **Research question(s)** | **Indicator** | **Source** | **Indicator description** | **Frequency** |
| --- | --- | --- | --- | --- |
| **I** | QA RAS coverage | M&E, HCPS | Proportion of trained and functional CHW and primary HF who provide QA RAS | Annually |
|  | RAS availability | M&E, HCPS | Proportion of CHW/primary HF with RAS in stock | Annually |
|  | Functional referral facilities | HCPS | Proportion of referral health facilities that have the capacity to manage severe malaria in children in line with global guidance | Annually |
|  | Supervision | M&E, HCPS | Proportion of CHW/primary HF that received at least one supervisory visit in the past 3 months | Annually |
| **I + II** | Adverse events | PSS | Frequency of passively reported adverse events after RAS administration | Continuous |
|  | Delayed haemolytic anaemia | PSS | Frequency of delayed haemolytic anaemia within 28 days after RAS administration | Continuous |
| **II i** | Treatment seeking | HHS | Proportion of children <5 years with a recent history of fever (mild or severe) who attend CHW/primary HF | Annually |
|  | CHW attendance | PSS | Proportion of children <5 years with a recent history of fever (mild or severe) who attend CHW/primary HF | Annually |
|  | RAS acceptability among health workers | HCPS | Acceptability of pre-referral RAS among health workers | Annually |
|  | RAS acceptability in community | HHS | Acceptability of pre-referral RAS among caretakers | Annually |
|  | Referral | PSS | Proportion of children <5 years with severe febrile illness seen by CHW/primary HF who completed referral | Continuous |
|  | Direct reporting | PSS | Number of children <5 years with severe febrile illness who report directly to RHF | Continuous |
| **I + III** | Pre-referral treatment | PSS | Proportion of children <5 years with severe febrile illness managed pre-referral according to guidelines | Continuous |
|  | Post-referral treatment | PSS | Proportion of children <5 years with severe febrile illness managed post-referral according to guidelines | Continuous |
| **IV** | Case fatality ratio | PSS | Proportion of children <5 years with severe febrile illness seen by CHW/primary HF that resulted in death within 28 days | Continuous |
|  | Malaria infection on day 28 | PSS | Proportion of children <5 years with severe febrile illness without parasites on day 28 | Continuous |
| **IV ii** | Successful referral | PSS | Proportion of children <5 years with severe febrile illness seen by CHW/primary HF who completed referral to a referral health facility | Continuous |
| **V** | Costs | EES | Total financial cost of managing severe case at community level.  Marginal financial cost of adding RAS to the management. | Continuous  From project records |
|  | Cost-effectiveness | PSS EES | Financial cost of RAS interventions per death averted. | PSS and project records |
| **Exploratory / contextual** | Danger signs | HHS | Proportion of children <5 years with a recent history of fever who reported danger signs (signs of 'severe febrile illness'). | Annually |
|  | Malaria prevalence | HHS | Proportion of children <5 years infected with malaria parasites | Annually |

PSS = Patient surveillance system (Activity 1); HCPS = Health care provider survey (Activity 2); HHS = Household survey (Activity 3); EES = Economic evaluation study (Activity 4); HF = Health facility; M&E = routine M & E activity (Activity 5)

## Monitoring of Artemisinin resistance

In the context of the CARAMAL Project, children treated with a single dose of QA RAS at community level (CHW or primary health facility) should subsequently receive parenteral treatment with artesunate, followed by a full course of an ACT at a referral facility, according to national guidelines. While resistance of the *Plasmodium* parasites against artemisinin has not yet been document in the study settings, this is obviously a great concern to public health practitioners and researchers. Of special concern in the frame of the present project is the fact that RAS is a monotherapy, and that the widespread administration of artemisinin monotherapies could increase drug pressure and subsequently lead to the selection of drug resistance conferring mutations. The CARAMAL project will be addressing these concerns in two ways.

Firstly, the CARAMAL project will be directly monitoring the frequency of inappropriate use of QA RAS, including its use as a monotherapy and inadequate subsequent treatment including failure to administer a full course of ACT once oral treatment can be tolerated. On the basis of these findings, appropriate mitigation measures (like additional training and supervision, drug provision etc.) will be discussed with health authorities and iCCM managers, and then implemented as soon as possible.

Secondly, a complementary study linked to the CARAMAL activities aims to monitor the frequency of resistance markers in the study settings. This study will be carried out in close collaboration with the Global Malaria Programme of the WHO under a separate complementary protocol. Specifically, the project will continuously sample children with malaria in the three study settings in order to assess over time the prevalence of molecular markers of artemisinin resistance (K13-propeller sequence polymorphisms). Ultimately, that study aims to monitor whether the introduction of RAS could increase the selection of resistant *Plasmodium* strains. The testing for these markers will be carried out in a WHO-selected laboratory. The details of sampling and testing will be described in a separate study protocol, to be submitted separately for ethical review and approval. Sample collection, once approved, will be linked to the Patient Surveillance System described in Chapter 4 of the present protocol.

# Study sites

The CARAMAL project will be implemented in three sub-Saharan African countries with an ongoing high burden of malaria. The three project countries, the Democratic Republic of the Congo (DRC), Nigeria, and Uganda together share an estimated 42% of total global malaria cases and 39% of total global deaths (Figure 1 and Table 2). The scale up of QA RAS in these three countries would thus have a significant impact on global malaria mortality.


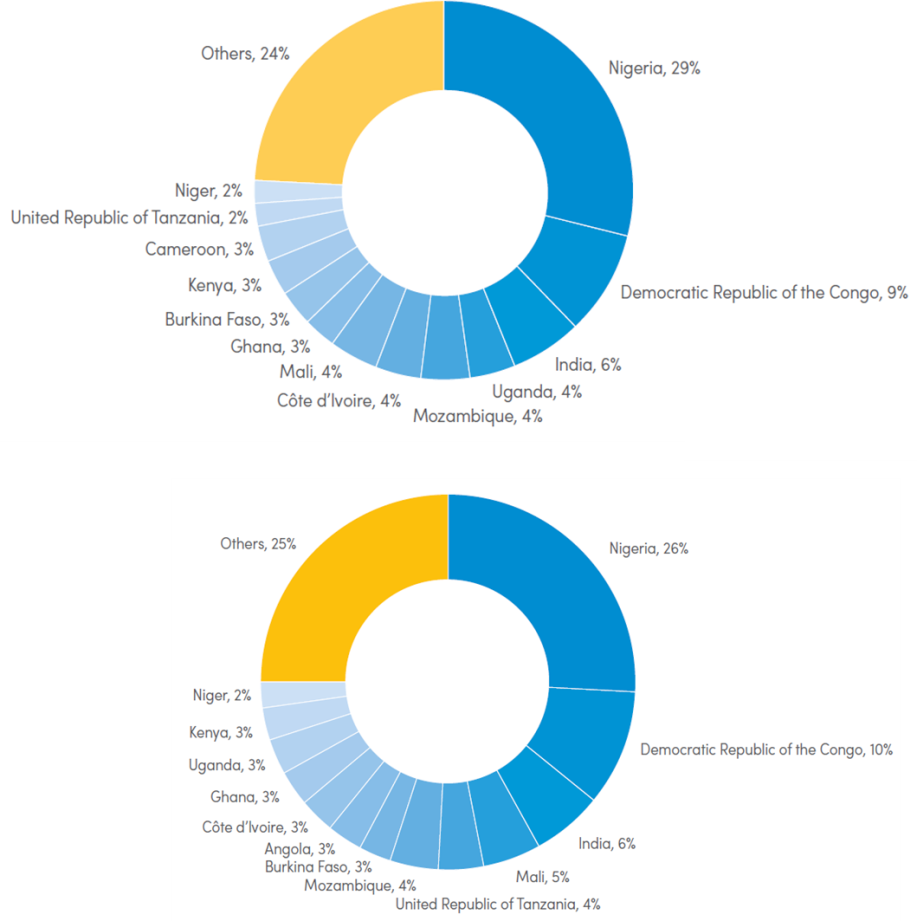


Figure 1. WHO estimated country shares of total malaria cases (top) and total malaria deaths (bottom) [1]

Table 2. Malaria burden and mortality in the three study countries [1]

|  | **DRC** | **Nigeria** | **Uganda** | **Total** |
| --- | --- | --- | --- | --- |
| Estimated malaria cases | 19 Mio | 61 Mio | 8,5 Mio | 88,5 Mio |
| Estimated malaria deaths | 42,000 | 110,000 | 12,000 | 164,000 |

Additional criteria to select these countries were geographical and cultural diversity, existence of functioning local iCCM programs, commitment of the National Malaria Control Programs, and presence of CARAMAL Project Consortium members. The selected countries represent a geographical mix with one country from each region of West, Central and East Africa. The inclusion of DRC ensures francophone representation and applicability of the project to other francophone African countries.

## Integrated community case management and RAS

Integrated Community Case Management (iCCM) is a strategy to provide populations, especially children under five years, with limited access to facility-based health care services with timely and effective treatment for malaria, pneumonia and diarrhoea. Community health workers (CHW, depending on the local context also called village health teams (VHT), or community oriented resource persons (CORP)) are appropriately trained low-level health personnel, supervised and supported with an uninterrupted supply of medicines and equipment. It has been shown that they can identify and correctly treat many sick children using oral rehydration salts (ORS), zinc, oral antibiotics, and artemisinin based combination therapy (ACT) [11]. In addition, the availability of high-quality rapid diagnostic tests for malaria (mRDTs) has made it possible for them to test for malaria at the community level. But clearly, CHW cannot manage more severely ill children, and there is therefore need of a functioning referral system.

The three selected countries have ongoing funding for iCCM programmes that can be leveraged to implement RAS. UNICEF has been supporting the iCCM programmes through supervision, training and provision of commodities. In the local health system context, the iCCM programmes are implemented through community health worker (CHW) schemes which are named differently in each country. In DRC, iCCM is implemented in remote populations through Community Care Sites (*sites de soins communautaires*) operated by volunteer community health workers (*relais communautaires*). In Nigeria, the volunteer health workers providing iCCM services are called Community Oriented Resource Persons (CORP) and in Uganda, iCCM is rolled out through the Village Health Team (VHT) system. In this protocol, community-based providers in all three countries are collectively called “CHW”.

There is Global Fund funding for iCCM programs to cover malaria commodities such as ACTs and mRDTs, along with supportive interventions, such as training. While in DRC and Uganda, the CHWs are not paid or paid only a minimal financial incentive, in Nigeria they are paid on a monthly basis. So the selected countries cover both models.

Though it is assumed that CHWs and peripheral health facility workers in the study areas are already trained in malaria case management, it is important that these CHWs and peripheral health facility workers are trained on how to properly administer and when to use RAS so as not to promote overuse of RAS in uncomplicated malaria. This training will be implemented by UNICEF before RAS roll-out and will include modules on identifying malaria danger signs (suspected severe malaria), administering pre-referral QA RAS, referring patients, patient follow-up, and using referral slips and reporting templates as well as further modules informed by the issues observed during the baseline assessment of this study. iCCM community communication and mobilization strategies will incorporate information about QA RAS availability for pre-referral treatment of suspected severe malaria and about the importance of immediate and completed referral if QA RAS is administered.

All three selected countries have RAS included in their national treatment policy. The National Malaria Control Programmes in each of the three countries have confirmed their commitment to pre-referral QA RAS, and have included the implementation of QA RAS at community and peripheral health facility levels in their National Strategic Plans and in the National Treatment Guidelines.

## Selection of study area within each country

The selection of the study areas within each country has been guided by several scientific and operational considerations (8.2 Determination of sample size), including the population required for meeting the minimum sample size (described in more detail in the respective chapter below), a functioning iCCM programme supported by the Consortium Partner UNICEF, a functioning referral system, and an acceptable security situation. Based on these considerations, the following project areas were selected:

- three health zones in DRC (Kenge, Kingandu and Ipamu),
- three districts in Uganda (Apac, Kole and Oyam), and
- a subset of local government areas (LGAs) in one state in Nigeria (Adamawa)

In preparation of the project, an abridged health facility survey of the referral health facilities within the project area was conducted to ensure that a minimal set of conditions are met, ensuring adequate post-referral treatment and care for cases of severe malaria / severe febrile illness. This assessment included an evaluation of the existence of an emergency triage system, availability of life-saving post-referral treatment, malaria diagnostic tools (mRDT or light microscopy with relevant reagents), adequate training of health workers, operational in-patient wards and functional patient record management systems. And any necessary minimal supportive interventions revealed by this assessment will take place before (and, if necessary, alongside) the roll-out of QA RAS and accompanying behaviour change messages at the community level.

The total combined catchment population of the CARAMAL project area comprises approximately 2.8 million people (Table 3). The study population for the various research activities in this project includes children, adults, and health professionals in the project areas. In the case of severe febrile illness patients, this study will focus on the age group < 5 years, which is in accordance with the age group established in the iCCM algorithms (while the WHO treatment guidelines recommend the administration of RAS to patients < 6 years). The specific study populations are defined in the following chapter for each individual research activity.

Country-specific community engagement plans will be developed by the implementing Consortium Partner UNICEF, containing messages on the appropriate treatment of severe malaria, highlighting the intended use of RAS strictly as a pre-referral treatment that requires subsequent complementation by a full course of anti-malarial treatment and adequate care by medical professionals.

Table 3. Study areas and study populations in the three Project countries

|  | **DRC** | **Nigeria** | **Uganda** | **Total** |
| --- | --- | --- | --- | --- |
| **Project area** | Kenge, Kingandu and Ipamu Health Zones | Adamawa State, selected LGAs | Apac, Kole and Oyam Districts |  |
| **Population** | 619,000 | 1,224,000 | 992,000 | 2,835,000 |
| **Population <5 years** | 112,000 | 245,000 | 176,000 | 533,000 |

🡺 Detailed profiles of the project countries and study areas are provided in Appendix II.

# Project design and data collection procedures

The CARAMAL project has been designed as a multi-country operational research study implemented in three highly malaria-endemic countries. It will be based on a before-and-after plausibility design [6] aligned with the roll-out of QA RAS through established community-based health care provider systems.

Data collection will span six months prior to the roll-out of QA RAS (baseline) and at least 18 months thereafter. As previously described, QA RAS will be implemented independently through established in-country collaborative arrangements and following existing national guidelines. Depending on the project country and the local preferences, RAS may be implemented through CHW only, or through CHW and primary health facilities.

The CARAMAL project will be implemented by a consortium comprised of the Clinton Health Access Initiative (CHAI), UNICEF, the Swiss Tropical and Public Health Institute (Swiss TPH) and Country Research Partners:

- **CHAI** as the lead grantee of the RAS implementation project is responsible for the overall governance of the project.
- **UNICEF** will procure QA RAS and support its introduction into functional local iCCM systems according to existing guidelines, ensuring that all personnel within the continuum of care are prepared and supported to adequately administer QA RAS. Performance will continuously be subject to M&E activities which feed into the research activities.
- **Swiss TPH** is responsible for generating and disseminating scientific evidence as described in detail in this protocol.
- **Country Research Partners** will be responsible for the local implementation of all research (non-routine) data collection and country-level analyses:
  - DRC: **Kinshasa School of Public Health, University of Kinshasa**
  - Nigeria: **Akena Associates Ltd.**
  - Uganda: **School of Public Health, Makerere University**

The CARAMAL project has been presented to and was accepted by the national and local health authorities in all three implementation countries. They have expressed their commitment to supporting implementation of this project but will remain independent from scientific evaluation of study outcomes pertaining to the health system. However, a regular information exchange between health authorities and the project consortium will ensure alignment of research activities with national practices and guidelines and translation of study findings into operational policy.

The research activities will consist of several interlinked and complementary observational studies applying a range of methodological approaches. They can be presented as five major activities:

**Activity 1:** A **patient surveillance** **system** to assess severe febrile illness/suspected severe malaria incidence, case fatality rate, and related clinical patterns, diagnoses, treatment and treatment outcomes from first contact to the point of recovery or death (baseline and after RAS roll-out)

**Activity 2:** **Health care provider surveys** to establish the availability and uptake of QA RAS at all levels, and health providers’ knowledge, attitudes and practices towards RAS (baseline and after RAS roll-out)

**Activity 3:** **Household surveys** to assess treatment seeking, caretakers’ knowledge and attitudes towards RAS, and malaria intervention coverage at community level (baseline and after RAS roll-out)

**Activity 4:** An **economic evaluation** to assess the costs and incremental cost-effectiveness of implementing RAS at community level compared to the current standard of care.

**Activity 5:** **Routine monitoring of process indicators** **along the entire case management chain** to continuously assess implementation progress of RAS as contextual information for other outcome indicators

Each activity is described in detail below. Research activities will be implemented in each country by the respective Country Research Partner in collaboration with Swiss TPH.

## Activity 1: Patient surveillance system

A patient surveillance system (PSS) will be established in the project sites allowing the continuous registration and follow-up of all children <5 years of age who present to a CHW or primary health care facility with severe febrile illness and are referred from there to a higher-level facility.

The PSS will track patients at the following three points of contact (Figure 2):

1) **CHW and/or primary health facility^[[2]](#footnote-3)^:** first contact and registration into the PSS

2) **Referral health facility:** second contact for those patients who successfully completed referral and first contact for patients directly attending a referral facility

3) **Home, 28 days after first contact:** last contact for all registered patients (in case of death of a child, this visit will be postponed to 8 weeks after the day of death)

At each point of contact, a distinct set of data will be collected using a combination of established routine records and study-specific data collection procedures. Data collection will be implemented at each point of contact independently. Individual patient data from each point of contact can be linked based on a unique study ID assigned to each patient at the first point of contact and recorded at each subsequent point of contact. Study IDs will be composed as follows: [country code]-[provider code]-[serial number 0 to n].

Data collection at the first two points of contact will capture the health status of the patient and the diagnostic tests, treatment and referral provided, while at the last point of contact, the health status and, retrospectively, treatment seeking will be assessed. The retrospective treatment seeking assessment on the last day will serve to triangulate and complement data collected at first and second points of contact as well as fill information gaps for those patients who did not successfully complete referral to a formal referral health facility.

Data collected from this activity will inform the following outcome indicators and changes in these indicators over time:

- Suspected severe malaria case fatality rate
- Access to and rational use of QA RAS as part of a continuum of care
- Unintended consequences of QA RAS introduction
- Patterns of illness
- Case management and referral
- Affordability
- Treatment outcome
- Passive reporting of adverse events

### Study population

The PSS will be implemented through all CHW / primary health facilities and all major referral facilities operating in the project areas.

The PSS will include all children <5 years of age seeking care for a current/recent febrile illness episode at the level of community based care providers in the study areas, including CHWs and primary health facilities. All children diagnosed at that level with severe febrile illness / suspected severe malaria will then be enrolled in the PSS and tracked at the referral health facility and at the child’s home 28 days after initial diagnosis.

In addition, all children <5 years of age seeking care for a severe febrile illness episode directly at the referral facility will be enrolled to evaluate treatment seeking, diagnosis, treatment and disease outcome. These children will be excluded from the case fatality ratio analysis.

During baseline (i.e. pre-RAS roll-out), RAS is expected not to be available at community level in the study area, and the PSS will enrol primarily children not treated with pre-referral RAS, whereas after RAS roll-out, RAS is expected to be administered at the level of community based health care providers. Due to the nature of the observational study design, the treatment with RAS will be a function of the CHW/health workers’ and patients’ adherence, which will be assessed as an outcome and explanatory variable for the impact assessment. Enrolment of children will be done by CHW/health worker making the initial assessment of the child (and after RAS roll-out, pre-treating the child with RAS). During their visit to health care providers and the post-treatment home visits, parents/caretakers of the children will be asked to participate in an interview.

### Procedures and instruments

Data will be collected from eligible patients at each of three potential points of contact, the CHW/primary health facility, the referral facility treating cases of suspected severe malaria, and at home (Figure 2).


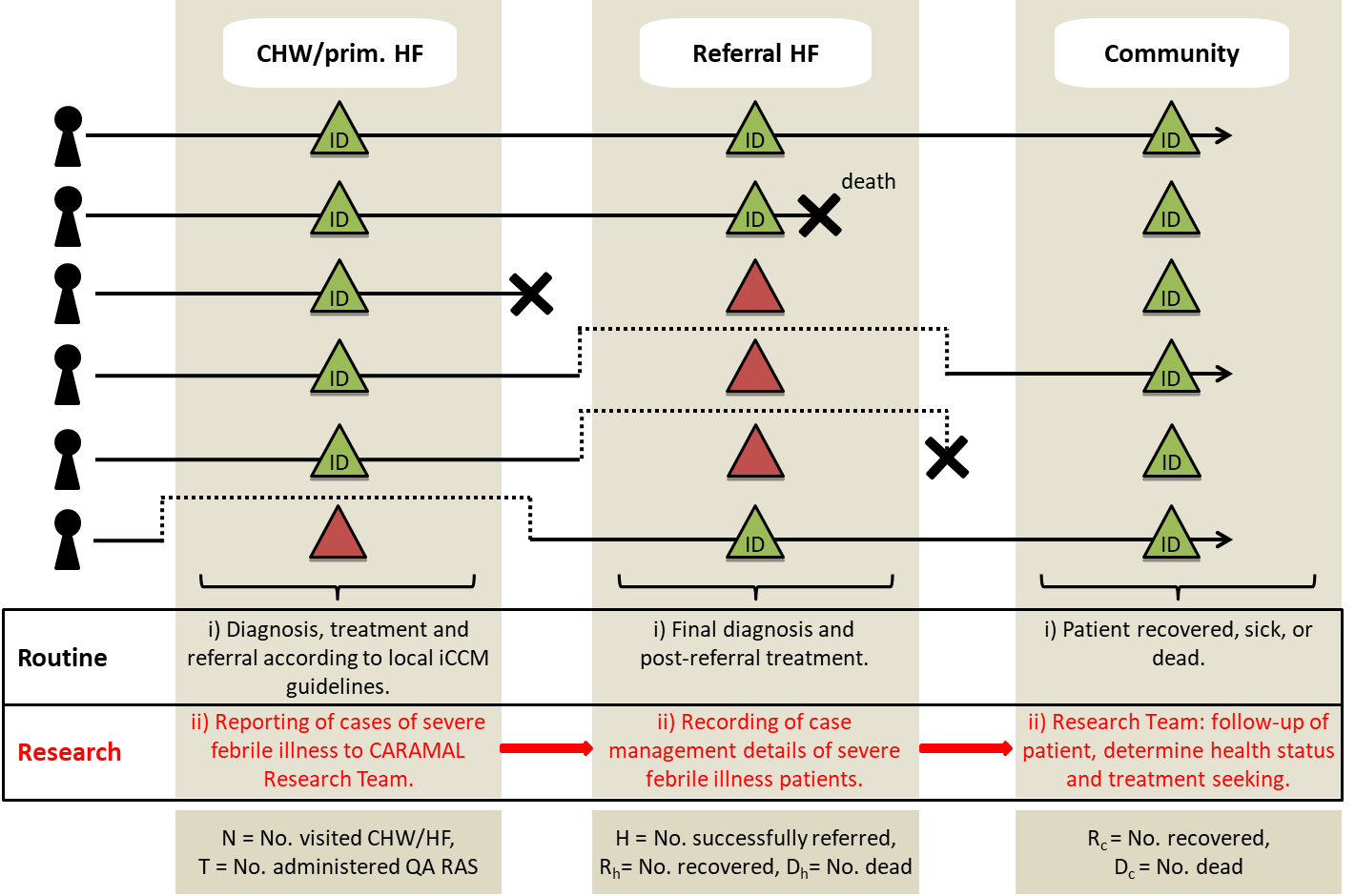


Figure 2. Schematic representation of possible points of contact in the Patient Surveillance System. ID = identification by unique study ID. Green triangles represent points of contact, red triangles no contact.

At each point, a distinct yet overlapping set of data will be collected, as described below. Patients enrolled into the PSS by the community-based health care providers will be identified at each point of contact through a unique study ID assigned at the time of enrollment.

**1) CHW / primary health facility (first contact)**

According to established routine practice, the following procedures are performed^[[3]](#footnote-4)^: children attending a CHW or primary health facility undergo examination, treatment and referral procedures as per local guidelines. An mRDT is performed on all children with a history of or acute fever, including children with symptoms of severe febrile illness / children with danger signs (see “Biological sample collection and diagnostic tests”, page 44). The children are then treated as per the applicable national guidelines. Performing an mRDT on all fever cases regardless of their severity is part of the current practice in the three countries. It is also crucial for study purposes in order to obtain a malaria diagnosis from all severe febrile illness patients irrespective of the completion of the subsequent referral and to allow establishing a denominator for malaria-relevant events. Following established iCCM protocols, children < 5 years of age with severe febrile illness are referred to a higher level facility for appropriate treatment and care. After baseline and following the roll-out of QA RAS and supportive measures, these patients will be administered QA RAS prior to their referral. Severely ill patients who are referred are issued a Referral Form, which is part of recommended routine practice in all three countries. The form specifies (at a minimum) the patient’s details, date and time of the visit, the reasons for referral, and the name of the referral facility.

The patient’s details, diagnosis, danger signs, treatment and referral information are also recorded routinely in the CHW or primary health facility’s paper record books. Aggregate categorized case numbers will be reported regularly, but depending on the country setting through different means, to supervisors and/or the national health information system.

The research team will extract data from the CHW and primary health facility’s case registers and/or individual patient files on a regular basis, with the schedule adapted to each country’s individual situation, e.g. during monthly or quarterly CHW-supervisor meetings or during personal visits to the CHW. The forms collect details on present signs and symptoms (including danger signs), RAS / ACT dosing, any adverse reaction, referral, and any follow-up action taken by the CHW. The details of the content of these forms depend on the local implementation of the iCCM guidelines. The data will then be copied from these forms into an electronic database using tablet computers and ODK software.

Children who were diagnosed with severe febrile illness and requiring referral following the local iCCM algorithm are eligible for enrolment into the PSS.

In addition to routine practice, the following study-specific procedures will then be performed for these patients by the CHW / primary health facility staff:

1. Preliminary oral informed consent will be obtained by the CHW or a dedicated health facility staff member from parents/caretakers prior to registration in the PSS. The consent will cover two aspects, namely, the performance of an mRDT (only if not routinely done by the CHW) and the assignment of a study ID and registration of the patient in the Central Patient Register for later follow-up by the research team, either at the referral facility, or at the patient’s home. This preliminary informed consent will be complemented with a full written consent as described below.
2. A unique study ID will be assigned to each child for which preliminary informed consent was provided. The unique ID will be issued as pre-printed serial number on stickers that will be affixed to the Referral Form provided to the parent/caretaker. The project will support (and regularly monitor) the routine use of Referral Forms as per local guidelines. These forms carrying the unique study ID will serve to identify the patient at the referral health facility.
3. The unique study ID will be recorded in the CHW/primary health facility’s case register, next to the patient’s details.
4. On a weekly basis, CHW/primary health facilities who submit aggregate case reports of at least one severe febrile illness case that was subsequently referred will be contacted by the research team (e.g. via a mobile phone based system or oral communication, depending on the local context). The research team will obtain basic details of the patients, including their assigned study ID, name, age, sex, residence and (if possible) contact details from the CHW/primary health facility staff and enter the information into the electronic Central Case Register. These records serve only as an overview of all patients that have been registered by assigning them a unique ID. The records will be accessible to the local research coordinator in order to verify the reports, monitor follow-up at the referral facility, and plan the day 28 household level visit. Aggregated data will be collected during regular routine CHW-supervisor meetings.

*Operational considerations: The minimum sample size of 1,067 severe malaria cases per year (see chapter 8.2 “Determination of sample size”) translates into 89 cases that need to be registered per month or 22 per week, per country.*

Information collected continuously through this activity will allow to

- Calculate rates of treatment with RAS and other routine indicators
- Calculate the frequency of particular danger signs (in countries with individual patient records)
- Monitor rates of enrolment in the PSS
- Verify details of patients enrolled in the PSS

CHW and primary health facility staff will undergo study-specific training led by sub-investigators of country research organizations or delegated staff. The training will consist of lectures on the project background, methodology, research ethics and intensive instruction and practice on completion of study specific procedures, including the informed consent process. The community-based providers will be trained to assign study IDs to patients meeting the specific study enrolment criteria that are indicative of severe febrile illness following local guidelines (i.e. the target group for QA RAS roll-out). The training will also emphasize the need to record the mRDT result on the referral form. They will be expected to emphasize to the parents/caretakers the importance of presenting the referral letter upon arriving at the referral facility (for easy identification by the study nurse based at the referral facility)^[[4]](#footnote-5)^.

**2) Referral facility**

According to established routine practice, the following procedures are performed: Children with severe febrile illness / suspected severe malaria and referred to a referral facility in the study area will be registered upon arrival in the facility. This process, following routine triaging and registration procedures established at the referral facilities, will be strengthened to ensure the immediate identification of severe febrile illness (suspected severe malaria) patients who were either referred by a community-based provider (and registered in the PSS), or who directly attended the referral facility. Patients are then assessed by clinical staff and admitted to an inpatient ward (unless the patient has already improved sufficiently to not require admission). Individual patient data is recorded in specific record books and forms in the facility and usually also in the patient’s health book (details differing per country).

The following study-specific procedures will be performed at referral health facilities:

1. A CARAMAL study nurse will be based at each major referral health facility. The study nurse or a trained local health facility staff will screen incoming patients to identify referrals from a community-based health provider with a referral letter (Figure 3), as well as severe fever patients directly attending the referral facility. For referred patients, the date and time of arrival will be recorded.
2. Full written informed consent will be obtained from parents/caretakers for the provision and use of data previously collected at the level of the community-based provider and for data collected at the referral facility and during the follow-up on day 28. For children directly seeking care at the referral health facility, a full written informed consent will be obtained as soon as the child has received adequate treatment. This procedure will be carried out by the study nurse based at the referral facility. Patient data will only be transferred from facility records into the study database if full informed consent is provided at this stage.
3. For referred and registered patients, the study ID will be recorded alongside their medical records. Patients who directly called in at the referral facility will be assigned a new unique study ID by the study nurse, provided informed consent is given.
4. The study nurse will monitor the case management of each registered patient and continuously enter study-specific details on diagnosis and treatment provided throughout the patient’s admission into an electronic case report form using Open Data Kit (ODK; described in “Description of data management”, page 49). Data collection forms and electronic databases will be pilot-tested and adapted if necessary.
5. Routine and study specific data captured at referral facilities include the patient’s unique study ID, (confirmatory) diagnosis, signs and symptoms of malaria, disease progression, treatment outcome (recovery, death), details on malaria and any other treatment, timing of procedures (time of admission, time of consultation, time of treatment), Hemocue results (Hb), observed adverse events, and treatment outcome (Table 4).

*Operational considerations: 89 cases that need to be registered per month or 22 per week, per country, translate into <5 cases per week per referral facility if 5 referral facilities are staffed with a study nurse, or <3 if 10 facilities are included. It is likely that at least an equal number of severe fever patients will directly call in at the referral facility. As the study nurse does not need to be present constantly, s/he is likely to have some but not excessive time to conduct day 28 follow-up visits (see below).*

Data collected at this level will allow calculating

- the proportion of severe febrile illness patients that completed referral to a designated referral health facility providing severe malaria treatment
- the proportion of severe febrile illness patients being referred by community based providers vs. directly attending a referral facility
- the proportion of severe febrile illness patients with a final diagnosis of malaria
- the proportion of these patients provided treatment according to national guidelines
- the average delay between initial diagnosis and referral by a CHW/primary health facility and arrival at the referral facility
- the case fatality rate among all admitted patients

Study nurses will undergo study-specific training led by co-investigators from country research organizations or delegated staff. The training will consist of lectures on the project background, methodology, research ethics and intensive instruction and practice on completion of study specific procedures, including the written informed consent process. Referral health facility staff involved in the management of severe febrile illness in children will also undergo study-specific information and training sessions in order to ensure data flow and data quality between facility staff, study nurse and research team.

**3) At home (day 28)**

Twenty-eight days after initial treatment by a community-based care provider, all children with severe febrile illness / suspected severe malaria and enrolled into the PSS by a CHW / primary health facility will be followed up at their home by a member of the research team. Likewise, all enrolled children that sought care for a current/recent febrile illness episode directly at the referral facility and enrolled in the PSS will be followed up at their home twenty-eight days after admission to the referral facility. The primary purpose of this visit is to establish the health status of the child (by recording any current signs and symptoms as reported by the parent/caretaker and assessing malaria infection and anaemia). Depending on the country-context, this visit will be carried out by a researcher or by the study nurse based at the nearest referral facility. Wherever possible, the visit will be conducted jointly with the CHW who initially diagnosed the patient in order to strengthen the process of patient follow-up that is an integral part of most iCCM guidelines (Figure 3).

For children that were not referred to a referral facility, a full written informed consent will be obtained from parents/caretakers for the provision and use of data previously collected at the level of the community-based provider and for data collected during the visit on day 28.

The following data will be collected using ODK (see Description of data management”, page 49). Forms will be pilot-tested and adapted if necessary.

**1) Day 28 questionnaire and blood sample collection**

A structured questionnaire administered to parents/caretakers will elicit the current health status of the child, details on care seeking with special focus on referral and antimalarial treatment, perception of RAS and care seeking cost. It will also include a section on the parent’s/caretaker’s experience and attitude towards the use of RAS and on the results of the mRDT and Hb measurement (see below).

During the home follow-up a finger-prick blood sample will be collected from all of children for confirming that there is no current malaria infection after treatment for severe febrile illness. The following samples will be collected from the finger prick:

- mRDT
- Microcuvette sample for measuring haemoglobin (Hb) concentration

Laboratory procedures for blood samples are described in more detail in Chapter 5.6 ”Biological sample collection and diagnostic tests”. In cases of a positive mRDT, the child will be treated according to standard treatment guidelines or referred to the nearest health facility, if required.

**2) Verbal autopsy**

In case of the death of an enrolled child, the research team will attempt to conduct a verbal autopsy based on the 2016 WHO verbal autopsy instrument [12] with the parents/ caretakers. This will require written consent and will be done at the earliest eight weeks after the child’s death to allow for an adequate bereavement period. This instrument is designed to ascertain and attribute causes of death at the community level and elicit previous treatment seeking. All data collected at this level will also contain the unique study ID of the patient.

Data collected at this level (in combination with data from first contact with CHW / primary health facility) will allow calculating:

- the proportion of severe febrile illness patients not successfully completing referral
- the proportion of severe febrile illness patients treated with RAS as monotherapy
- treatment seeking since first contact with a CHW / primary health facility
- the case fatality ratio among all severe febrile illness patients (and those with a positive RDT at first contact)
- perception of and attitudes towards RAS


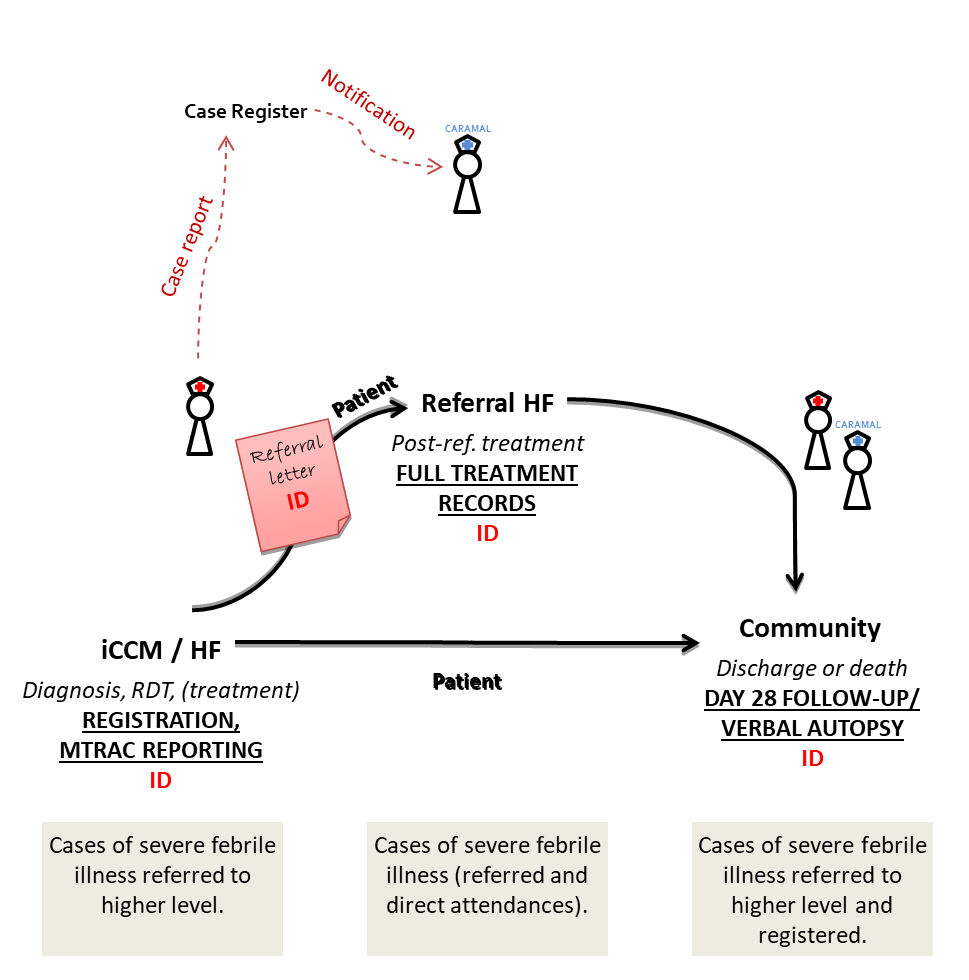


Figure 3. Flow-chart showing the flow of patients and information in the Patient Surveillance System

Table 4. PSS study variables by point of contact

| **Variable** | **Tool** | **CHW** | **Primary HF** | **Referral HF** | **Home** |
| --- | --- | --- | --- | --- | --- |
| Unique study ID |  | Assigned | Assigned | Recorded/ assigned* | Recorded |
| Contact details |  | X | X | X |  |
| Date / time of contact |  | X | X | X |  |
| Name |  | X | X | X | X |
| Demographic details |  | X | X | X | X |
| Signs & symptoms |  | X | X | X | X |
| Danger signs |  | X | X | X | X |
| Diagnostic test results |  | X | X | X | X |
| Treatment |  | X | X | X | X |
| Treatment outcome |  |  |  | X | X |
| Adverse events |  | (X) | (X) | X | X |
| Malaria infection | mRDT | X | X | (X) | X |
| Haemoglobin | HemoCue |  |  | X | X |

*newly assigned to patients who directly visit a referral facility

## Activity 2: Health care provider survey

Health care provider surveys (HCPS) will be conducted annually as a cross-sectional survey in the study areas to assess the availability of diagnostic tools, medicines, medical equipment and human resources. This will also help assess the quality of treatment and care for episodes of severe febrile illness as well as mild episodes of malaria. The surveys will extend across all levels of care using several different data collection tools including checklists and interview guides. Data collected from this activity will inform the following outcomes and their changes over time:

- Access to and rational use of QA RAS as part of the continuum of care
- Case management and referral process
- Acceptability of RAS

### Study population

The HCPS will include a stratified random sample of health care providers, including community-based providers (CHW) and staff at health facilities. The sampling frame will include all registered providers at all levels operating in the study areas, including public and private providers. A simple random sampling approach stratified by provider level (CHW, primary health facility, etc.) will be used to select providers for the survey. As the successful implementation of RAS will depend amongst other factors on the availability of post-referral care, the survey will include all referral facilities in the study area (rather than a sample thereof). Information on the quality of post-referral treatment and care for severe malaria will inform the interventions accompanying the UNICEF-led RAS roll-out.

The following clinical/nursing staff involved in the treatment of children with febrile illness will be

eligible for interviews: i) all selected CHW, ii) at selected primary health care facility, the officers in charge and all other clinical staff, iii) at referral facilities, the officer-in-charge and all clinical staff in the out-patient and paediatric inpatient departments. Per health care provider, up to two staff members per staff category will be randomly selected from a list of all relevant staff and subsequently interviewed.

### Procedures and instruments

Interviews will be conducted by teams of field interviewers who have received extensive training prior to commencing the survey. The training will be led by co-investigators from country research organizations and will consist of lectures on the project background, survey methodology, research ethics, and intensive instruction and practice on the survey protocol and instruments. Members of each field team will spend between one to five days at each participating provider. Prior to any provider visit, the relevant local health authorities will be informed.

At each health facility, the field research team will conduct an information session with the officer-in-charge upon arrival and, following this, with the health facility staff. Once permission to proceed has been obtained, the field team leader will establish in consultation with the officer in charge an acceptable process for survey completion. All interviews will require prior individual informed consent.

Two survey instruments will be completed at each provider. All forms are designed for use with ODK (see Description of data management”, page 49). They will be pilot-tested and adapted if necessary.

**1) Health Care Provider Checklist**

A structured checklist completed with the CHW or officer-in-charge designed to assess the availability of essential medical supplies (incl. RAS) and equipment, human resource capacity, infrastructure and documentation.

**2) Health Care Provider Questionnaire**

An interviewer administered questionnaire completed with CHWs and health workers employed at participating health facilities. The questionnaire contains open and closed questions pertaining to the health worker’s demographics, education and training, work experience and supervision, type and utility of any work-related training received, knowledge, attitudes and practices relevant to febrile case management (incl. diagnostic algorithm and (RAS) treatment guidelines) and intermittent preventive treatment in infants and pregnancy (IPTi, IPTp), experiences implementing malaria/febrile case management and prevention guidelines. More in-depth questions will be asked to health care workers administering RAS as well as different cadres of health workers providing post-referral treatment, focusing on aspects relevant to the implementation of pre-referral RAS and post-referral treatment of severe febrile illness.

Health care providers and health facilities will be identified by a unique ID that is composed of [provider code]-[person ID]. For CHWs [village ID]-[person ID] will be used. The coding will allow linking the two questionnaires applied in this survey.

### Rapid Assessment of referral facilities

A rapid assessment of referral facilities has been conducted by CHAI, UNICEF and local partners in the context of establishing suitable sites for implementing this project and linked to their ongoing collaboration with local health authorities. The purpose of this one-off assessment was to ensure adequate post-referral care is available in a sufficient number of referral facilities before RAS is implemented and referral is promoted. The data will be used to inform the CARAMAL team and other stakeholders including UNCEF and the national malaria control programmes on implementing limited supportive interventions, as needed to ensure minimal functionality. Such interventions could include improving diagnostic facilities, strengthening of the commodity supply chain, or training of staff in the provision of severe malaria treatment and care.

## Activity 3: Household survey

Cross-sectional household surveys will be carried out annually in the study areas in order to assess (1) malaria control intervention coverage, (2) treatment seeking patterns, (3) caretakers’ knowledge of and attitudes towards RAS, (4) socio-economic status and other community characteristics, and (5) prevalence of malaria and anaemia. Data collected from this activity will inform the following outcomes and changes in these outcomes over time:

- Patterns of illness (at community level)
- Treatment seeking from different sources for mild and severe malaria episodes
- Caretakers’ knowledge of and attitudes towards RAS
- Prevalence of malaria and anaemia in children < 5 years

Contextual information on malaria control intervention coverage and demographic and socio-economic characteristics of the household will allow assessing crucial determinants for rates of severe malaria disease. For this reason, a finger prick blood sample will be taken from every household member below 5 years of age to assess changes in malaria prevalence and anaemia over time in the study areas (see “Biological sample collection and diagnostic tests”, page 44**)**.

### Study population

Household surveys will include randomly sampled households in the study areas. Households will be selected using a two-stage random sampling approach (village-household) whereas the sampling frames will consist of all villages in the study area and all households in the village with at least one child < 5 years, respectively.

Within each sampled village a list of households will be established by the survey team and local village representatives. In settings where village-level census data is available, probability-proportional-to-size (PPS) sampling will be applied.

In each household, the household heads and parents/caretakers of children < 5 years of age will be eligible to participate in face-to-face structured and semi-structured interviews. Blood samples by finger prick will be collected from all children < 5 years of age provided a parent/caretaker consents.

### Procedures and instruments

Interviews will be conducted by teams of field interviewers who have received extensive training prior to commencing the survey. The training will be led by co-investigators from country research organizations and will consist of lectures on the project background, survey methodology, research ethics, and intensive instruction and practice on the survey protocol and instruments. At least one of the team members must be qualified by local guidelines to collect finger-prick blood samples.

Prior to any field work, relevant local authorities will be informed following locally acceptable standards. Upon arriving at each survey village, the field team will inform and secure permissions from relevant village authorities/community leaders and community members, where applicable.

A listing of all households in the respective village will be established in consultation with village leader(s) and guides, wherever such listing does not exist. The random selection of households will then be performed by the team leader using a random number list and the survey process will commence. An alternative approach using satellite images may also be applied.

Three survey instruments will be completed with participating household heads and/or household members:

Face-to-face interviews with eligible adult respondents (household heads and parents/caretakers of children < 5 years) will be conducted using structured electronic data collection forms in ODK (see “Description of data management”, page 49). All interview questionnaires will be pilot-tested and adapted if necessary.

**1) Household Questionnaire**

A structured interviewer administered questionnaire completed with adult heads of selected households. This form collects information about coverage and uptake of mosquito nets behaviour change campaigns and other malaria control interventions, alongside background demographic information on each household member as well as indicators of the household’s socio-economic status.

**2) Treatment Seeking Questionnaire**

A semi-structured interviewer administered questionnaire completed with randomly selected household members who are parents or caretakers of a child <5 years of age. Parents/caretakers of children who reported a febrile illness (irrespective of severity) in the two weeks prior to the survey will be interviewed about that specific illness episode. The form collects information about the signs and symptoms of the illness and subsequent treatment seeking behaviours, including sources of treatment and types (if any) of drugs administered. Parents/caretakers of children who did not experience a recent febrile illness will be asked about hypothetical care seeking behaviour based on specific vignettes (one scenario of mild and one scenario of severe febrile illness). The form furthermore collects information on the knowledge and attitudes of the parents/caretakers towards RAS. The parent/caretaker will be asked about previous experiences with RAS. Parents/caretakers without any previous experience with RAS will be asked about their attitudes based on a vignette. Short qualitative components in the interviews may be audio recorded, transcribed verbatim and coded.

**3) Prevalence Form / blood sample collection**

A short form will accompany each finger-prick blood sample, recording the individuals’ demographic details, recent travel history, intake of any medicine, the mRDT and Hb measurement result, and any treatment administered.

Households and household members interviewed will be identified by a unique ID that is composed of [village ID]-[household ID]-[person ID], whereas the latter is the “line number” of the household members listing in the main Household Questionnaire. The coding will allow linking the two questionnaires applied in this survey.

## Activity 4: Economic evaluation study

The economic evaluation will assess financial costs and the incremental cost-effectiveness of QA RAS introduction over current standard of care for management of severe malaria. Financial and a few essential non-financial economic costs (such as salaries) will be collected continuously during the project period. Most cost details will be extracted from the routine accounting documents of the iCCM projects in the three countries. Additional data will be collected in the frame of other surveys (for example time spent by providers on caring for a child (HCPS), and financial costs incurred by caretakers during referral (PSS Day 28 assessment)). This activity will not include the full economic cost incurred during a disease episode, for example the productive time lost by caretakers. The evaluation will reflect multiple perspectives including individual (i.e. patient), societal, and health systems-level (i.e. government). The methodology will follow standard economic evaluation guidelines [13]. We will use an ingredients-based approach to quantify the resources and associated unit costs required to implement QA RAS, as well as those related to the current standard of care for management of severe malaria. We will organize costs according to standard expenditure categories, including: drugs, personnel (salaries), supplies, equipment, services, space, and overhead. Full incremental costs will be derived, measured in the local currency, and converted to year-adjusted US dollars.

## Activity 5: Routine monitoring of process indicators

Programmatic records of the implementation of QA RAS by UNICEF will provide the backbone for routine monitoring of process indicators. These records will provide data on QA RAS implementation progress and contextual variables for evaluating the effect of the QA RAS roll-out. A number of different data collection methods will be accessed, including:

1. **RAS needs assessment data and orders**

Procurement records will provide information on the annual number of QA RAS units procured and distributed to projects areas.

1. **Reports from supportive supervision**

Supportive supervision conducted by the implementing agency UNICEF (and/or government health officials) will support the implementation of QA RAS and provide information on functionality of community based providers of QA RAS, incl. number and proportion of trained and functional providers and of providers with QA RAS in/out of stock.

1. **CHW monthly reports**

Monthly routine reports submitted by CHW to the implementing agency UNICEF will provide details on the proportion of trained CHWs and peripheral health facilities actively administering RAS.

Provider geo-locations (CHW and health facilities) will allow spatial analyses of QA RAS availability.

1. **CHW, health facility registers**

Routine registers of health workers will provide data on trends in uncomplicated and severe malaria cases over times.

Patient registers kept by community based providers will allow monitoring trends over time in the number and proportion of children treated with QA RAS, their demographic characteristics, and (to a certain degree) on the off-label administration of QA RAS.

Routine registers kept at referral facilities will capture the number of children with severe febrile illness who received pre-referral QA RAS including their demographic details, test results and treatments administered.

Programmatic data collection instruments will be reviewed on a continuous basis and selected indicators transcribed into an electronic study database.

## Biological sample collection and diagnostic tests

Capillary blood samples will be collected by finger-prick or heel-stick from children < 5 years of age with signs of severe febrile illness for the diagnosis of malaria by CHW and primary health facility staff. A malaria rapid diagnostic test (mRDT) will be performed. The mDRT will be supplied through established supply channels. Procedures for performing the test will follow the manufacturer’s protocol and local iCCM guidelines. This procedure is part of routine diagnostics for febrile illnesses in all three project countries. Where CHW have not previously routinely performed mRDTs, training will be conducted in the frame of the malaria case management and RAS training module provided by UNICEF.

A further blood finger-prick / heel-stick blood sample will be collected by a trained member of the study field team during household follow-up visits 28 days after enrolment into the PSS. An mRDT will be performed, as well as an Hb assessment, a good morbidity measure in malaria. An HRP2/pLDH test will be used meeting the WHO criteria for selecting and procuring rapid diagnostic tests for malaria [14] in consideration of the latest available WHO product testing result (currently [15]). . Hb tests will be performed using a Hemocue (Sweden) photometric analyser on site as per the manufacturer’s instructions.

During the household survey, a blood finger-prick / heel-stick blood sample will be collected by a trained member of the study field team in order to assess the prevalence of malaria infection in children <5 years in the community. An mRDT will be performed and the Hb level measured, as described above. The prevalence assessment is important for monitoring trends in malaria transmission intensity over time, an important covariate in the analyses of CFR and related indicators. The Hb measures will provide important information on the evolution of anaemia levels over time.

One finger or heel prick will yield sufficient blood for all samples and special care will be taken to avoid multiple punctures.

All blood samples will be immediately destroyed after the respective test result is obtained and documented.

Staff will undergo adequate training in blood collection and test execution and interpretation.

## Environmental temperature

Electronic temperature loggers will be used to record the environmental temperature in the location where RAS packages are kept at community-based providers and health facilities. Temperature will be monitored over several periods of time throughout the year to establish seasonal fluctuations and peak temperatures during different seasons. Stored temperatures will be downloaded to computers by field supervisors.

## Measures to minimize bias

The present study is an observational study based on a before-and-after plausibility design [6], with no treatment randomization and no external control group. The focus of the present work is to document the process of optimizing the continuum of care for severely ill children including RAS and other health systems measures under routine, real-world circumstances.

The selection of health care providers, households and individuals included in the cross-sectional surveys will be based on (simple, stratified, or PPS) random sampling with the aim to minimise selection bias. Special attention will also be paid to minimising interviewer or measurement bias by thoroughly training field staff and implementing standard operating procedures for all study components. During the informed consent process, special attention will be given to a non- judgemental attitude of the research team while explaining that the researchers’ objectives include obtaining information on health care seeking behaviour, practices, attitudes, and beliefs regarding severe malaria reflecting as much as possible the reality.

Treatment seeking interviews during the household surveys (dealing with both uncomplicated and severe disease episodes) will cover the past four weeks; the precise timing of the episode will be established in an effort to consider recall period as potential confounder in the analyses. The same recall period will be applied in the context of the day 28 follow-up. This activity will exclusively investigate severe cases for which recall is likely to be better. Since the case mix in both surveys will be rather different, no attempt will be made to directly compare them.

Triangulation of information from household surveys, health care provider surveys and the different contact points in the PSS will help to detect any major bias in one of the data collection components. In the PSS, not all patients will be seen by a study nurse at a referral facility due to the referral not being completed or because the patient went to a referral facility outside the study area. Different levels of detail of information will therefore be available from patients seen and patients not seen at the referral facility. In order to minimise any bias in comparing these patients, variables for comparison will have to be selected carefully. The validity of day 28 information can be checked by comparing self-reported data with data collected by the study nurses in the referral facilities.

## Study duration

The project will be implemented in a pre-RAS baseline phase of approximately 6 months and a post-RAS implementation phase of approximately 18 months.

|  | **2017** | **2018** | | | | **2019** | | | | **2020** | | | |
| --- | --- | --- | --- | --- | --- | --- | --- | --- | --- | --- | --- | --- | --- |
|  | **Q4** | **Q1** | **Q2** | **Q3** | **Q4** | **Q1** | **Q2** | **Q3** | **Q4** | **Q1** | **Q2** | **Q3** | **Q4** |
| **Project phase** | **Pre-RAS** | | | | **R/O** | **Post-RAS introduction** | | | | | | | |
| IEC submission | X |  |  |  |  |  |  |  |  |  |  |  |  |
| IEC approval |  | X |  |  |  |  |  |  |  |  |  |  |  |
| **Baseline data collection** |  |  | **6 months** | |  |  |  |  |  |  |  |  |  |
| PSS |  |  | X | X | (X) |  |  |  |  |  |  |  |  |
| HCPS |  |  | X | X |  |  |  |  |  |  |  |  |  |
| HH survey |  |  | X | X |  |  |  |  |  |  |  |  |  |
| Costing study |  |  | X | X |  |  |  |  |  |  |  |  |  |
| Routine data collection |  |  | X | X | X |  |  |  |  |  |  |  |  |
| Baseline data analysis |  |  |  | X | X |  |  |  |  |  |  |  |  |
| **Post-RAS data collection** |  |  |  |  |  | **18 months** | | | | | |  |  |
| PSS |  |  |  |  |  | X | X | X | X | X | X |  |  |
| HCPS |  |  |  |  |  |  | X | X |  |  | X |  |  |
| HH survey |  |  |  |  |  |  | X | X |  |  | X |  |  |
| Costing study |  |  |  |  |  | X | X | X | X | X | X |  |  |
| Routine data collection |  |  |  |  |  | X | X | X | X | X | X |  |  |
| Post-RAS data analysis |  |  |  |  |  |  |  | X | X | X | X |  |  |
| Publication and technical manuscripts |  |  |  | X | X |  |  | X | X |  | X | X |  |
| Data Quality Audits |  | X |  |  |  | X |  |  |  | X |  |  |  |

IEC = Independent Ethics Committee; PSS = Patient Surveillance System; HCPS = health care provider survey; HHS = household survey; R/O = QA RAS roll-out

### Early termination of the study

The Sponsor-Investigator, Project Lead and any competent authority may terminate the project prematurely according to certain circumstances (e.g. insufficient participant recruitment, ethical concerns, financial issues, change of principal investigator or any other reason that would prevent the project execution according to the research plan). Reasons for early termination are stipulated in the Consortium Agreement between the implementing partners CHAI; Swiss TPH and UNICEF.

# Selection of the study participants

## Recruitment

In the frame of introducing QA RAS at the community level, available iCCM communication and mobilization strategies will be leveraged to raise awareness about the availability of QA RAS and the importance of immediate and completed referral. Communities will be informed via the same communication channels about the research activities relating to RAS implementation.

Cases of severe febrile illness eligible for enrolment in the PSS will be recruited among patients presenting to the designated iCCM CHW / peripheral health facilities or seeking care directly at the referral health facility within the project area. A person can repeatedly be part of the study, either through repeated visits to a CHW / health facility for an episode of severe febrile illness, or by being recruited in more than one study component.

CHWs, health workers at peripheral health facilities and health care providers at referral hospitals will be recruited among all health workers involved in the iCCM referral system in the project areas for face-to-face interviews, according to the procedures described above.

During household surveys, heads of households and parents/caretakers of children < 5 years will be recruited from among all de facto members of sampled households, according to the procedures described above. .

## Inclusion criteria

Patient surveillance system:

- Children < 5 years
- History of fever plus danger signs indicative of severe febrile illness / suspected severe malaria, according to local iCCM guidelines
- Child referred to higher level facility by CHW/primary health facility, or, child directly attending a referral facility.

Signed full consent form from parent / guardian

Health care provider interview:

- CHW or health worker at peripheral health facility enrolled in the iCCM referral system who treats children < 5 years OR
- Health care provider at referral hospitals treating children < 5 years OR
- Any other health care provider in project area treating children < 5 years
- Signed consent form

Household survey:

- Household head and parent / caregiver of children < 5 years
- Signed consent form from parent / guardian

## Exclusion criteria

Patient surveillance system:

- Children ≥ 5 years
- Children with no permanent residence in project area

Health care provider interview:

- Health workers not treating children < 5 years
- Health workers outside project area
- Health workers who do not speak any of the local languages
- Health workers employed since < 1 month

Household survey:

- Parents / guardians with no children < 5 years
- Parents / guardians with no permanent residence in project area
- Parents / guardians who do not speak any of the local languages

# Description of data management

## Data collection

In collaboration with UNICEF, Swiss TPH will ensure as much as possible uniformity of data collection tools across study sites in the three countries. Swiss TPH will ensure data quality through regular data quality audits described in more detail in Chapter 11, “Quality control and quality assurance: description of measures”.

Quantitative data collected by field research teams and study nurses will as much as possible directly be captured on internet/Wi-Fi capable tablets using OpenDataKit (ODK) electronic data collection software (see 7.2 “Data management system”). Quantitative data for which electronic data capture is not feasible and qualitative data will be captured on paper forms (and audio recorded if applicable) and entered into an electronic database. Double-entry will be required for all datasets collected on paper. Depending on the country-specific context this may only be relevant for primary records from CHW and primary health facilities which are collected during CHW-supervisor visits. In these cases, data entry will be coordinated with the implementing agency.

In the PSS, data will be derived from source documents and consistency of data transcription will be ensured by regular spot-checks. The Country Research Partners will ensure that a routine of cross-checks with source documents is established. Source documents include participant CHW / health facility records, doctors’ and nurses’ notes, appointment book, original laboratory reports etc. Primary data collection tools and referral forms will allow unique identification of an individual child eligible for pre-referral RAS treatment. Referral documents will use the same unique identifier to allow follow-up of post-referral treatment as well as capture of final outcome (recovered, recovered with sequelae, dead, still sick). The assignment of the unique study ID has been described in chapter 5 “Project design”.

During cross-sectional surveys (health care provider and household surveys), households, health facilities and individuals will be assigned a unique ID as described in chapter 5 “Project design”. The unique ID will allow linking the different data collection instruments used under the same activity.

## Data management system

Swiss TPH will be responsible for overseeing all aspects of data management. **Study data** will be collected using customized entry screens on password-protected tablets equipped with OpenDataKit (ODK) software.

Open Data Kit (ODK) is a free open-source software tool for form-based data collection using mobile devices (http://opendatakit.org/). It has a flexible user interface that allows the user to easily create forms for use on Android smart phones or tablet computers. It uses a secure username/password authentication process. Data collected on a mobile device can be uploaded to a server and data aggregation and processing is done through a web application, which can be accessed (password-protected) from anywhere in the world. Constant mobile network connection is not required for this application, as data is automatically saved on the mobile device and can be sent whenever connectivity can be established. ODK has been established as a useful tool for electronic data collection for surveillance and epidemiological studies in resource-poor settings [16, 17]. ODK forms and data download procedures will be pretested in each country and amendments will be made if necessary.

The programming of tablets for data collection and databases will be completed by Swiss TPH jointly with country research partners. Data collected on tablets will be transferred to the study database on a daily basis when a reliable internet connection is available, and at the earliest opportunity when internet access is unavailable/problematic (e.g. in remote field locations). Individual data on each tablet will be routinely erased after confirmation of a successful data transfer to the study database.

Electronic and routine anonymised **M&E data** collected by UNICEF will be imported into the study database under consideration of validating the process and maintaining data integrity. Swiss TPH and UNICEF will ensure data transmission follows national data flow process and quality data is assessed through regular supervision (including spot checks). With UNICEF’s support, a monthly/quarterly data review meeting will be organized by the district management team along with key health-players prior to data/report submission.

Where considered necessary, and after obtaining the relevance clearance, routine Health Management Information Systems (HMIS) data may be accessed on rare occasions and transferred into the database.

In order to minimise data recording errors, ODK form fields will specify entry constraints that limit the values to plausible ranges. These constraints will be pre-tested as part of the overall pilot testing of all forms. Databases will be cleaned by thoroughly checking whether indicator values fall within plausible ranges, confirming whether skip patterns have been respected, and assessing whether survey responses are consistent with previous responses. The database will be locked after all data was monitored and all raised queries have been resolved.

Information recorded as narratives in a local language will be translated to English by the country research partners.

All data collected during this research will be hosted on a secure encrypted server located at Swiss TPH for the duration of the proposed research. Data Transfer Agreements (DTA) will be filled by all partners to facilitate data transfer.

## Data security, access, archiving and back up

All source documents must be stored for possible review and/or audit by Swiss TPH and/or local health authorities. All study data must be archived for the minimum of time specified by local legislation after study termination or premature termination of the research project. Paper data collection forms will be stored in lockable cabinets in the country research organization’s offices or, during the study, in referral facilities.

All cost data information will be collected on pass-word protected Excel spreadsheets, and treated with the same level of confidentiality as the clinical data. Only the study personnel in charge of this component and working together with the financial managers of the implementing organizations will have access to the collected data. Data made public will be aggregated in such a way that data cannot be linked to either patients or individual health care workers. In addition, any paper files and hard disk backups will be kept in locked drawers.

M&E data collected by UNICEF: as per local HMIS guidelines, the paper based tools are stored at collection points (CHW/VHT and health facility level). Data summaries are stored at the district health office. UNICEF will ensure all paper-based data has been digitalized and backed-up.

Direct access to original study data will be limited to study personnel. Analysis databases will be anonymised by deleting all identifiable information such as names of patients or interview participants. No names will be published at any time, and published reports will not allow the identification of single study participants. Confidentiality and anonymity will be ensured throughout the entire research project.

# Statistics

## Hypothesis

Pre-referral QA RAS can be implemented safely and cost-effectively at scale in real-life settings reducing CFR for severe malaria in cases seen by CHWs or at peripheral health facilities.

## Determination of sample size

### Sample size calculation for case fatality ratio (CFR)

Sample size calculations were performed for precision of the point estimate of CFR in severe malaria cases that make contact with the primary health care system, and for comparing pre- and post-intervention proportions. The latter is the analysis requiring the largest sample size and it was therefore the starting point for our calculations. We assumed the CFR to be 6% at baseline (historical case-fatality rates for severe malaria: 2.8% MATIAS Study DRC [18], 8.5% AQUAMAT [9]) and the data collection to last for 6 months pre-RAS (baseline) and 18 months post-RAS.

The minimum sample size of **6,032 cases of severe malaria in children < 5 years** over 24 months is based on the calculation of difference in proportions allowing the detection of a 30% decrease in case fatality across the three project countries following the roll-out of RAS (target in Unitaid grant agreement) with 80% power and α = 0.05 (Table 5). Based on the project time plan with 6 months of baseline and 18 months post-RAS (total 2 years, allocation ratio = 3), this translates into 1508 cases at baseline and 4524 after RAS roll-out. The sample size calculation formula is provided below; it is based on Fleiss et al. [19] with an allocation ratio of 3, and implemented in STATA using the 'power twoproportions p1 p2, nratio(3)' command.


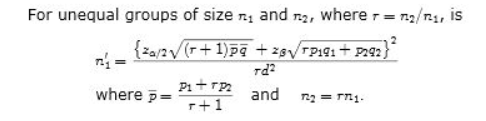


Table 5. Sample size calculation for difference in CFR

| **Baseline** | **Delta** | **Post-RAS** | **N baseline** | **N post-RAS** | **N total** |
| --- | --- | --- | --- | --- | --- |
| 6% | -10% | 5.4% | 15,468 | 46,404 | 61,872 |
| 6% | -20% | 4.8% | 3,632 | 10,896 | 14,528 |
| **6%** | **-30%*** | **4.2%** | **1,508** | **4,524** | **6,032** |
| 6% | -35% | 3.9% | 1,069 | 3,207 | 4,276 |

* target in Unitaid grant agreement

This sample size will also allow an acceptably precise estimate of the actual point estimate of CFR relative to the value hypothesised (H0) based on existing literature (Table 6).

Table 6. Sample size calculation for precision of CFR point estimate

| **H_0_** | **Delta** | **Actual value** | **N** |
| --- | --- | --- | --- |
| 10% | 0.5 | 10.5% | 28,628 |
| 10% | 1 | 11.0% | 7,248 |
| 10% | 1.5 | 11.5% | 3,261 |
| 10% | 2 | 12.0% | 1,856 |
| 6% | 0.5 | 6.5% | 18,115 |
| 6% | 1 | 7.0% | 4,627 |
| 6% | 1.5 | 7.5% | 2,099 |
| 6% | 2 | 8.0% | 1,204 |
| 3% | 0.5 | 3.5% | 9,566 |
| 3% | 1 | 4.0% | 2,493 |
| 3% | 1.5 | 4.5% | 1,151 |
| 3% | 2 | 5.0% | 671 |

On this basis, using rounded numbers of 1,600 cases at baseline (over 6 months) and 4,600 post-RAS (over 18 months), the required size of the overall study area across 3 countries was then calculated by assuming 18% of the population to be children <5 years^[[5]](#footnote-6)^ and a severe malaria rate of 0.02/year^[[6]](#footnote-7)^. We also assumed that only 40% of all severe malaria cases would be enrolled in the PSS at CHW/primary health facility level and followed up on Day 28. This assumption takes into consideration that not all severe cases seek treatment from a CHW or a peripheral facility, as some may directly attend a referral facility or not seek treatment at all and some patients will be lost to follow-up by Day 28. For the purpose of measuring changes in CFR, a pooled analysis with data from all three countries will be undertaken, while other indicators will be calculated per country.

In order to detect a 30% decrease in case fatality across the three project countries, the project area should comprise a total population of roughly **2.2 million** people, including **400,000 children <5 years** of which 3,200 are expected to be registered with a severe malaria episode over a hypothetical 12-months or 1,600 over the 6-months pre-RAS baseline data collection period, respectively (Table 7). The study areas chosen in the project countries can therefore be considered sufficient, with the smaller area in DRC being outweighed by the larger areas in Nigeria and Uganda.

Table 7. Estimation of required size of project area to detect a 30% decrease in CFR across the three project countries

### Sample size calculation for successful completion of referral

Sample size was further calculated per country for a comparison of the proportion of children with severe febrile illness, seen by a community-based provider, who are successfully referred pre- and post-implementation of RAS.

Successful referral was assumed to be between 15 and 25% at baseline (17). The sample size was calculated to allow for a detection of various degrees of improvement with 80% power, α = 0.05, 6 months of baseline and 18 months post-RAS (total 2 years; sample ratio = 3). Depending on the assumptions, a sample size of between 108 and 3372 severe malaria cases is required over the entire project period (Table 8).

Table 8. Sample size calculation for difference in proportion successfully referred

| **Baseline** | **Post-RAS** | **N baseline** | **N post-RAS** | **N total** |
| --- | --- | --- | --- | --- |
| 15% | 20% | 617 | 1851 | 2468 |
| 15% | 30% | 84 | 252 | 336 |
| 15% | 40% | 34 | 102 | 136 |
| 20% | 30% | 200 | 600 | 800 |
| 20% | 40% | 56 | 168 | 224 |
| 20% | 50% | 27 | 81 | 108 |
| 25% | 30% | 843 | 2529 | 3372 |
| 25% | 35% | 223 | 669 | 892 |
| 25% | 40% | 104 | 312 | 416 |

A study area based on the CFR-based sample size of 1,600 cases at baseline (533 per country) and 4,600 post-RAS (1533 per country) will allow detecting most projected changes in referral at an individual country level. It will furthermore allow an acceptably precise estimate of the actual point estimate of successful referral relative to the value hypothesised (H0) based on existing literature (Table 9).

Table 9. Sample size calculation for precision of point estimate of successful referral

| **H_0_** | **Delta** | **Actual value** | **N** |
| --- | --- | --- | --- |
| 20% | 5 | 25% | 528 |
| 20% | 10 | 30% | 137 |
| 20% | 20 | 40% | 36 |
| 20% | 30 | 50% | 17 |
| 30% | 5 | 35% | 676 |
| 30% | 10 | 40% | 172 |
| 30% | 20 | 50% | 44 |
| 30% | 30 | 60% | 20 |

### Sample size calculation for Health care provider surveys

Coverage indicators considered for this calculation are availability of QA RAS and adherence to case management guidelines incl. referral. The sample size was estimated to allow for detecting a decrease in the minimum acceptable coverage following the roll-out of RAS.

Due to the relevance of the quality of severe malaria treatment provided at referral facilities for the roll out of QA RAS by UNICEF and partners, the surveys will include all referral health facilities in the project areas. In Uganda and DRC, the survey will also include all non-referral health facilities in the study are, while in Nigeria, a random sample of 40 facilities will be included, allowing for the detection of a reduction of coverage indicators by 19 percentage points from a baseline value of 80% with a power of 80% and α = 0.05.

In addition, 40 community health workers will be included in Nigeria and Uganda, allowing for the detection of a reduction of coverage indicators by 19 percentage points from a baseline value of 80% with a power of 80% and α = 0.05. In DRC, all CHW will be included due to their smaller number (<40).

Other indicators, such as acceptability of RAS are exploratory and more qualitative in nature. For the purpose of assessing these indicators, up to two health workers per cadre will be interviewed per selected health facility.

### Sample size calculation for household surveys: treatment seeking from CHF/primary health facility

The sample size for household surveys is based establishing treatment seeking rates from community-based providers and detecting changes between baseline and post-implementation of RAS. Estimates of treatment seeking for fever in children <5 years from formal health facilities vary greatly between studies and can be estimated as low as 15% (16) to over 75% [21]. For the purpose of calculating a sample size for comparing proportions pre- and post-implementation of RAS, treatment-seeking at baseline was assumed to be as low as 15% even though we assume that in reality it is considerable higher. To allow for a detection of an increase to 20% with 80% power and α = 0.05, a minimum sample size of 906 household survey responses on treatment-seeking for severe febrile illness will be required per country and individual survey round.

## Description of statistical methods

Data analyses will be performed in Stata (StataCorp LP, College Station, TX, USA), SAS (Carey, NC), R or similar software packages. Data analyses will be performed by country research partners and Swiss TPH.

### Descriptive analysis of routine M&E data

Country research partners will take on primary responsibility for the descriptive analysis of routine M&E data and other relevant variables for their respective countries, with support from Swiss TPH. The monitoring of intervention coverage will be used as a management tool by the programmes.

Descriptive analyses of point estimates of primary and secondary operational indicators will be performed once after baseline (pre-RAS) and twice during the post-RAS period, with a first post-RAS analysis 9 months after RAS roll-out. This will entail constructing tables and graphics of operational indicators disaggregated by administrative area, health facility, or CHW. Most variables will be presented as proportions, but numbers of events recorded at health facilities will be expressed as population-based rates. This analysis will directly inform the continuous roll-out of RAS in the three countries.

### Inference of the effects of the RAS roll-out

Swiss TPH will take primary responsibility for overall analyses (in Q3 2020) of the final compiled dataset, in close collaboration with country research partners. A standardised inter-sites analysis is essential in order to obtain point and interval estimates of the impacts of the RAS program, and to test their statistical significance.

Standard methods of survey statistics will be used to analyse the health care provider and household surveys to establish the availability and uptake of QA RAS at all levels, and health providers’ knowledge, attitudes and practices towards RAS, treatment seeking behavior, and malaria intervention coverage. Individual-level data will be tabulated by province, age, sex, and SES (as measured by asset indices derived from the household surveys). Most of the indicators to be analyzed are proportions. Interval estimates for these will be computed with allowance for both the stratification and clustering in the sampling design, and will be tabulated separately by country, administrative area, and programme status. Random effects logistic regression, or similar approaches, will be used for significance testing of the impact of the RAS program, controlling for relevant covariates including trends in coverage of other malaria control interventions. For outcomes that are not proportions, analogous approaches based on normal models (or ranks, where there are substantial deviations from normality) will be applied. Hypothesis testing will use likelihood ratio tests with a significance level (α) of 0.05.

The analysis of the clinical outcomes of the RAS roll-out will be done primarily on a pooled dataset across all three countries, due to the expected low number of relevant cases and deaths. A cohort, consisting of all the severe malaria cases that contact the CHWs and primary health facilities will be defined. Initial analysis will apply a therapeutic efficacy study approach, taking the cohort that presents with severe fever (and positive RDT) at the CHW/primary health facility as denominator and the Day 28 health status as final outcome measure. This will provide an initial estimate of the CFR.

To allow for loss to follow-up, and making use of additional data available from the referral health facilities further analyses will be conducted. At each level of facility there are a series of possible outcomes for cases that present and are identified (cure, death, further referral), together with a set of possibilities for those that are not identified (did not report, not identifiable owing to data linkage error, not identified as a suppository recipient, etc..). The complete set of probabilities of the different paths and outcomes specify a decision tree. The effectiveness ladder corresponding to this tree will be analysed using a Markov process model, using standard methods (either by maximum likelihood, or using a Bayesian MCMC algorithm). The MCMC approach will make it possible to combine follow-up data for the cohort from the health facilities, with the data from the 28 day home visits, while including cases that are lost to follow-up. It will also enable the estimates to be updated with information from the health care provider and the household surveys.

In addition to the basic analyses of key indicators listed in this protocol, further in-depth analyses will be conducted as per emerging requirements by project partners and in-country stakeholders. These analyses will be informed by findings from basic descriptive analyses and by questions arising during the implementation of the project.

Hypothesis testing will be used to determine whether or not absolute and relative target values of outcomes and impact indicators have been achieved.

## Handling of data

The potential for missing data in this study would arise from

- Participants enrolled in the PSS that cannot be retrieved for or refuse to take part in the D28 FU visit
- Participants’ refusal to answering questions during the interviews

Missing or spurious data will be accounted for in our statistical analysis. Answer options like “not known” or “does not wish to answer” will be included in the questionnaires.

In the event of withdrawal of informed consent or participant discontinuation, no further evaluations should be performed and no attempts should be made to collect additional data. Data obtained prior to participant withdrawal will still be included in the analysis.

# Duties of the Investigator

## Investigator’s confirmation

All protocol modifications must be documented in writing. A protocol amendment can be initiated by either the Sponsor or any Investigator. The Investigator will provide the reasons for the proposed amendment in writing and will discuss with the Sponsor and the Principal Investigator. Any protocol amendment must be approved and signed by the Sponsor and the Principal Investigator and must be submitted to the appropriate Independent Ethics Committee (IEC) for information and approval, in accordance with local requirements, and to Health/Regulatory Authorities if required. Approval by IEC (and Health/Regulatory Authority, if applicable) must be received before any changes can be implemented, except for changes necessary to eliminate an immediate hazard to study participants, or when the change involves only logistical or administrative aspects of the study, e.g. change of telephone number(s).

## Safety reporting

Safety reporting will be limited to events concerning haemoglobin levels (see Chapter 11.1.1 Risk identification, assessment and mitigation); all other clinical information is out of the scope of this study.

## Project management

| **Prof. Dr. Christian Burri** | **Swiss TPH**  Head, Department of Medicines Research;  Professor of Pharmacy and Clinical Pharmacology, University of Basel | Responsible for conduct of research activities according to ethical guidelines. Contributes to development of research protocol development. Serves as main contact for ethics committees and drug regulatory authorities. Contributes to preparation of scientific and technical manuscripts. Supports WHO-GMP in the process of convening an ERG and developing QA RAS implementation guidance. |
| --- | --- | --- |
| **Prof. Dr. Christian Lengeler** | **Swiss TPH**  Head, Health Interventions Unit, Department of Epidemiology and Public Health;  Professor of Epidemiology, University of Basel | Overall scientific lead, key contact for study design and writing of protocol. Leads the process of documenting findings and lessons learnt through the project. Leads writing of progress reports and dissemination of results to partners. Supports WHO-GMP in the process of convening an ERG and developing QA rectal artesunate implementation guidance. |
| **PD Dr. Manuel Hetzel** | **Swiss TPH**  Scientific Project Leader,  Health Interventions Unit, Department of Epidemiology and Public Health | Country lead: Nigeria, Uganda. Leads development and setting up of the PSS and household surveys. Leads development, setting up and analysis of baseline and impact data collection, including databases. Contributes to development of research protocol. Leads preparation of scientific and technical manuscripts. |
| **Dr. Aita Signorell** | **Swiss TPH**  Clinical Research Scientist, Research Cluster, Department of Medicine | Country lead: DRC.  Leads the development and setting up of clinical assessments in the PSS and health care provider surveys. Coordinates routine monitoring of process indicators between Consortium Partners and countries. |
| **Prof. Antoinette Tshefu** | **University of Kinshasa**  Professor, Kinshasa School of Public Health | Sub-Investigator DRC, responsible for data collection and analysis and for documenting findings at national level |
| **Dr. Elizabeth Omoluabi** | **Akena Associates Ltd.**  Director | Sub-Investigator Nigeria, responsible for data collection and analysis and for documenting findings at national level |
| **Dr. Phyllis Awor** | **Makerere University**  Research Fellow, School of Public Health | Sub-Investigator Uganda, responsible for data collection and analysis and for documenting findings at national level |

# Ethical considerations

The research project will be carried out in accordance with the research plan outlined in this protocol and with principles enunciated in the current version of the Declaration of Helsinki, International Conference on Harmonisation ICH Topic E6: ‘Guideline for Good Clinical Practice’. 1996 (CPMP/ICH/135/95), Essentials of Good Epidemiological Practice issued by Public Health Switzerland (EGEP) as well as all national legal and regulatory requirements as applicable.

GCP E6(R2) is specifically designed to allow interpretation and flexibility to accommodate several types of investigations. The present work is operational, and there is a low risk for participants in this study arising from the study. Studying the non-experimental, real life implementation of an intervention study with a low risk for participants requires the collection of unbiased and uninfluenced data under true field conditions. The CARAMAL project will fully adhere to the Declaration of Helsinki 2013 and be compliant to GCP E6(R2), but it will make full use of the flexibility provided by the revised current version GCP E6(R2) and the European clinical trial regulation 2014 foreseen particularly for such studies and situations.

All necessary measures will be taken to ensure gender equality throughout the conduct of this research project. We do not foresee any risk of gender bias regarding the treatment of patients and survey participants enrolled in this study.

## Independent Ethics Committee (IEC)

Before study initiation, the protocol will be submitted together with its associated documents, to the responsible IEC/IRB for its favourable opinion/approval. This includes CHAI’s Scientific and Ethical Review Committee and the WHO Research Ethics Review Committee.

Furthermore, the protocol will be formally submitted for approval by the local Sub-Investigators to each country’s IEC/IRB, namely:

- Uganda: Makerere University School of Public Health Research Ethics Committee (REC) and Uganda National Council for Science & Technology (UNCST)
- Nigeria : Ethics Committee of Adamawa State,
- DR Congo: Kinshasa School of Public Health Ethics Committee, University of Kinshasa

The study will not start at a site before the Sponsor-Investigator has obtained written favourable opinion/approval from the concerned IEC/IRB and authorities. The IEC/IRB will be asked to provide documentation of the date of the meeting at which the favourable opinion/approval was given, the members and voting members present at the meeting. Written evidence of favourable opinion/approval that clearly identifies the study, the study protocol version and the Participant Information and Informed Consent Form version reviewed should also be provided.

Any amendments to the study will also be submitted to the concerned IEC/IRB and the authorities, before implementation. Relevant safety information will be submitted to the IEC/IRB and the authorities during the course of the trial in accordance with national regulations and requirements.

## Evaluation of the risk-benefit ratio

### Risk categorisation

The present work is in risk category A of the *Koordinationsstelle für Forschung am Menschen* of the Swiss Federal Office of Public Health’s portal for human research in Switzerland). This applies to research project involving human subjects that does not count as a clinical trial. Collection of health related personal data and biological specimens connected with minimal risk and stress.

The project is based on observational studies which include paper- and electronic questionnaire-based collection of health- and behaviour-related data, participatory observations, as well as the collection of capillary blood samples for the diagnosis of *Plasmodium* spp. infection and assessment of haemoglobin levels.

### Risks to participants

This is a minimal risk study. The main risk to participants is the risk of breach of confidentiality or privacy during the data collection or storage process. Since no particularly sensitive health or social data will be collected, the risk of social stigma in the unlikely event of data leakage is considered minimal.

A further risk is minor discomfort below every day risk when finger prick / heel-stick blood draws are taken. The puncture may cause a small amount of discomfort for a few minutes, slight bruising, irritation, or tenderness. The study staff will take precautions to prevent punctures that are too deep and use sterile methods to prevent the puncture from becoming infected.

All other assessments are non-invasive and do not constitute any physical risk to the study participants.

### Potential Benefits of the Proposed Research

The individual patient benefits from (1) access to safe pre-referral drugs, (2) being part of an extended continuum of severe malaria management, and (3) from an increased awareness of the health professionals about the potential consequences of severe malaria.

Beyond the individual benefit, the study will generate evidence on the responsible introduction of pre-referral QA RAS in a functioning severe malaria case management system and its results will be used to guide rational introduction of QA RAS at the peripheral level. An estimated 58 lives will be saved annually directly as part of the project (see assumptions and calculation in Table 7). After national-level scale-up, a total of 17,172 lives could be saved annually across the three project countries, and the total indirect impact in terms of lives that could be saved by rational use of RAS in all malaria-endemic countries could be approximately 34,000 deaths per year.

Results from this project will be published in international peer reviewed scientific journals and presented at relevant conferences, thus contributing to the dissemination of knowledge gained for the benefit of stakeholders involved in malaria research and control worldwide.

## Participant information and consent

Inclusion in the PSS will occur only if the participant’s parent or guardian has given preliminary informed consent at the CHW level and provided written informed consent at the referral health facility or during the Day 28 follow-up. Written informed consent will be obtained prior to data collection and all non-routine assessments, and prior to any trial-related activities being carried out. In the setting of this particular study, information and consenting tasks may be performed by CHW (preliminary informed consent only), research assistants including study field workers, study nurses or other designated study staff. All these individuals will undergo adequate training with a special emphasis on the process of unbiased information of study participants ensuring that non-participation in the study does not result in a child not receiving RAS or not being adequately managed. During this training, theoretical and practical sessions will be devoted to research ethics and obtaining informed consent.

Depending on the study activity, informed consent will be obtained on the following levels:

**Activity 1, patient surveillance system:**

Preliminary informed consent will be obtained by the community health worker or health worker at the primary facility from parents/guardians of all children < 5 years of age with a recent/current mild or severe febrile illness who sought care from community-based providers such as CHW or primary health facilities. Due to the emergency of the situation, this preliminary consent will be oral and will only cover inclusion into the PSS and blood sampling for mRDT, if the latter is not part of routine procedure. Later, a written informed consent for all patient enrolled in the PSS will be obtained by the study nurse either at the referral facility (in case of successful referral) or during the D28 FU home visit. In the case of patients brought directly to a referral facility, the full informed consent procedure will be performed as soon as adequate initial treatment has been provided to the patient. Consent will cover the collection of demographic and health data, blood sampling for Hb testing, mRDT and the conduct of structured interviews at the patient’s home during the day 28 follow-up. A separate written informed consent will be obtained prior to conducting a verbal autopsy in case of death of a child.

**Activity 2, health care provider survey:**

For the conduct of structured interviews, the research assistant will obtain written informed consent from community-based and referral facility health workers. Parents/guardians of children with a recent/current mild or severe febrile illness attending community-based health services will be asked for their consent to the medical consultation being observed by a field team member. Participants (health worker and parents/guardians) do not have to answer questions they do not want to and may terminate the interview or observed consultation at any time with no consequences for themselves or the participating children.

**Activity 3, household survey:**

Written informed consent for the conduct of structured interviews will be obtained from household heads and parents/guardians of children < 5 years. Participants do not have to answer questions they do not want to and may terminate the interview at any time with no consequences for themselves or their children.

**Activity 4, costing / cost-effectiveness study:**

Data relating to the costing of the management of suspected severe malaria will be collected as part of the health care provider and household surveys. No separate consent is necessary.

**Activity 5, routine monitoring of process indicators:**

Data collected under this activity includes coded / anonymised routine M&E data, mostly aggregate numbers, for which no consent is necessary.

The written information/informed consent documents will be available in the local language or a language understood by the participant or the participant’s parent/guardian, respectively. The translated informed consent forms in the relevant language(s) are included in the submission to the country’s IEC for approval.

An information sheet giving an overview of the study including the purpose, eligibility of participants, procedures, risks or inconveniences, benefits, compensation for participation, confidentiality and rights of the participants will be verbally presented to eligible participants / participants’ parents or guardians. A confidential environment during the information session will be sought. After the research assistant has fully explained the study, s/he will ask if the participant has any questions. If the participant does not have any questions and is willing, s/he should let the research assistant know whether or not s/he chooses to participate and will be asked to sign the written consent form. Both a copy of the information sheet and consent form will be left with study participants.

Eligibility for providing individual informed consent will follow the local law and customs in each country. On that basis, a minimum age for independently providing informed consent will be defined for each country (e.g. minimum 15 or 18 years).

Children and individuals considered minor by local law and customs and hence not eligible to independently provide informed consent will require the consent of one of their parents or a guardian. Individuals considered emancipated by local law may be able to consent without the permission, or even knowledge, of their parents / guardians.

In the case of illiterate participants or parents/guardians, an impartial witness may be required during the information session, depending on the regulations of the respective country. This applies to the full informed consent in the PSS and to the consent prior to a household survey interview. In such instances, the research assistant will explain to the participant / participant’s parent or guardian the information contained in the written document and ask whether he/she gives his/her consent to participate. Participant’s / participant’s parent’s or guardian’s consent will be confirmed by his/her fingerprint on the form whereas the witness will sign the form. The witness must be literate and must be present during the entire information session. The witness will sign the consent form to certify the completeness of the information given to the participant / participant’s parent or guardian, and its compliance with the written information in the participant information sheet.

Details of these procedures may be adjusted to the local regulations and customs, whereas all fundamental principles of informed consent must always be safeguarded.

## Participant confidentiality

The Investigators affirm and uphold the principle of the participant’s right to privacy and that they shall comply with applicable privacy laws. Especially, anonymity of the participants shall be guaranteed when presenting the data at scientific meetings or publishing them in scientific journals. Only core study personnel (members of the data management group, member of the coordination team, master and doctoral students involved in the project) will have access to the protocol, dataset, statistical code, etc. during and after the research project (publication, dissemination).

Confidentiality of all participants will be ensured through the replacement of any personally identifiable information (name, initials, birth dates etc.) with unrelated unique identifiers as soon as is practicable in the data management process. Original electronic databases will be stored on a password-protected ODK server at Swiss TPH. Codes for data will be stored safely on password-protected computers. Where relevant, names will be separated from the electronic data processed for analysis. Recorded interviews (audio-files) will be destroyed after transcription. The only identifiers used during data analysis will be a unique identification number.

All data will be kept under lock and key or on or password protected computers, with a change of passwords every three months for security reasons. Only key personnel will have access to this data.

Parents / guardians of children participating in PSS component will be asked for their contact details (name, address and at least one telephone number). This information ensures retrieval and follow-up of these children and will be kept strictly confidential. The Investigators will keep a separate confidential enrolment log that matches the unique identifier with the participants’ names and contact details.

Disclosure of any examination results (including biological results) will be restricted to the patient / parent or guardian.

All biological material collected in the frame of this study (mRDT and Hb microcuvettes) will be discarded immediately after obtaining the result of the tests.

All health related primary data will be locally stored as per local requirements and ensuring participant confidentiality. Direct access to source documents will be permitted for purposes of quality control, audits or inspections.

## Participants requiring particular protection

The study participants will include children < 5 years of age that present to the CHW / peripheral health facility or the referral facility in an emergency situation. Pre-referral RAS roll-out is targeted to this age group due to their high vulnerability to severe malaria. Their inclusion in this project is therefore of central importance for generating results relevant for informing the roll-out of pre-referral RAS treatment. The informed consent process at the CHW / peripheral health facility will take into account the urgency of the situation.

## Damage coverage

It is assumed that the rectal artesunate products will be registered in all countries upon study start (observation of use in patients) and liability for the product lies with the producer and the health care provider (malpractice / negligence). Therefore, and because of the existing guidance covering the use of the products in all countries implementation by the partner UNICEF has no experimental character. The study described in this protocol is of purely observational character, has no experimental components and does not assess product safety or efficacy.

In the unlikely case that registration of a product should be delayed, an exemption and import permit will be requested from the respective competent authority. In case a country should in such case request particular study insurance, respective provisions will be made after detailed consultation on what bases and data claims could be filed.

## Participant compensation

Participants will not be paid any compensation to participate in the study.

CHWs and health workers will be reimbursed for their efforts relating to patient care and data collection according to applicable local guidelines under the responsibility of the local health authorities. There will be no study-specific compensation.

## Other aspects

In the event that a study specific activity reveals a disease or health condition in the children that are subject to this study, the investigators will take all necessary measures to refer the concerned child to the appropriate health services according to the local directives.

# Quality control and quality assurance: description of measures

## Risk management

The main risk to the study results from data incompleteness and poor quality leading to inconclusive or even rejected study results. This risk will be managed by rigid quality control strategies including thorough trainings, routine data monitoring and yearly data quality audits. Furthermore, the dispersed data collection activities conducted by different study teams bears a risk to data confidentiality and data consistency.

Community-based pre-referral treatment bears the risk that referral advices are not strictly followed by care takers. This risk will be mitigated by emphasizing on the importance of immediate referral to an appropriate health facility, adequate medical care and full treatment with ACTs for severe malaria during community sensitization campaigns. Any signal of accumulating non-adherence to these treatment guidelines in the context of the CARAMAL study will be reported to the responsible health authorities to decide on necessary contingency actions.

The sample size calculation for the PSS is based on assumptions on incidence rates and case fatality rate of severe malaria and anticipated impact of the intervention. All these assumptions may be inaccurate due to the paucity of available data and may therefore have to be adjusted as the project is rolled out and evidence on these indicators is generated. Considering the potentially life-saving role of QA RAS, the baseline data collection will not be extended in case the minimum sample size could not be achieved within 6 months; however, this may only be critical for the Case Fatality Ratio assessment and will not affect evaluation of all other indicators listed in Table 1 for which a smaller sample size will still yield sufficient power.

The proposed study falls under risk category A according to the Swiss Federal Office of Health: collection of health related personal data and biological specimens connected with minimal risk and stress. Hemoglobin determination will be done on day 28 since there is a lack of data about a potential adverse drug reaction of artesunate. A number of cases of delayed haemolytic anaemia have been identified following treatment of severe malaria with injectable artesunate [[22-25]. None of these events have been life threatening and all were self-limiting [21-24]. The underlying mechanism of its development is not fully understood but the reaction has been observed particularly in patients presenting with hyperparasitaemia. In order to monitor information about these events with a minimum interaction with the observational character of the study, haemoglobin (Hb) levels in the studied children Hb levels will be measured after treatment initiation at the referral facility as well as during the D28 follow-up visit. Appropriate management (like onsite drug therapy or referral to a health facility if necessary) of any alarming result at day 28 will be ensured.

To mitigate the risk of any persisting malaria infection going undetected after treatment, an additional mRDT and/or parasitological malaria test will be performed at the day 28 follow-up visit.

Systematic safety monitoring of RAS and other antimalarials is outside of the scope of this project. This is an observational project on the utilisation of RAS in the context of severe malaria management. All drugs are registered in the study countries and safety monitoring and reporting falls under the countries’ routine pharmacovigilance system.

### Risk identification, assessment and mitigation

Risk identification and assessment will be done on a continuous basis. Any information gathered by the study team and considered a risk to the project, the individual participant or to the integrity of data will be assessed for probability of occurrence (high, medium or low) and impact (high, medium or low). Based on these assessments, each risk will be classified as critical (needing immediate response and monitoring), medium (needing a response plan that is put in action if deemed necessary) and low (no action required at this point). The response strategies to identified risks may include acceptance of the risk, risk mitigation and/or a risk contingency plan.

A risk management log will be generated for this project and maintained on an ongoing basis to document and track each risk.

## Quality control

To ensure consistency and progress of data collection and comparability of results across sites, in each implementation country, Swiss TPH will perform regular data quality audits (DQAs) on data collected by UNICEF as well as by the in-country partner research institutions. DQAs include verification of completeness, consistency and correctness of (transcribed) data, traceability of data, integrity and confidentiality of data, plausibility checks, adherence to guidelines / procedures, staff qualifications, etc. The results of the DQA will be used to identify activities for improving quality.

In addition, Swiss TPH’s oversight plan foresees regular visits to the research sites to assure quality of in-country partner’s research activities including data capture and management as well as the appropriate use of the data collection tools. This includes adherence to study procedures and applicable guidelines as well as spot-checks for accuracy of transcription of source data.

Swiss TPH will also conduct remote real-time monitoring of data (completeness, consistency, timeliness, trends, inter-site comparisons etc.).

## Translations - Reference language

The study protocol, the Informed Consent Forms and the Data Collection Tools were developed in English (master documents), all further language versions are translations of these documents. Translations into local languages were done by a native speaker of the respective language and were double-checked by a second native speaker. Translations of the ICFs and data collection forms were back-translated into English to ensure consistency.

# Dissemination of results and publication policy

The target audiences for the proposed research include the WHO, the Ministries of Health and particularly the National Malaria Control Programmes (NMCP) of Nigeria, DRC and Uganda, as well as the wider malaria control and research communities.

Regular exchange of preliminary study findings with the NMCPs and WHO will be ensured and dissemination of evidence created through the project will be compiled in technical documents so that countries with RAS in their guidelines are informed on best practices for use and implementation, and that malaria partners can advocate for the rational use of RAS on a global level.

Study findings will also be submitted for publication in peer-reviewed scientific journals and presented at scientific conferences to inform the wider scientific community. Joint authorship of all publications arising from this project will be sought, involving institutions and individuals actively involved in this project, and following the recommendations issued by the International Committee of Medical Journal Editors [26].

In accordance with the Helsinki declaration, dissemination of study results to research participants and their communities is considered essential and an ethical requirement to engage communities in research activities. Appropriate strategies that are in accordance with local malaria control policies will be agreed upon among the consortium partners; community sensitization campaigns by UNICEF and local partner organizations will be one leveraged platform for dissemination.

# Funding and support

This project is funded by Unitaid. The research team attests that there is no conflict of interest and that our study will be conducted independently of outside influences in terms of specific intellectual, financial, and proprietary agendas.

# References

1. WHO, *World Malaria Report*. 2016, World Health Organization.

2. WHO, *Guidelines for the treatment of malaria*. 2015, World Health Organization.

3. Gomes, M.F., et al., *Pre-referral rectal artesunate to prevent death and disability in severe malaria: a placebo-controlled trial.* Lancet, 2009. **373**(9663): p. 557-66.

4. Gomes, M., et al., *Rectal artemisinins for malaria: a review of efficacy and safety from individual patient data in clinical studies.* BMC Infect Dis, 2008. **8**: p. 39.

5. Tozan, Y., et al., *Prereferral rectal artesunate for treatment of severe childhood malaria: a cost-effectiveness analysis.* Lancet, 2010. **376**(9756): p. 1910-5.

6. Habicht, J.P., C.G. Victora, and J.P. Vaughan, *Evaluation designs for adequacy, plausibility and probability of public health programme performance and impact.* Int J Epidemiol, 1999. **28**(1): p. 10-18.

7. Marsh, K., et al., *Indicators of life-threatening malaria in African children.* N Engl J Med, 1995. **332**(21): p. 1399-404.

8. Dondorp, A., et al., *Artesunate versus quinine for treatment of severe falciparum malaria: a randomised trial.* Lancet, 2005. **366**(9487): p. 717-25.

9. Dondorp, A.M., et al., *Artesunate versus quinine in the treatment of severe falciparum malaria in African children (AQUAMAT): an open-label, randomised trial.* Lancet, 2010. **376**(9753): p. 1647-57.

10. WHO, *Global technical strategy for malaria 2016-2030*. 2015, World Health Organization.

11. WHO-UNICEF, *Integrated Community Case Management (iCCM): An equity-focused strategy to improve access to essential treatment services for children*. 2012.

12. WHO, *Verbal autopsy standards: The 2016 WHO verbal autopsy instrument*. 2016.

13. Drummond, M., et al., *Methods for the economic evaluation of health care programmes*. Third ed. 2005, Oxford, United Kingdom: Oxford University Press.

14. World Health Organization, *Good practices for selecting and procuring rapid diagnostic tests for malaria*. 2011, Geneva: World Health Organization.

15. World Health Organization, *Malaria rapid diagnostic test performance: results of WHO product testing of malaria RDTs: round 7 (2015-2016)*. 2017, Geneva: World Health Organization.

16. Kabuya, C., et al., *Routine data for disease surveillance in the undeveloped region of the OR Tambo district of the Eastern Cape Province.* Stud Health Technol Inform, 2014. **197**: p. 103-7.

17. Raja, A., et al., *Android and ODK based data collection framework to aid in epidemiological analysis.* Online J Public Health Inform, 2014. **5**(3): p. 228.

18. Ferrari, G., et al., *An operational comparative study of quinine and artesunate for the treatment of severe malaria in hospitals and health centres in the Democratic Republic of Congo: the MATIAS study.* Malar J, 2015. **14**: p. 226.

19. Fleiss, J., B. Levin, and M. Paik, *Statistical Methods for Rates and Proportions.* 3rd ed. 2003, New York: Wiley.

20. WHO, *Severe malaria.* Trop Med Int Health, 2014. **19 Suppl 1**: p. 7-131.

21. Hetzel, M.W., et al., *Obstacles to prompt and effective malaria treatment lead to low community-coverage in two rural districts of Tanzania.* BMC Public Health, 2008. **8**(1): p. 317.

22. Burri, C., et al., *Delayed anemia after treatment with injectable artesunate in the Democratic Republic of the Congo: a manageable issue.* Am J Trop Med Hyg, 2014. **91**(4): p. 821-3.

23. Centers for Disease, C. and Prevention, *Published reports of delayed hemolytic anemia after treatment with artesunate for severe malaria--worldwide, 2010-2012.* MMWR Morb Mortal Wkly Rep, 2013. **62**(1): p. 5-8.

24. Rolling, T., et al., *Delayed hemolysis after treatment with parenteral artesunate in African children with severe malaria--a double-center prospective study.* J Infect Dis, 2014. **209**(12): p. 1921-8.

25. WHO/GMP, *WHO Information Note on Delayed Haemolytic Anaemia following Treatment with Artesunate*. 2013.

26. ICMJE, *Recommendations for the Conduct, Reporting, Editing, and Publication of Scholarly Work in Medical Journals*. 2016.

27. Krishna, S., et al., *Bioavailability and preliminary clinical efficacy of intrarectal artesunate in Ghanaian children with moderate malaria.* Antimicrob Agents Chemother, 2001. **45**(2): p. 509-16.

28. Morris, C.A., et al., *Review of the clinical pharmacokinetics of artesunate and its active metabolite dihydroartemisinin following intravenous, intramuscular, oral or rectal administration.* Malar J, 2011. **10**: p. 263.

29. Price, R., et al., *Adverse effects in patients with acute falciparum malaria treated with artemisinin derivatives.* Am J Trop Med Hyg, 1999. **60**(4): p. 547-55.

30. Idro, R., et al., *Cerebral malaria: mechanisms of brain injury and strategies for improved neurocognitive outcome.* Pediatr Res, 2010. **68**(4): p. 267-74.

1. Rectal artesunate product characteristics and dosage

RAS will be procured as suppositories containing 100 mg of artesunate. In addition to meeting the WHO PQ quality criteria, the product will require regulatory registration / import licencing or a specific waiver in each of the RAS project countries before delivery can be initiated.

Parasite clearance kinetics is comparable between intravenous and rectal artesunate [27] with parasitaemia reduced by >60% after 12 hours, and at 24 hours > 90% with single-dose (10 mg/kg) RAS [4]. As compared to oral artesunate, RAS exhibits a slightly shorter time to maximal plasma concentration - Tmax [28]; this may be attributable to the fact that RAS does not have to pass through the liver, leading to a higher bioavailability after administration.

The safety profile of rectal artemisinins is favourable and consistent with that of the artemisinins in general [4]. Fever, headache and unspecified pain as well as gastrointestinal problems (vomiting, nausea, diarrhoea, constipation, abdominal pain) were described to be related to administration of rectal artemisinins (including artesunate and artemisinin suppositories) [4]. More rarely, dizziness and hearing impairment were reported. Neurotoxicity has been observed in animals associated with prolonged exposure and was not observed with single-dose administration of artemisinin derivatives [4, 29]. No clinically relevant drug-drug interactions between artesunate and other drugs have been reported.

The risk associated with RAS under-dosing is considered more important due to the acute danger of severe malaria, whereas the risk of overdosing is obviously limited by the single-dose regimens recommended by WHO.

A rapid amelioration of the symptoms of severe malaria after RAS administration bears the risk that the child is not referred and sub-optimally treated at the community level with the possible consequence of not fully recovering and suffering long-term neurological and cognitive deficits or epilepsy [30].

**Dosage plan**

In the context of the Unitaid-supported pilot roll-out, suppositories containing 100 mg of artesunate will be used:

- children ≤10 kg body weight: 1 x 100 mg
- children > 10 kg and ≤ 20 kg body weight: 2 x 100 mg

This dosing scheme follows a rather conservative approach in that children will receive at least the minimal recommended dose. It is the WHO’s perspective that, as severe malaria is a life-threatening medical emergency, children should rather be over- than under-dosed.

1. Country profiles

**Democratic Republic of the Congo (DRC)**

***Malaria situation***

DRC is the second largest and third most populous country in Africa and all of its 81.3 million people (2016) are at risk of contracting malaria. Malaria is the principal cause of morbidity and mortality, accounting for more than 40% of all outpatient visits and for 19% of deaths among children under five years of age. Given that the majority of the population lives in high transmission zones, it has been estimated that the DRC accounts for 9% of all malaria cases and 10% of all malaria deaths, worldwide.

The National Malaria Control Strategic Plan (2016–2020) introduced the stratification of health zones based on parasite prevalence as measured by the 2013 DHS. This approach allows the National Malaria Control Plan to focus high-impact interventions in the areas that bear the greatest disease burden. According to the 2013 Demographic and Health Survey, progress is being made in key malaria interventions, such as insecticide-treated net (ITN) ownership and use, as well as mortality rates for children under five years of age, which fell by 30%.^[[7]](#footnote-8)^

| **Population (2016):** | 81.3 million^[[8]](#footnote-9)^ |
| --- | --- |
| **Population at risk of malaria (2014):** | 100%^[[9]](#footnote-10)^ |
| **Malaria incidence/1000 population at risk (2013):** | 295^[[10]](#footnote-11)^ |
| **Under-five mortality rate (2013):** | 104/1,000 live births^[[11]](#footnote-12)^ |
| **ITN use, children under 5 years (2013):** | 56%^10^ |
| **Current malaria policy document:** | Plan stratégique national de lutte contre le paludisme 2016-2020 |

***Health system***

On the peripheral level, the health system is organized in “health zones” (zone de santé) that cover a population of approximately 50’000 to 100’000 in rural areas and between 100’000 and 250’000 in urban areas. The coordination of the health zone lies with the central bureau. Each health zone has one General Referral Hospital that very often is not centrally located within the health zone leading to often long distances between the different health structures. The health zone has financial autonomy leading to a variable availability of drugs and maintenance and repair of facility and infrastructure.

The health zone is further sub-divided into “health areas” (aire de santé), each with a health facility covering 5’000 to 10’000 inhabitants in rural areas, and between 15’000 and 30’000 inhabitants in urban areas. These health facilities offer a minimal package of outpatient primary health care. They generally are staffed by one or two nurses. Referral health facilities/hospitals offer more comprehensive health care services.

According to the national policy, the population should be able to access health care within 5 km (or within one hour walking distance). Remote populations located at more than 5 km from the next health facility should have access to a community health site operated by volunteer community health workers. These sites operate according to the iCCM policy under the authority and supervision of the health zone (central bureau). Elected by the community, they receive a four-days training and continuous supportive supervision visits by the bureau central on a monthly basis. Their health care services cover children of 1 to 5 years of age. The CHW is not paid but receives a motivation fee. For patients, consultation and treatment are free of charge.

iCCM community health sites are supported by UNICEF and the Global Fund through financing and medicines supply (Global Fund covering malaria, UNICEF covering pneumonia and diarrhoea), and efforts are ongoing to increase the number of CHWs / community health sites. Within the country, SANRU (Soins de Santé Primaires en milieu Rural) is responsible for supply of donations from the national to the peripheral level.

In general, the first contact in search for medical care is at the community health site (if available), health post or health facility of the health area. However, in some cases a referral health facility may be the nearest facility offering medical care so patients may directly go there. Cases that cannot be managed at the health facility are referred to the next higher level health facility. A system of referral letters at the community level is implemented.

Despite the long distances between first point of contact and the referral health facilities, only the appointed referral hospitals are authorized to manage severe cases of malaria.

The national treatment guidelines recommend injectable artesunate as first-line drug for severe malaria; due to frequent stock-outs of injectable artesunate, intravenous quinine is often used as an alternative.

***Project Implementation Area***

The study area will comprise the health zones Kenge located in the province of the same name, as well as the health zones Ipamu and Kingandu located in Kwilu Province. The three health zones comprise a total population of approximately 620, 000, including an estimate of over 93,000 children under five years of age.

| **Zone de santé** | **Population (2017)** | **Population <5 years (estim. 2017)** | **iCCM providers (community health sites)** | **Primary HF** | **Referral HF*** |
| --- | --- | --- | --- | --- | --- |
| Ipamu | 209,717 | 37,749 | 6 | 21 | 7 |
| Kenge | 300,207 | 54,037 | 16 | 23 | 6 |
| Kingandu | 109,195 | 19,655 | 6 | 17 | 3 |
| Total | 619,119 | 111,441 | 28 | 61 | 16 |

*incl. centre de santé de reference, hospital and secondary hospital

Project interviews will be conducted in French, Lingala and Kikongo. Persons 18 years or older and emancipated minors as per local laws and customs are eligible to provide informed consent.

**Nigeria**

***Malaria situation***

Nigeria is the most populous country in Africa and all 186 million people (2016) are at risk of malaria. According to the 2015 Malaria Indicator Survey the prevalence of malaria in children under five years of age is 27% with wide regional differences. The duration of the transmission season ranges from year-round transmission in the south to 3 months or less in the north. WHO has estimated the total number of malaria cases at 61 million and the number of malaria deaths at 110,000 in 2015.^[[12]](#footnote-13)^ It has also been estimated that Nigeria accounts for 29% of all cases and 26% of all malaria deaths, worldwide. Almost all health and socioeconomic indicators in the south of the country are significantly better than in the north. For example, under-five mortality rates are about 1.5 times higher in some northern zones than in the rest of the country.

On the other hand, there has been progress in malaria control and Malaria Indicator Surveys found an increase in insecticide treated net use between 2010 and 2015 (29% to 44% in children under five years).^[[13]](#footnote-14)^

| **Population (2016):** | 186 million^[[14]](#footnote-15)^ |
| --- | --- |
| **Population at risk of malaria (2014):** | 100%^[[15]](#footnote-16)^ |
| **Malaria incidence/1000 population at risk (2013):** | 343^[[16]](#footnote-17)^ |
| **Under-five mortality rate (2013):** | 128/1,000 live births^[[17]](#footnote-18)^ |
| **ITN use, children under 5 years (2013):** | 44%^10^ |
| **Current malaria policy document:** | National Malaria Strategic Plan 2014-2020 |

***Health system***

The Nigerian public health care system is organised into primary, secondary and tertiary levels. The Local Government Areas (LGAs) are responsible for primary health care, the State Governments are responsible for providing secondary care while the Federal Government is responsible for policy development, regulation, overall stewardship and providing tertiary care. At primary care level, populations of 500-5000 people are serviced by a health post, and populations above >5,000 people by a primary health centre.

Although the Nigerian economy benefited from an oil boom in the past, economic growth of the past decade has not improved the welfare of the majority of the population nor has it reduced the high levels of poverty and inequality. It is estimated that the government spends less than 5% of the national budget on health. The LGA level is the least funded and least organised level of government and therefore has not been able to properly finance and organise primary healthcare, creating a very weak base for service provision at the primary care level.

The 2015 Malaria Indicator Survey reported that 66% of children with fever sought advice or treatment, yet only 30% went to the public sector. While treatment for malaria (and some other diseases) is supposed to be free in the public sector, medicines are often not available. Ancillary treatment (non-first line antibiotics) or additional unavailable material (e.g. gloves, infusion kits) have to be purchased from the private sector.

On the other hand, the private healthcare system is robust and provides care for a substantial proportion of the Nigerian population. However, the sector is not well regulated and access to private providers is heterogeneous with most providers concentrated in urban areas and southern Nigeria. Interestingly, the 2008 DHS found even to the poorest quintile, the private sector provides 72% of healthcare services. Among the private sector providers, pharmacies and patent medicine vendors (PMVs) play a critical role, jointly providing 39% of the services to children with fever in 2008, compared to public clinics 37%, private clinics 13%, and shops 7%. The heavy reliance on the private healthcare sector introduces case management and health surveillance challenges.

The majority of childhood deaths (62%) occur at home because of inaccessible or insufficient health care. The Nigerian iCCM policy that came into place in 2013 allows community-oriented resource persons (CORPs) to deliver treatment for a number of conditions closer to people’s homes. At an LGA level, there is a group of leaders assigned to support /supervise the CORPs.

CORPs are selected at a ratio of about 1:200 population. They are volunteers, resident of the local community and recommended by local stakeholders. Their tasks include patient assessment, classification and treatment for selected simple conditions, or referral. They perform mRDT and administer ACT (AL), oral antibiotics and ORS, and conduct community sensitization. CORPs are unpaid but may be incentivized for training or receive small incentives on a quarterly basis.

Each state and LGA has a Malaria Program Officer who oversees malaria activities in his or her area.

***Project Implementation Area***

The study area will comprise selected Local Government Areas (LGA) in Adamawa State in the North-Eastern Region of Nigeria. Illiteracy rates are high in Adamawa and there are security concerns due to Boko Haram, particularly in the northern parts of the State. In 2017, there was an observed decrease in referrals and increased case fatality due to Boko Haram related security threats.

The State comprises a total of 21 LGAs, 12 of which – in the southern part of the State – are considered low security risk. The entire state has a population of over 4 million people, including an estimated 1.2 million children under five years, of which 2.4 million including 760,000 children under five live in low-risk LGAs. For the purpose of the research project, two to four LGAs will be selected from the “green” LGAs taking into consideration the outcomes of the rapid assessment of referral facilities (mentioned in the protocol) and on discussions with key stakeholders in Adamawa State.

Adamawa State health services include 226 primary health facilities and 17 referral facilities, 9 of which are located in the low risk LGAs. Each primary health facility supervises at least 10 CORPs.

| **LGA** | **Population (2016)** | **Population <5 years (estim. 2016)** | **iCCM providers** | **Primary HF** | **Referral HF** |
| --- | --- | --- | --- | --- | --- |
| Demsa | 155,739 | 31,148 |  | 31 | 1 |
| Fufore | 381,651 | 76,330 |  | 75 | 1 |
| Ganye | 365,773 | 73,155 |  | 61 | 1 |
| Jada | 321,304 | 64,261 |  | 31 | 1 |
| 4 LGAs | 1,224,467 | 244,894 |  | 198 | 4 |
| Total Adamawa | 4,046,960 | 777,992 | 2,700 | 226 | 17 |

Project interviews will be conducted in English, Hausa and Fulfulde. Persons 18 years or older and emancipated minors as per local laws and customs are eligible to provide informed consent.

**Uganda**

***Malaria situation***

Uganda has the third highest number of *P. falciparum* infections in sub-Saharan Africa, and some of the highest reported malaria transmission rates in the world.^[[18]](#footnote-19)^ Malaria transmission is stable and perennial in most parts of the country, except in the highland areas, where there is low and unstable transmission with potential for epidemics. According to 2015 data from Uganda’s Health Management Information System (HMIS), malaria on average accounts for 34% of outpatient visits and 28% of hospital admissions, which amounted to over 3.5 million cases and 6,000 deaths in 2015. Seasonality of transmission is reflected in two seasonal peaks in the middle and towards the end of the year.^[[19]](#footnote-20)^

The Uganda Malaria Reduction Strategic Plan for 2014–2020 calls for a rapid nationwide scale-up of cost-effective preventative and curative interventions to achieve universal coverage. The Plan outlines three main goals to be achieved by 2020: 1) reduce annual malaria deaths from 2013 levels to near zero; 2) reduce malaria morbidity to 30 cases per 1,000 population; and 3) reduce malaria parasite prevalence to less than 7%.

HMIS data have recently shown a positive trend in malaria case management and MIS malaria prevalence in children under 5 years decreased from 42% in 2009 to 19% in 2014.

An upsurge in malaria was observed in 2015 in ten districts of Northern Uganda following their transition from IRS to universal coverage of ITNs and improved case management in 2014^21^. It includes the CARAMAL project districts of Apac, Oyam and Kole. The need for IV artesunate has gone up because of the increased number of complicated cases being seen and the roll-out of rectal artesunate at community level is expected to further prevent the progression to complicated malaria. In response, the NMCP and partners provided technical assistance to the affected districts, health facilities, and communities.^[[20]](#footnote-21),^^[[21]](#footnote-22)^

| **Population (2016):** | 38.3 million^[[22]](#footnote-23)^ |
| --- | --- |
| **Population at risk of malaria (2014):** | 100%^[[23]](#footnote-24)^ |
| **Malaria incidence/1000 population at risk (2013):** | 232^[[24]](#footnote-25)^ |
| **Under-five mortality rate (2011):** | 90/1,000 live births^[[25]](#footnote-26)^ |
| **ITN use, children under 5 years (2014-15):** | 74%^10^ |
| **Current malaria policy document:** | Uganda Malaria Reduction Strategic Plan 2014–2020 |

***Health system***

The Ugandan Ministry of Health has four levels of administration: national, regional, district, and county. The central level includes the National Directorate of Public Health of the Ministry of Health (which houses the NMCP), where national guidelines and norms are promulgated. The provision of health services has been decentralized with districts and health subdistricts playing a key role in the delivery and management of health services. The private system consists of private health practitioners, private-not-for-profit providers and the traditional and complementary medicine practitioners.

The formal health services are structured into national and regional referral hospitals, general hospitals, and health centers (HCs) IVs, IIIs, and IIs. HC IIs only provide outpatient care and community outreach services; HC IIIs provide basic preventive and curative care, and supportive supervision to the community and HC IIs under their jurisdiction.

In approximately 75% of districts, Village Health Teams (VHT; also labelled HC I) facilitate health promotion, service delivery, and community participation in access and utilization of health services. According to a 2015 MoH review, the VHT strategy has been implemented to varying degrees across the districts that have different levels of capacity to coordinate, train, and supervise VHT activities but have been hampered by a lack of funds. VHTs are linked to HC IIs for supervision.

UNICEF supports the implementation of iCCM through VHTs, in addition to providing commodities for iCCM in selected districts. Overall, there are more than 80 districts, which are being targeted for scale-up of iCCM in Uganda with support from various partners (Global Fund, UNICEF, DFID, RMNCH Trust Fund, CHAI, and others).

***Project Implementation Area***

The study area will comprise the districts of Apac, Kole and Oyam located in Uganda’s Northern Region. The project area is highly endemic for malaria and in recent years, outbreaks have contributed to a large number of cases. The three districts together have a population of approximately one million, including an estimated >175,000 children under five years of age.

A total of 29 health facilities provide health services to the population in the three districts, including 7 HC II, 17 HC III, 3 HC IV and 2 hospitals. HC IV and hospitals are referral facilities with inpatient wards.

| **District** | **Population (2014)** | **Population <5 years (estim. 2014)*** | **iCCM providers** | **Primary HF** | **Referral HF**** |
| --- | --- | --- | --- | --- | --- |
| Apac | 368,626 | 65,247 | 1,470 | 28 | 2 |
| Kole | 239,327 | 42,361 | 1,120 | 9 | 1 |
| Oyam | 383,644 | 67,905 | 2,076 | 24 | 2 |
| Total | 991,597 | 175,512 | 4,666 | 61 | 5 |

*Children under 5 represent 17.7% of total population, National Population and Housing Census, 2014^[[26]](#footnote-27)^

**incl. HC IV and hospital. Some, but not all, HC III also have an inpatient ward.

Project interviews will be conducted in English and Lango. Persons 18 years or older and emancipated minors as per local laws and customs are eligible to provide informed consent.

1. The recommended pre-referral treatment options for children < 6 years, in descending order of preference, are therefore intramuscular artesunate; rectal artesunate; intramuscular artemether; and intramuscular quinine. [↑](#footnote-ref-2)
2. Country-specific, depending on the level at which pre-referral QA RAS will be implemented [↑](#footnote-ref-3)
3. Regular training on diagnosis, treatment and referral following iCCM guidelines will be conducted by UNICEF and its implementation partners as part of their support to the iCCM roll-out in all three countries. [↑](#footnote-ref-4)
4. A general training on referral letters is already part of the iCCM training for CHWs. [↑](#footnote-ref-5)
5. Based on DHS survey data [↑](#footnote-ref-6)
6. Incidence rates and distribution of severe malaria remain difficult to assess due to the fact that malaria predominantly occurs in areas where diagnosis is weak and where a big percentage of malaria-related deaths in the community go unreported 20. WHO, *Severe malaria.* Trop Med Int Health, 2014. **19 Suppl 1**: p. 7-131.. [↑](#footnote-ref-7)
7. President’s Malaria Initiative, Democratic Republic of the Congo, 2016 [↑](#footnote-ref-8)
8. U.S. Census Bureau, International Data Base 2015 [↑](#footnote-ref-9)
9. WHO, World Malaria Report 2015 [↑](#footnote-ref-10)
10. WHO, World Health Statistics 2015 [↑](#footnote-ref-11)
11. Demographic Health Survey (DHS) 2013 [↑](#footnote-ref-12)
12. WHO, World Malaria Report 2016 [↑](#footnote-ref-13)
13. President’s Malaria Initiative, Nigeria, 2016 [↑](#footnote-ref-14)
14. U.S. Census Bureau, International Data Base 2015 [↑](#footnote-ref-15)
15. WHO, World Malaria Report 2015 [↑](#footnote-ref-16)
16. WHO, World Health Statistics 2015 [↑](#footnote-ref-17)
17. Demographic Health Survey (DHS) 2013 [↑](#footnote-ref-18)
18. Okello PE, Van Bortel W, Byaruhanga AM, Correwyn A, Roelants P, et al. (2006) Variation in malaria transmission intensity in seven sites throughout Uganda. Am J Trop Med Hyg 75: 219-225 [↑](#footnote-ref-19)
19. WHO, World Malaria Report 2015. Geneva: WHO; 2015. [↑](#footnote-ref-20)
20. President’s Malaria Initiative, Uganda, 2016. [↑](#footnote-ref-21)
21. President’s Malaria Initiative, Uganda Malaria Operational Plan FY 2017 [↑](#footnote-ref-22)
22. U.S. Census Bureau, International Data Base 2015 [↑](#footnote-ref-23)
23. WHO, World Malaria Report 2015 [↑](#footnote-ref-24)
24. WHO, World Health Statistics 2015 [↑](#footnote-ref-25)
25. Demographic Health Survey (DHS) 2011 [↑](#footnote-ref-26)
26. Uganda Bureau of Statistics, The National Population and Housing Census 2014 – Main Report, Kampala, 2016 [↑](#footnote-ref-27)
