# Supplementary material for: Health worker compliance with severe malaria treatment guidelines in the context of implementing pre-referral rectal artesunate in the Democratic Republic of the Congo, Nigeria, and Uganda: An operational study
Source: PLoS Med. 2023 Feb 21;20(2):e1004189. doi: 10.1371/journal.pmed.1004189 (PMC9990943; doi:10.1371/journal.pmed.1004189)
Supplement: S1 Table — (DOCX) [file pmed.1004189.s004.docx]

**S1 Table.** Summary characteristics of surveyed patients and exposure variables (subsample, post-implementation only).

|  | **DRC** | | **Nigeria** | | **Uganda** | |
| --- | --- | --- | --- | --- | --- | --- |
|  | **Community enrolments** | **RHF enrolments** | **Community enrolments** | **RHF enrolments** | **Community enrolments** | **RHF enrolments** |
|  | **N = 850** | **N = 823** | **N = 113** | **N = 308** | **N = 132** | **N = 1,223** |
|  | n (%) | n (%) | n (%) |  | n (%) | n (%) |
| **Age (years)** |  |  |  |  |  |  |
| < 1 | 199 (23.4) | 175 (21.3) | 13 (11.5) | 42 (13.6) | 25 (18.9) | 279 (22.8) |
| 1 - 2 | 427 (50.2) | 412 (50.1) | 70 (62.0) | 181 (58.8) | 79 (59.9) | 654 (53.5) |
| 3 - < 5 | 224 (26.4) | 236 (28.7) | 30 (26.6) | 85 (27.6) | 28 (21.2) | 290 (23.7) |
| **Sex** |  |  |  |  |  |  |
| Female | 415 (48.8) | 375 (45.6) | 77 (31.9) | 140 (45.5) | 63 (47.7) | 543 (44.4) |
| **Pre-referral RAS use** |  |  |  |  |  |  |
| yes | 781 (91.9) | 33 (4.0) | 46 (40.7) | 2 (0.7) | 109 (82.6) | 126 (10.3) |
| **Malaria test*** |  |  |  |  |  |  |
| positive (mRDT or blood slide) | 761 (89.5) | 750 (91.1) | 111 (98.2) | 281 (91.2) | 130 (98.5) | 1214 (99.3) |
| negative / not done | 89 (10.5) | 73 (8.9) | 2 (1.8) | 27 (8.8) | 2 (1.5) | 9 (0.7) |
| **Rainy season°** | 546 (64.2) | 518 (62.9) | 70 (62.0) | 187 (60.7) | 91 (68.9) | 770 (63.0) |
| **Drugs payable** | 438 (51.5) | 478 (58.1) | 77 (68.1) | 246 (79.9) | 14 (10.6) | 139 (11.4) |
| **Hospitalisation payable** | 458 (46.1) | 439 (53.3) | 17 (15.0) | 51 (16.6) | 6 (4.6) | 148 (12.1) |
| **Age caregiver (years)** |  |  |  |  |  |  |
| < 30 | 259 (30.5) | 309 (37.6) | 64 (56.6) | 169 (54.9) | 88 (66.7) | 779 (63.7) |
| ≥ 30 | 591 (69.5) | 513 (62.4) | 49 (43.4) | 139 (45.1) | 44 (33.3) | 444 (36.3) |
| missing | 0 (0.0) | 1 (0.1) | 0 (0.0) | 0 (0.0) | 0 (0.0) | 0 (0.0) |
| **Education caregiver** |  |  |  |  |  |  |
| Completed secondary education | 493 (60.0) | 498 (63.0) | 28 (32.2) | 71 (29.1) | 12 (9.1) | 147 (12.0) |
| Completed primary education | 161 (19.6) | 163 (20.6) | 13 (14.9) | 56 (23.0) | 62 (47.0) | 484 (39.6) |
| No education | 168 (20.4) | 129 (16.3) | 46 (52.9) | 117 (48.0) | 58 (43.9) | 592 (48.4) |
| missing | 28 (3.3) | 33 (4.0) | 26 (23.0) | 64 (20.8) | 0 (0.0) | 0 (0.0) |
| **Health Zone / LGA / District**** |  |  |  |  |  |  |
| Kenge DRC / Fufore NG / Kole UG | 261 (30.7) | 308 (37.4) | 28 (24.8) | 109 (35.4) | 43 (32.6) | 351 (28.7) |
| Kingandu DRC / Mayo Belwa NG / Oyam UG | 189 (22.2) | 87 (10.6) | 69 (61.1) | 91 (29.6) | 69 (52.3) | 662 (54.1) |
| Ipamu DRC / Song NG / Kwania UG | 400 (47.1) | 428 (52.0) | 16 (14.2) | 108 (35.1) | 20 (15.2) | 210 (17.2) |
| Number and column % of those with non-missing data, pooled, by country and by enrolment location; missing data rows are number and column %.  Abbreviations: DRC, Democratic Republic of the Congo; LGA, local government area; mRDT, rapid diagnostic test for malaria; RAS, rectal artesunate; RHF, referral health facility  * Severe malaria diagnosis was based on clinical assessment, diagnostic test result may or may not have been considered for the diagnosis  ° At time of admission; DRC: October - April; Nigeria: May - October; Uganda: April – October  ** Health zones in DRC (Kenge, Kingandu, Ipamu) / LGA in Nigeria (Fufore, Mayo Belwa, Song) / District in Uganda (Kole, Oyam, Kwania) | | | | | | |
