# Supplementary material for: Health worker compliance with severe malaria treatment guidelines in the context of implementing pre-referral rectal artesunate in the Democratic Republic of the Congo, Nigeria, and Uganda: An operational study
Source: PLoS Med. 2023 Feb 21;20(2):e1004189. doi: 10.1371/journal.pmed.1004189 (PMC9990943; doi:10.1371/journal.pmed.1004189)
Supplement: S2 Table — (DOCX) [file pmed.1004189.s005.docx]

**S2 Table.** Patient, provider, caregiver and facility correlates with antimalarial medication appropriateness according to the WHO malaria treatment guidelines (sensitivity analysis).

|  |  |  |  |  |  |  |
| --- | --- | --- | --- | --- | --- | --- |
|  | **DRC** | | **Nigeria** | | **Uganda** | |
|  | **aOR (95% CI) ^a^** | ***P* value** | **aOR (95% CI) ^a^** | ***P* value** | **aOR (95% CI) ^a^** | ***P* value** |
| **Patient variables** |  |  |  |  |  |  |
| **Age (years)** |  |  |  |  |  |  |
| < 1 | 1.49 (1.14 - 1.93) | 0.003 | 0.13 (0.01 - 1.76) | 0.08 | 1.04 (0.72 - 1.69) | 0.96 |
| 1 - 2 | 1.46 (1.20 - 1.78) | <0.001 | 0.68 (0.16 - 2.41) | 0.23 | 0.95 (0.74 - 1.28) | 0.70 |
| 3 - < 5 | Ref |  | Ref |  | Ref |  |
| **Sex** |  |  |  |  |  |  |
| Male | Ref |  | Ref |  | Ref |  |
| Female | 0.97 (0.84 - 1.12) | 0.70 | 0.46 (0.17 - 1.51) | 0.15 | 0.88 (0.74 - 1.05) | 0.15 |
| **Weight (kg)** |  |  |  |  |  |  |
| < 8 | 1.00 (0.75 - 1.32) | 0.99 | 2.15 (0.53 -8.76) | 0.28 | 1.21 (0.82 - 1.77) | 0.32 |
| 8 - 10 | 1.01 (0.84 - 1.22) | 0.92 | 1.28 (0.35 - 4.65) | 0.71 | 1.05 (0.82 - 1.38) | 0.66 |
| > 10 | Ref |  | Ref |  | Ref |  |
| **Administration of RAS** |  |  |  |  |  |  |
| no (post-implementation, community) | Ref |  | Ref |  | Ref |  |
| yes (post-implementation, community) | 2.19 (1.60 - 2.99) | <0.001 | 0.50 (0.02 - 9.77) | 0.98 | 0.42 (0.18 - 0.97) | 0.04 |
| NA (Pre-implementation, RHF) | 1.74 (1.14 - 2.66) | 0.01 | 2.89 (0.08 - 7.81) | 0.82 | 1.05 (0.59 – 3.60) | 0.42 |
| **Enrolment at community provider** |  |  |  |  |  |  |
| no | Ref |  | Ref |  | Ref |  |
| yes | 1.11 (0.83 - 1.50) | 0.72 | 1.39 (0.10 – 5.16) | 0.95 | 1.49 (0.80 – 2.61) | 0.22 |
| **mRDT / blood slide result** |  |  |  |  |  |  |
| negative/not done | Ref |  | Ref |  | Ref |  |
| positive | 0.98 (0.78 - 1.23) | 0.85 | 0.48 (0.24 - 1.44) | 0.17 | 2.27 (0.64 - 8.27) | 0.32 |
| **Provider variable** |  |  |  |  |  |  |
| **Costs incurred for** |  |  |  |  |  |  |
| Drugs | 0.82 (0.68 -0.99) | 0.03 | 1.03 (0.04 -15.4) | 0.63 | 0.25 (0.18 -0.33) | <0.001 |
| Hospitalisation | 1.20 (1.02 -1.42) | 0.03 | 1.50 (0.23 -9.91) | 0.89 | 4.27 (2.57 -6.93) | <0.001 |
| **Caregiver variable** |  |  |  |  |  |  |
| **Age (Years)** |  |  |  |  |  |  |
| <30 | 1.19 (1.00 -1.39) | 0.06 | 0.49 (0.32 -1.67) | 0.19 | 1.18 (0.93 -1.52) | 0.15 |
| ≥30 | Ref |  | Ref |  | Ref |  |
| **Other contextual factors** |  |  |  |  |  |  |
| **RAS implementation period (pre- vs. post-implementation)** | 6.22 (4.84 – 7.99) | <0.001 | 4.90 (0.56 - 42.7) | 0.16 | 1.02 (0.79 - 1.31) | 0.69 |
| **Health Zone / LGA / District**◊ |  |  |  |  |  |  |
| Kenge DRC / Fufore NG / Kole UG | 1.22 (0.99 -1.49) | 0.05 | Ref |  | Ref |  |
| Kingandu DRC / Mayo Belwa NG / Oyam UG | 0.70 (0.57 -0.87) | <0.001 | 1.50 (0.41 - 19.9) | 0.87 | 6.50 (3.83 -8.05) | <0.001 |
| Ipamu DRC / Song NG / Kwania UG | Ref |  | 10.5 (1.00 -72.1) | 0.05 | 1.76 (1.09 -3.81) | 0.02 |
| **Seasonality** (rainy season) | 1.01 (0.85 - 1.18) | 0.95 | 4.58 (0.95 - 22.2) | 0.11 | 0.71 (0.42 -0.97) | 0.003 |
| Abbreviations: aOR, adjusted odds ratio; CI, confidence interval; DRC, Democratic Republic of the Congo; mRDT, rapid diagnostic test for malaria; RAS, rectal artesunate  ^a^ adjusted Odds Ratios (aOR), 95% confidence intervals (CI) and p-value obtained from logistic models. Adjusted for covariates shown accounting for clustering at RHF level, missing data for weight were imputed by multiple imputation methods.  ◊ Corresponds to Health zones in DRC (Kenge, Kingandu, Ipamu*) / LGA in Nigeria (Fufore*, Mayo Belwa, Song) / District in Uganda (Kole*, Oyam, Kwania) (* = Ref) | | | | | | |
