# Supplementary material for: Health worker compliance with severe malaria treatment guidelines in the context of implementing pre-referral rectal artesunate in the Democratic Republic of the Congo, Nigeria, and Uganda: An operational study
Source: PLoS Med. 2023 Feb 21;20(2):e1004189. doi: 10.1371/journal.pmed.1004189 (PMC9990943; doi:10.1371/journal.pmed.1004189)
Supplement: S3 Table — (DOCX) [file pmed.1004189.s006.docx]

**S3 Table.** Antimalarial treatment administration and prescription compliance: number of doses of injectable antimalarials administered and follow-on ACT administration / prescription (subsample, post-implementation only)

|  |  |  |  |  |  |  |  |  |  |
| --- | --- | --- | --- | --- | --- | --- | --- | --- | --- |
|  | **DRC** | | | **Nigeria** | | | **Uganda** | | |
|  | **Community enrolments** | **RHF enrolments** | ***P* value (Chi2)** | **Community enrolments** | **RHF enrolments** | ***P* value (Chi2)** | **Community enrolments** | **RHF enrolments** | ***P* value (Chi2)** |
|  | n (%) | n (%) |  | n (%) | n (%) |  | n (%) | n (%) |  |
| **Administration of at least one dose of an inj. antimalarial**^1^ | **N = 850** | **N = 823** | 0.790 | **N = 113** | **N = 308** | 0.906 | **N = 132** | **N = 1,223** | 0.942 |
| Yes | 804 (94.6) | 776 (94.3) |  | 111 (98.2) | 302 (98.1) |  | 131 (99.2) | 1213 (99.2) |  |
| Artesunate | 773 (90.9) | 736 (89.4) | 0.298 | 111 (98.2) | 298 (96.8) | 0.420 | 131 (99.2) | 1200 (98.1) | 0.353 |
| Artemether | 0 (0.0) | 3 (0.4) | 0.078 | 0 (0.0) | 4 (1.3) | 0.224 | 0 (0.0) | 1 (0.1) | 0.742 |
| Quinine | 34 (4.0) | 39 (4.7) | 0.460 | 1 (0.9) | 2 (0.7) | 0.799 | 0 (0.0) | 17 (1.4) | 0.173 |
| **Number of doses of inj. antimalarial** | **N = 804** | **N = 776** | 0.649 | **N = 111** | **N = 302** | 0.643 | **N = 131** | **N = 1,213** | 0.234 |
| < 3 | 38 (4.7) | 33 (4.3) |  | 10 (9.0) | 23 (7.6) |  | 0 (0.0) | 13 (1.1) |  |
| ≥ 3 | 766 (95.3) | 743 (95.8) |  | 101 (91.0) | 279 (92.4) |  | 131 (100.0) | 1200 (98.9) |  |
| **In-hospital administration of at least one dose of an ACT after ≥ 3 doses of inj. treatment**^2^ | **N = 804** | **N = 776** | 0.216 | **N = 111** | **N = 302** | NA | **N = 131** | **N = 1,213** | <0.001 |
| Yes | 658 (81.8) | 616 (79.4) |  | 0 (0.0) | 5 (1.7) |  | 39 (29.8) | 567 (46.7) |  |
| **Administration / dispensing / prescription of at least one dose of an ACT after ≥ 3 doses of inj. treatment**^3^ | **N = 804** | **N = 776** | 0.295 | **N = 111** | **N = 302** | 0.212 | **N = 131** | **N = 1,213** | 0.379 |
| Yes | 663 (82.5) | 624 (80.4) |  | 46 (41.4) | 146 (48.3) |  | 130 (99.2) | 1191 (98.2) |  |
| Number and % of children receiving appropriate antimalarial treatment in compliance with WHO guidelines (type of drug and number of doses), pooled, by country and enrolment location  Abbreviations: ACT, artemisinin-based combination therapy; ALU, artemether-lumefantrine; ASAQ, artesunate-amodiaquine; DRC, Democratic Republic of the Congo; RHF, referral health facility  ^1^ More than one type of antimalarial may have been administered  ^2^ Appropriate antimalarials  ^3^ Treatment prescription compliance  ^4^ Only includes children with a prescription / dispensing of an ACT (ALU or ASAQ) to complete treatment at home, does not include ACT treatment during hospitalisation | | | | | | | | | |
