# Supplementary material for: Health worker compliance with severe malaria treatment guidelines in the context of implementing pre-referral rectal artesunate in the Democratic Republic of the Congo, Nigeria, and Uganda: An operational study
Source: PLoS Med. 2023 Feb 21;20(2):e1004189. doi: 10.1371/journal.pmed.1004189 (PMC9990943; doi:10.1371/journal.pmed.1004189)
Supplement: Attachment — Submitted filename: signorell.pdf [file pmed.1004189.s007.pdf]

This paper discusses a trial of treatment experience of young children with severe malaria in three countries in Africa.

I was asked for a statistical report and I interpret that to include all aspects of the design and conduct of the study.

Points of detail

Page 7 The missing indicator method of dealing with missing the information in covariates has been criticised (Vach and Blettner, 1998). If multiple imputation is not undertaken then the authors might at least carry out a complete cases analysis as a sensitivity analysis (which belongs in the supplementary material unless it shows a difference).

Page 8 The caption to Figure 1 is not self–standing. Telling us what pre-RAS and post–RAS mean would help as I assume that here they do not mean before and after the administration of RAS but rather before and after a policy change. If my re–writing is wrong then all the more reason for the authors to give us the explanation.

Page 9, Table 1 How do the same districts occur in all three countries? This seems totally bizarre to me. The same applies to Supplementary Table 1 as well.

Page 10 Just giving a p–value is not sufficient. Editors of health journals started calling for measures of effect size instead of p–values three decades ago (Langman, 1986; Gardner and Altman, 1986)

Page 11 If the numbers in Nigeria are small we could still see estimates and confidence intervals. Since we do not seem to be told the actual numbers we have no way of calculating them for ourselves either. Of course the confidence intervals will be wide but that is no reason to avoid them.

Page 11 The authors state 'No such differences were observed in the other two countries.' The overall pooled model shows that the difference was present overall (assuming it is adjusted for country which the table suggests it was) so this needs a test for interaction if the statement is going to be justified. Table 2 in fact uses three age groups not two and although I agree that the estimates for DRC are so close as to be indistinguishable is that true in the other countries? Bearing in mind the variation in precision here I would be cautious about any statement about country differences. I back–calculated the standard errors from

the figures in the table and the largest is about an order of magnitude greater than the smallest.

Page 12, Table 2 What do the estimates for country refer to in the columns for single countries? Surely you cannot adjust for country in a model which does not include any data from that country? This is similar to the point I made about Table 1.

Page 18 The authors state that 'This resulted in a low overall treatment compliance, leading to poor treatment outcomes and an increased risk of resistance development.' Since the authors do not seem to have actually measured what happened when the caregivers were made responsible for administering medication this statement cannot be left as it is. as far as I can see the authors did not measure treatment outcomes either so that consequent is not supported by their data analysis.

Supplementary Tables 1 and 4 The caption refers to rows reporting missingness but I cannot find them in the table

Points of more substance

Pooling

From Page 10 Figure 2 onwards the authors present pooled data. I would question the wisdom of presenting pooled results when it is clear that there are really substantial differences between countries. I really do not see the point of reporting 4.0, 43.9, and 65.7 as being 50.0. The same point can be made many times later in the paper. The discussion contains many speculative comments about the reason for the differences but surely the authors must have more data which could give us some factual background?

If pooled results are to be given then the public health question which they are designed to be an answer should be specified and the appropriate method for combing the samples used. For instance if the public health ministry in, say, Burkina Faso wanted to know what they might find in their setting then the current value which weights the samples by their size seems inappropriate and a simple average (37.9) might be better. Even better would be a prediction interval for the average which I suspect would be very wide.

Absolute versus relative frequencies

We really need more support for the percentages which are given throughout in terms of the actual numbers. This is important for the benefit of later

meta–analysts who otherwise have to back–calculate them where error may creep in.

Summary

Quite a few points for clarification and some more important issues about the presentation and interpretation.

Michael Dewey

References

M J Gardner and D G Altman. Confidence intervals rather than P values: estimation rather than hypothesis testing. *British Medical Journal*, 292: 746–750, 1986.

M J S Langman. Towards estimation and confidence intervals. *British Medical Journal*, 292:716, 1986.

W Vach and M Blettner. Missing values in epidemiological studies. In P Armitage and T Colton, editors, *Encyclopaedia of Biostatistics*, pages 2641–2654. Wiley, New York, 1998.
