# Supplementary material for: Health worker compliance with severe malaria treatment guidelines in the context of implementing pre-referral rectal artesunate in the Democratic Republic of the Congo, Nigeria, and Uganda: An operational study
Source: PLoS Med. 2023 Feb 21;20(2):e1004189. doi: 10.1371/journal.pmed.1004189 (PMC9990943; doi:10.1371/journal.pmed.1004189)
Supplement: Attachment — Submitted filename: Point-by-point responses to Reviewers feedback, 11.11.2022.docx [file pmed.1004189.s008.docx]

-----------------------------------------------------------
Requests from the editors:

1) We note that you conducted research or obtained samples in a foreign country. Did you consider including a local author as first or last author? If not, we recommend that you consider doing so in line with ICMJE's authorship requirements (<https://www.icmje.org/recommendations/browse/roles-and-responsibilities/defining-the-role-of-authors-and-contributors.html>). PLOS also has a parachute research policy which aims to promote collaboration and inclusivity in global health research. You are required to complete PLOS’ questionnaire on inclusivity in global research and submit it with your revised paper. The policy and questionnaire can be found at <https://journals.plos.org/plosone/s/best-practices-in-research-reporting>.

- Thank you for this remark. Our authors’ list is in line with the ICMJE guidelines. The first author has had a major contribution in the conception and design of the work as well as in the interpretation of the data. The work within the CARAMAL project resulted in a total of 13 manuscripts or published papers of which 5 have/will have a local first author and 1 a local last author.
- The PLOS policy and questionnaire is attached to our submission of the revised paper. The checklist is referenced as S2 in the main body of our manuscript.

2) Please revise your title according to PLOS Medicine's style. Your title must be nondeclarative and not a question. It should begin with main concept if possible. Please place the study design only in the subtitle (i.e., after a colon) and sample description before the colon. For example, ““Health worker compliance with severe malaria treatment guidelines in the context of implementing pre-referral rectal artesunate in the Democratic Republic of Congo, Nigeria and Uganda: an operational study”

- Thank you for this comment. We have revised the title accordingly.

3) Please include specific countries in the title instead of “three high burden countries”

- We have revised the title accordingly.

4) Abstract:
a) Background: The final sentence should clearly state the study question.
b) In the Methods and Findings, please include the study design, the target population, years during which the study took place, length of follow up, and main outcome measures.
c) Line 39, should read, “It included…”
d) Please include the important dependent variables that are adjusted for in the analyses.
e) Please include p values, as appropriate, in addition to 95% CIs.
f) Please ensure that all numbers presented in the abstract are present and identical to numbers presented in the main manuscript text.
g) In the last sentence of the Abstract Methods and Findings section, please describe the main limitation(s) of the study's methodology.

- Where applicable, we have made the necessary modifications in the revised manuscript.

5) At this stage, we ask that you include a short, non-technical Author Summary of your research to make findings accessible to a wide audience that includes both scientists and non-scientists. The Author Summary should immediately follow the Abstract in your revised manuscript. This text is subject to editorial change and should be distinct from the scientific abstract. Please see our author guidelines for more information: <https://journals.plos.org/plosmedicine/s/revising-your-manuscript#loc-author-summary>

- A non-technical Author Summary is attached to our submission of the revised paper.

6) Please include the relevant protocol or prospectively written document with your revised manuscript as a Supporting Information file to be published alongside your study and cite it in the Methods section. A legend for this file should be included at the end of your manuscript.

- We don’t believe that publishing the CARAMAL protocol along with this manuscript adds any benefit to the reader. The protocol was established for a list of research questions to be answered by this project, of which the compliance with post-referral treatment was just one. We recommend referring readers to the published overview paper by Lengeler, Burri et al. (2022) *PLOS Glob Public Health* that presents the study design and relevant operational aspects*.*

7) Please make sure that the Methods section transparently describes when analyses were planned, and when/why any data-driven changes to analyses took place, including those made in response to peer review comments-- should be identified as such in the Methods section of the paper, with rationale.

- We have added the necessary information in the Methods section (Statistical analysis). The final analyses were planned in January 2021, after completion of data collection. The analysis plan was informed by interim analyses on incomplete datasets undertaken to inform stakeholders and project funder during the execution of the project.

8) As requested by reviewers, please provide further context and detail regarding the accompanying CARAMAL project.

- Further details about the CARAMAL study were added to the Methods section. Furthermore, the objective of the research presented here and its results were more clearly put in relation with other findings made within the project (e.g. RAS effectiveness).

9) Please ensure that the study is reported according to the STROBE and include the completed STROBE checklist as Supporting Information. Please add the following statement, or similar, to the Methods: "This study is reported as per the Strengthening the Reporting of Observational Studies in Epidemiology (STROBE) guideline (S1 Checklist)."
a) When completing the checklist, please use section and paragraph numbers, rather than page numbers.

- Please find the STROBE Checklist as S1 included in the re-submission.

10) Did you consider adjustment of maternal factions like education, employment status, age – factors which may affect completion of treatment. If not, please highlight this as a limitation?

- Thank you for this valuable remark. We have rerun the model including caregiver age as additional predictor. The results are shown in the new Table 3. Unfortunately, the variable “highest level of education completed” had 51% missing data and was therefore not included (see respective comment in the methods section).

11) Please include line numbers after in the results and discussion

- Thank you for spotting this oversight. Line numbers are now included throughout the manuscript.

12) Table 1: under the subheading “Health Zone/ LGA/ District,” please explain what the names mean and which country representation they stand for, e.g., “Kenge….”

- The respective countries for the three health zones / LGAs / districts are now indicated within the table.

13) Instead of pre- and post-RAS, please refer to “pre- and post- intervention” period to avoid confusion

- We have followed your advice and replaced pre- and post-RAS by pre- and post-intervention throughout the document, and feel that it indeed is clearer now.

14) Please consistently provide both 95% CIs and p values in results and tables.

- Done where applicable.

15) Please state p <0.001, not p <0.000

- Thank you for spotting this oversight. P-values in Supplementary Table 2 were corrected accordingly.

16)	Please define all abbreviations in the tables e.g., mRDT, RAS

- All abbreviations are now defined in the tables and figure captions.

17) Figure 2 & 3: please explain the meaning of the bars and asterisk in the footnotes

- Upon review of the figures we felt that the bars and asterisks did not provide much valuable information, we have therefore removed them. Where important differences between groups are significant, we mention so in the text.

18) Please replace the term “compliance” with “adherence” where it is used to refer to treatment adherence.

- Throughout the manuscript, the term “compliance” refers to health workers following treatment guidelines when administering or prescribing antimalarial treatment, while “adherence” refers to patients/caregivers adhering to instructions for drug intake.

19) Given the limitations of the observational study design, in the discussion and conclusions, please remove
causal language such as “impact,” “effect,” and “leading to poor treatment outcomes ….” Refer to associations instead.

- Many thanks for this valuable comment. We have made the necessary adjustments (e.g. Conclusions).

20) Discussion: paragraph 7, line 7, should read “… single dose of RAS…”

- Many thanks for spotting this error, it was corrected accordingly.

21) Conclusion: Please provide specific implications for policy and clinical practice as substantiated by the results.

- We have added the sentence “In order to effectively integrate pre-referral RAS into the continuum of care for severe malaria, health system deficiencies need to be addressed and health worker compliance strengthened to ensure the provision of effective, life-saving post-referral treatment.” to highlight the implications of our results for policy and clinical practice.

22) Please remove the ‘Funding,” and “Conflict of interest” statements from the end of the main text. In the event of publication, this information will be published as metadata based on your responses to the submission form.

- Done.

23) References:
a) Please select the PLOS Medicine reference style in your citation manager. In-text reference call outs should be presented as follows noting the absence of spaces within the square brackets, e.g., "... services [1,2]."

- Done.

b) Please ensure that journal name abbreviations consistently match those found in the National Center for Biotechnology Information (NCBI) databases.

- Thank you, we have corrected the reference list accordingly.

https://journals.plos.org/plosmedicine/s/submission-guidelines#loc-references. e.g. PLoS Med.
c) Please ensure all weblinks are accessible and have access dates.

- Done.

-----------------------------------------------------------


Comments from the reviewers:

**Reviewer #1:** The paper describes one aspect of the CARAMAL project which has made a major negative impact on WHO policy. The paper is critical to understand why the project has provided counterintutitive results. The publication of the paper seems to me an urgent priority.

This paper has to deal with two major challenges:
We are only getting one slice of the Salami. The treatment compliance described in the paper under review is critical and relevant for the outcome of the project yet without knowing the project the reader may be baffled regarding its relevance. It is my understanding that the key paper by Hetzel et al is about to be published. To help the reader grasp the relevance of the paper the authors should summarize the key findings of the project i.e. increased mortality after RAS roll-out in the introduction and/or in a box regarding 'research in context'.

- Many thanks for this valuable remark. We believe that by highlighting health impact results and better linking this research to the other components of the CARAMAL project helps to put the different pieces into context and illustrate a more comprehensive picture of the various complex factors that influence whether a child can access appropriate treatment or not. We have accordingly elaborated on the referral completion findings and the lack of beneficial health impact of RAS in the introduction.

The second challenge are the major discrepancies in research findings between countries. If findings and (as the authors inform us) some of the methods between countries are so different it makes little or no sense at all to pool the findings. For the reader it would be more convenient being able just to look at one set of pooled summary data but that would provide a wrong impression. It is quite possible that not all readers of the journal are familiar with these statistical facts hence it may be worthwhile to explain why the country findings are reported separately? More importantly the authors can't resist to report repeatedly pooled data from the three study countries. It will be interesting to see whether the statistical reviewer considers this legitimate.

- We fully agree with reviewer’s comment and have removed the pooled results from all the tables and figures, as well as limited referring to pooled results to a minimum in the text.

Please state what kind of a study this is e.g. prospective, observational?

- Done, the kind of study was specified in the Methods section (observational).

**Abstract:** the conclusions are based on data from the project but not from data included in the paper. if the authors want to include these conclusions and I think they should, they also have to state the mortality findings of the caramal project in their abstract

- As described above, mortality findings are introduced in the introduction section.

**Methods** "Information on the use of pre-referral RAS was consolidated from different data sources through the different points of contact with the healthcare system (CHW, PHC, RHF) and from a caregiver's interview on day 28." please explain how this was done, i.e. data management? How were discrepancies dealt with?

- Information on the use of pre-referral RAS was consolidated from different data sources (enrolment register, RHF and day 28 data collection forms) through the different points of contact with the healthcare system (CHW, PHC, RHF) and from a caregiver’s interview on day 28.

**Definitions:** pls. explain what you mean by 'three dimensions'?

- The Definitions in the Methods section were rewritten in order to better distinguish general compliance (i.e. the correct medicines) from the stricter interpretation of compliance for which three doses of parenteral antimalarial need to be administered before ACT is provided either directly administered, or dispensed at discharged or a prescription is issued

**Statistics:** "Costs incurred to caregivers for hospitalisation or medication were analysed as provider-level predictors" the paper under review does not include data on costs.

- We agree with this comment, we did not collect costs, but asked caregivers whether any fees had to be paid at discharge (y/n). We have revised the sentence to read “Caregivers were asked a yes/ no question about whether they had to pay any fees either for hospitalisation or for medication; this was analysed as provider-level predictor.”

**Results:** there is a major drop out of participants from 14,911 provisionally enrolled, to 7,983 included in the analysis, and 3,449 with an 'update data collection form'. I find it hard to understand how much data are missing? Which data are in the updated form which are not in the other form and what are the implications for the generalizability of the data?

- The details of the analysis dataset and how this relates to the total number of children enrolled in the CARAMAL project are provided in Fig. 1. We have adapted this Figure and its caption to help readers to understand how we arrive at the numbers included in the analysis dataset.

The authors sometimes refer to compliance with treatment, a term I am familiar with and then they use 'compliant treatment'. I may not be the only one who is confused by 'compliant treatment'. It sounds wrong to me. I apologize if that is due to my ignorance.

- Many thanks for pointing this out. We were not aware of this confusing expression. We have rephrased the respective passages accordingly.

**Discussion:** the word 'obviously' in the sentence "Adequate post-referral management is obviously critical to ensure complete patient cure and avoid death and persisting disability." strikes me as downright cruel. I can't help wondering if this is so obvious why didn't the project assure compliance with the post-referral management? The flippant way the sentence is worded the authors may open an ethics quagmire very difficult to get out of.

- Many thanks for pointing out this very valid point, we have dropped the word “obviously” from the sentence.

The conclusions read well but are not based on the data presented in the paper. It is essential that the authors include these data in one form or another in this paper to allow the reader to understand what they mean.

- Many thanks for this remark. Throughout the manuscript, we now have stressed the possible implications of our findings regarding sub-optimal post-referral treatment compliance with the main outcome of the CARAMAL project, i.e. the lack of effectiveness of RAS under the current health system conditions.

**Tables:** the authors have moved all tables to the supplementary section. Why not include the four tables in the text?

- We very much appreciate this remark and have therefore moved former supplementary tables 2 and 3 to the main body of the manuscript.

**Figures** should use colour.

- We agree and have used color to better illustrate the figures.

Figure 2: pls. revise the title of the y-axis. Patients treatment (%) is not informative.

- We agree with the reviewer’s comment and have relabeled the y-axis with Patients (%)

Figure 3B: pls. revise the legend to clearly indicate the difference between the two groups.

- Done. We relabeled the legend to clearly describe the two groups.

**Reviewer #2:** See attachment

Michael Dewey (see below)


**Reviewer #3:** Adequate and complete follow-up treatment at referral facilities is a critical factor in the context of implementation of pre- referral rectal artesunate. This requires strong health systems, including well-functioning referral facilities, both in terms of availability of trained health workers and commodities for the treatment of severe malaria. This paper presents findings on health workers' adherence to severe malaria treatment guidelines in referral facilities in the context of implementing pre-referral rectal artesunate.

The paper is well writtend. Below are specific comments.

1. Although the authors refer to the main paper for more detailed information on the methods for this manuscript, the components of the methods relevant to this substudy should be clearly described. How were RAS children tracked up to the study RHFs? What data collection tools were used at different time points: pre and post RAS? Exactly what supportive measures were in place for the deployment of RAS at the different sites to better understand the different contexts, etc.

- We appreciate these inputs and have addressed them in the revised methods section, to include all components of the patient surveillance system. At each of these points of contact we used an ODK-based data collection form

2. Page 5-6, line 105-106: "Patients provisionally enrolled at the community level were excluded from this analysis if the diagnosis of severe malaria was not confirmed in the RHF." The authors described the signs and symptoms of severe malaria in the inclusion criteria for community-level workers. But for severe patients in the RHF, how was severe malaria diagnosed? Were laboratory tests (RDT or microscopy) performed to confirm malaria? What was the national guideline for diagnosing severe malaria that RHF staff in different countries should follow? These need to be described for each country based on their treatment guidelines.

- Diagnosis of severe malaria was done by the RHF clinicians and could not be verified independently for correctness / compliance with national malaria guidelines. Most of the children included in this study did have a positive mRDT or blood slide at least at some point during their hospital stay. The sentence was modified accordingly and the fact, that the diagnosis was not confirmed by the study team was highlighted as a limitation.

3. Page 6, lines 125-127: "General treatment compliance was computed for the entire study population, while a refined treatment assessment was done in a sub-population for which the updated data collection form was used." The terms "General treatment compliance" and "refined treatment assessment" need to be defined. Authors refer here also "updated data collection form" here. This implies that different data collection forms were used at different time points in the study. These need to be described in detail.

- We agree with the reviewer’s comment. We have rewritten the Definitions in the Methods section and modified the description in Figure 1 accordingly.
- The basic data collection form used at the beginning of the study was adapted to include more detail on drug type, route of administration and treatment durations. This is described in the Study procedures in the Methods section.

4. Page 10, lines 173-174: "Across the full study period, most of the children were treated with an injectable antimalarial (DRC 83.7%, Nigeria 93.6% and Uganda 94.8%; Supplementary Table 2)." To clarify the statement, change to "Across the full study period, most of the children were treated with an injectable antimalarial at RHFs (DRC 83.7%, Nigeria 93.6% and Uganda 94.8%; Supplementary Table 2)."

- We have revised the sentence accordingly.

5. Page 10, lines 173-179: In the statements "Across the full study period, most of the children were treated with an injectable antimalarial (DRC 83.7%, Nigeria 93.6% and Uganda 94.8%; Supplementary Table 2). In 86.8% of these cases, injectable artesunate was administered. During the post- RAS period, administration of parenteral antimalarials was higher (94.1%, artesunate 87.8%) than in the pre- RAS period (75.2%, artesunate 50.0%; Fig 2, Supplementary Table 2). In DRC, the use of intravenous quinine was still common (18.3% among all children) though it was gradually replaced by artesunate throughout study duration (34.6% pre- RAS, 10.3% post- RAS)." These data refer to all admitted severe patients in RHFs. It would be informative to provide data on parenteral treatment of RAS treated patients from the community.

- Thank you for this comment. We agree that (parenteral) treatment by providers other than RHFs is an important aspect that is not discussed in this manuscript. Here, we focus on post-referral treatment as it is foreseen by the health system: i.e. at designated referral health facilities. Assessing quality of post-referral treatment at lower-level providers that administer injectable treatment of offer alternative treatment options would be very valuable in the context of treating children unable to complete referral to a RHF.

6. Page 10, lines 194-196: "Throughout the full study period, 42.0% of the children received both an injectable and an ACT, i.e. compliant treatment, during admission (Supplementary Table 2). While only 2.7% of the children in Nigeria received oral follow-on treatment, 44.5% did so in Uganda and 50.3% in DRC." The term "compliant treatment" has a sligh .

- Many thanks for this comment. We have removed the reference to compliant treatment.

7. RAS is a relatively new intervention and requires a context-specific new community-level delivery approach. The authors report that the use of pre-referral RAS had a positive impact on whether a child received compliant treatment in DRC and a negative impact in Uganda. They linked this to supportive interventions in the DRC, including training of health workers at RHF, provision of artesunate injectables, etc. This underscores the need to not only provide sustained support to community-based providers, but also to strengthen referral facilities. The authors did not elaborate on what we can learn from the three contexts studied and what they might suggest to improve appropriate follow-up treatment following RAS treatment.

- Many thanks for this excellent comment. We have added the following to the Discussion: “In conclusion, prerequisites for provision of compliant treatment for severe malaria include the availability of the recommended drugs, in particular artesunate and ACTs. Further, health workers must know and comply with the treatment guidelines; any incentives nor non-compliant treatment (e.g. financial) should be counteracted. Sensitisation for the need to complete treatment even if symptoms have improved is key. If the ACT course cannot be completed at the RHF, drug dispensation seems more favourable than a prescription. And finally, mechanisms to monitor adherence of home treatment should be implemented to ensure adequate treatment completion (e.g. follow-up home visit by CHW).”

This paper discusses a trial of treatment experience of young children with severe malaria in three countries in Africa.

I was asked for a statistical report and I interpret that to include all aspects of the design and conduct of the study.

# Points of detail

**Page 7** The missing indicator method of dealing with missing the information in covariates has been criticised (Vach and Blettner, 1998). If multiple imputation is not undertaken then the authors might at least carry out a complete cases analysis as a sensitivity analysis (which belongs in the supplementary material unless it shows a difference).

- We agree with the reviewer’s comments, we have revised table 3, the reported results are based on complete cases. We also did a sensitive analysis based on multiple imputation of missing weight (not shown), and there was no difference in the results.

We have also revised the methods section:

“All models were based on complete cases. In addition, we also did a sensitivity analysis using multiple imputation methods for weight, which had 12.4% missing values of the total sample. There were no differences in the results and we therefore only report results based on the complete cases.”

**Page 8** The caption to Figure 1 is not self–standing. Telling us what pre- RAS and post–RAS mean would help as I assume that here they do not mean before and after the administration of RAS but rather before and after a policy change. If my re–writing is wrong then all the more reason for the authors to give us the explanation.

- We agree with reviewer’s comments, we have revised the caption for Figure 1 to provide a definition of pre-RAS and post-RAS period as defined in methods.

**Page 9, Table 1** How do the same districts occur in all three countries? This seems totally bizarre to me. The same applies to Supplementary Table 1 as well.

- Districts were only in Uganda, while health zones were in DRC and LGA in Nigeria. We have clarified this in the footnote. (** Health zones in DRC (Kenge, Kingandu, Ipamu) / LGA in Nigeria (Fufore, Mayo Belwa, Song) / District in Uganda (Kole, Oyam, Kwania))

**Page 10** Just giving a *p*–value is not sufficient. Editors of health journals started calling for measures of effect size instead of *p*–values three decades ago (Langman, 1986; Gardner and Altman, 1986)

- We agree with the reviewers comment, we have revised Figure 2 and remove all the p-values.

**Page 11** If the numbers in Nigeria are small we could still see estimates and confidence intervals. Since we do not seem to be told the actual numbers we have no way of calculating them for ourselves either. Of course the confidence intervals will be wide but that is no reason to avoid them.

- We agree with the reviewers comment. We have revised the results to add actual numbers, estimates and 95% CI

**Page 11** The authors state ‘No such differences were observed in the other two countries.’ The overall pooled model shows that the difference was present overall (assuming it is adjusted for country which the table suggests it was) so this needs a test for interaction if the statement is going to be justified. Table 2 in fact uses three age groups not two and although I agree that the estimates for DRC are so close as to be indistinguishable is that true in the other countries? Bearing in mind the variation in precision here I would be cautious about any statement about country differences. I back–calculated the standard errors from the figures in the table and the largest is about an order of magnitude greater than the smallest.

- Many thanks but this comment is no longer relevant because we have removed the pooled result.

**Page 12, Table 2** What do the estimates for country refer to in the columns for single countries? Surely you cannot adjust for country in a model which does not include any data from that country? This is similar to the point I made about Table 1.

- We have clarified this in the footnote. (** Health zones in DRC (Kenge, Kingandu, Ipamu) / LGA in Nigeria (Fufore, Mayo Belwa, Song) / District in Uganda (Kole, Oyam, Kwania))

**Page 18** The authors state that ‘This resulted in a low overall treatment compliance, leading to poor treatment outcomes and an increased risk of resistance development.’ Since the authors do not seem to have actually measured what happened when the caregivers were made responsible for administering medication this statement cannot be left as it is. as far as I can see the authors did not measure treatment outcomes either so that consequent is not supported by their data analysis.

- We agree with the reviewers comment. We have revised this sentence accordingly:” This resulted in a low overall treatment compliance, entailing a danger of poor treatment outcomes and an increased risk of resistance development.”

**Supplementary Tables 1 and 4** The caption refers to rows reporting missingness but I cannot find them in the table

- We agree with reviewer’s comment. We have revised the footnote and the caption, “missing data rows are number and column %” has been removed.

# Points of more substance

## Pooling

From Page 10 Figure 2 onwards the authors present pooled data. I would question the wisdom of presenting pooled results when it is clear that there are really substantial differences between countries. I really do not see the point of reporting 4.0, 43.9, and 65.7 as being 50.0. The same point can be made many times later in the paper. The discussion contains many speculative comments about the reason for the differences but surely the authors must have more data which could give us some factual background?

If pooled results are to be given then the public health question which they are designed to be an answer should be specified and the appropriate method for combing the samples used. For instance if the public health ministry in, say, Burkina Faso wanted to know what they might find in their setting then the current value which weights the samples by their size seems inappropriate and a simple average (37.9) might be better. Even better would be a prediction interval for the average which I suspect would be very wide.

- We agree with reviewer’s comment and have removed the pooled results from all the tables and figures, as well as limited referring to pooled results to a minimum in the text

## Absolute versus relative frequencies

We really need more support for the percentages which are given throughout in terms of the actual numbers. This is important for the benefit of later meta–analysts who otherwise have to back–calculate them where error may creep in.

- We agree with reviewers comments. We have revised the results reporting and included actual numbers and percentages in bracket

# Summary

Quite a few points for clarification and some more important issues about the presentation and interpretation.

Michael Dewey

# References

M J Gardner and D G Altman. Confidence intervals rather than P values: estimation rather than hypothesis testing. *British Medical Journal*, 292: 746–750, 1986.

M J S Langman. Towards estimation and confidence intervals. *British Medical Journal*, 292:716, 1986.

W Vach and M Blettner. Missing values in epidemiological studies. In P Armitage and T Colton, editors, *Encyclopaedia of Biostatistics*, pages 2641– 2654. Wiley, New York, 1998.
