# Supplementary material for: Health worker compliance with severe malaria treatment guidelines in the context of implementing pre-referral rectal artesunate in the Democratic Republic of the Congo, Nigeria, and Uganda: An operational study
Source: PLoS Med. 2023 Feb 21;20(2):e1004189. doi: 10.1371/journal.pmed.1004189 (PMC9990943; doi:10.1371/journal.pmed.1004189)
Supplement: Attachment — Submitted filename: Point-by-point responses to Reviewers feedback, 16.01.2023.docx [file pmed.1004189.s009.docx]

Dear Editor,

Thank you for the positive feedback on our manuscript entitled “Health worker compliance with severe malaria treatment guidelines in the context of implementing pre-referral rectal artesunate in the Democratic Republic of the Congo, Nigeria and Uganda: an operational study”.

We have gone through the remaining comments and have now made the necessary changes as requested.

We have provided a clean manuscript and a version with tracked changes, alongside a point-by-point response below.

We are looking forward to seeing this paper published.

Kind regards,

Aita Signorell

---------------------------------------------------------------------------------------------------------------------------

Requests from Editors:

Author Summary:
* Please shorten your Author Summary such that is contains 2-3 single sentence bullet points per question. Questions 1 and 2 can be trimmed to include only essential details and bullet points can be combined where appropriate.

* Please include headline numbers in the summary, including sample size and key findings.
* Please place your Author Summary in the main manuscript, after the Abstract and before the Introduction.

- Thank you for these requests. We have revised the Author Summary accordingly and placed it in the main manuscript.

Has the CARAMAL protocol been published as part of previous work? If not, we would request that the protocol is included in the Supporting Information, in the interest of full transparency (unless there are any legal or ethical concerns associated with this inclusion). If already published (e.g., as Supporting Information for a prior paper), please feel free to cite this publication instead.

- The CARAMAL protocol has not yet been published because it was established for a list of research questions of which the compliance with post-referral treatment was just one. The overall design of CARAMAL and the different research components and methodologies were published in an overview paper (Lengeler et al. Community access to rectal artesunate for malaria (CARAMAL): a large-scale observational implementation study in the Democratic Republic of the Congo, Nigeria and Uganda. PLOS Glob Public Health. 2022;2(9):e0000464. doi: 10.1371/journal.pgph.0000464). As pointed out earlier, we feel that publishing the protocol as Supporting Information adds no particular benefit to this manuscript. However, we will nevertheless submit it along with our revisions and would like to leave it up to the Editors to decide whether publishing it as Supporting Information is still requested.

Abstract Methods and Findings: Please include the important dependent variables that are adjusted for in the analyses.

- Done

---------------------------------------------------------------------------------------------------------------------------

Comments from Reviewers:

**Reviewer #1:** thank you for taking the time to address my suggestions.

- Thank you for your careful review and for acknowledging our revisions.

**Reviewer #2:** The authors have addressed my points but I have one remaining minor point. I think the multiple imputation performed as a sensitivity analysis should be shown and not reported as "not shown" it could go into the supplement obviously.

Michael Dewey

- Thank you for your time to go through the manuscript again.

As indicated in the manuscript, the results are the same as what we have reported. Therefore, we don't see the need to show this in the supplement, unless you think otherwise.

**Reviewer #3:** The authors addressed all the queries satisfactorily.

- Thank you for your positive feedback.
